# A novel human leiomyoma tissue derived matrix for cell culture studies

**DOI:** 10.1186/s12885-015-1944-z

**Published:** 2015-12-16

**Authors:** Tuula Salo, Meeri Sutinen, Ehsanul Hoque Apu, Elias Sundquist, Nilva K. Cervigne, Carine Ervolino de Oliveira, Saad Ullah Akram, Steffen Ohlmeier, Fumi Suomi, Lauri Eklund, Pirjo Juusela, Pirjo Åström, Carolina Cavalcante Bitu, Markku Santala, Kalle Savolainen, Johanna Korvala, Adriana Franco Paes Leme, Ricardo D. Coletta

**Affiliations:** Cancer and Translational Medicine Research Unit, Faculty of Medicine, University of Oulu, PO Box 5281, FI-90014 Oulu, Finland; Medical Research Center Oulu, Oulu University Hospital and University of Oulu, FI-90014 Oulu, Finland; Department of Oral and Maxillofacial Diseases, University of Helsinki, FI-00014 Helsinki, Finland; Clinical Department, Faculty of Medicine of Jundiai (FMJ), Jundiai, São Paulo SP-13202-550 Brazil; Department of Oral Diagnosis, Oral Pathology Division, Piracicaba Dental School, University of Campinas, Piracicaba, São Paulo SP-13414-903 Brazil; Center for Machine Vision Research, University of Oulu, FI-90014 Oulu, Finland; Biocenter Oulu, University of Oulu, FI-90014 Oulu, Finland; Faculty of Biochemistry and Molecular Medicine, University of Oulu, FI-90014 Oulu, Finland; Proteomics Core Facility, Biocenter Oulu, University of Oulu, FI-90014 Oulu, Finland; Department of Obstetrics and Gynecology, Oulu University Hospital and University of Oulu, FI-90029 Oulu, Finland; Department of Obstetrics and Gynecology, Tampere University Hospital and University of Tampere, FI-33521 Tampere, Finland; Laboratório Nacional de Biociências, LNBio, CNPEM, Campinas, SP-13083-970 Brazil

**Keywords:** Tumor microenvironment matrix, Invasion, Migration, Hanging drop, Colony formation, Spheroid formation, Capillary formation

## Abstract

**Background:**

The composition of the matrix molecules is important in *in vitro* cell culture experiments of *e.g*. human cancer invasion and vessel formation. Currently, the mouse Engelbreth-Holm-Swarm (EHS) sarcoma -derived products, such as Matrigel®, are the most commonly used tumor microenvironment (TME) mimicking matrices for experimental studies. However, since Matrigel® is non-human in origin, its molecular composition does not accurately simulate human TME. We have previously described a solid 3D organotypic myoma disc invasion assay, which is derived from human uterus benign leiomyoma tumor. Here, we describe the preparation and analyses of a processed, gelatinous leiomyoma matrix, named Myogel.

**Methods:**

A total protein extract, Myogel, was formulated from myoma. The protein contents of Myogel were characterized and its composition and properties compared with a commercial mouse Matrigel®. Myogel was tested and compared to Matrigel® in human cell adhesion, migration, invasion, colony formation, spheroid culture and vessel formation experiments, as well as in a 3D hanging drop video image analysis.

**Results:**

We demonstrated that only 34 % of Myogel’s molecular content was similar to Matrigel®. All test results showed that Myogel was comparable with Matrigel®, and when mixed with low-melting agarose (Myogel-LMA) it was superior to Matrigel® in *in vitro* Transwell® invasion and capillary formation assays.

**Conclusions:**

In conclusion, we have developed a novel Myogel TME matrix, which is recommended for *in vitro* human cell culture experiments since it closely mimics the human tumor microenvironment of solid cancers.

**Electronic supplementary material:**

The online version of this article (doi:10.1186/s12885-015-1944-z) contains supplementary material, which is available to authorized users.

## Background

Translational cancer research almost completely lacks human tissue *in vitro* models that mimic the natural tumor microenvironment matrix (TMEM). This also was partially fulfilled by our organotypic leiomyoma 3D solid disc model [1], which has been successfully used in numerous cancer invasion studies [2–7]. In this model, the hypoxic tumor matrix provides an authentic environment including *e.g*. fibroblasts, vessels, collagen fibers, laminins, glycoproteins, cytokines and proteases [8].

*Matrigel®* (*BD Biosciences*), the mouse Engelbreth-Holm-Swarm (EHS) tumor-derived commercial product [9], is widely used for *in vitro* adhesion, invasion and capillary formation assays [10, 11]. However, the tumor matrix of rodents clearly differs from the respective human TMEM [12]. These differences most likely affect human cancer invasion processes and underscore the need for soluble human TMEM products. In addition to classic Matrigel® other EHS tumor derived products are also available, such as ECM gel (Sigma), Cultrex® BME (Amsbio), Geltrex® (Gibco Life Technologies) and ECMatrix™ (Millipore). All these products have the same disadvantage for human studies; they are mouse tumor tissue homogenates that differ in composition from human TMEM.

Since collagens are the most abundant proteins in the extracellular matrix (ECM), gels from purified rodent collagens are commonly used to embed cells into 3D cultures [13, 14]. In organotypic 3D cultures, type I collagen derived from rat tail is probably the most abundant ECM mimicking matrix. Other commercially available ECM molecules, like fibronectin [15], fibrin [16] and hyaluronic acid [17], are also used for *in vitro* studies. In addition, synthetic ECM or peptide matrices are available from various manufacturers. However, one purified molecule, a mixture of them, or totally synthetic matrices do not adequately simulate the complex effects of natural ECM due to the obvious lack of hundreds of cytokines or protease cleavage sites identified in natural tumor ECM [18, 19]. Moreover, the excessive presence of one molecule or a mixture of basement membrane components rich in growth factors does not reflect the ECM composition synthesized by stromal cells. *In vivo* the combinations of multiple TMEM factors are important for cell-ECM interactions during cancer progression [20].

Three recent reports [21–23] use the term myogel for an extracellular matrix material that is derived from human, mouse, rat or pig normal skeletal muscles using procedures similar to those of Kibbey [9] for the preparation of EHS tumor extract. The myogel material was shown to be adipogenic [21, 23] and to support the *ex vivo* amplification of corneal epithelial cells [22]. Vivo Biosciences Inc. is marketing HuBiogel, an ECM gel derived from normal human amnion tissue containing laminin, collagen types I and IV, entactin, tenascin and heparan sulfate proteoglycan, but lacking endogenous growth factors (EGF, TGF-α, TGF-ß, FGF and PDGF) as well as MMP-2 and MMP-9 [24]. These commercial products and other human ECM matrices used in research are derived from normal tissues (skeletal muscle, amnion membrane, placenta) or are *in vitro*, cell culture -derived [MaxGel™ Human ECM (Sigma), AlphaMAX3D (Neuromics)].

Here we describe a novel product prepared from human uterus benign leiomyoma tumor tissue [1] according to the method described for the preparation of Matrigel® [9]. We formulated a solution/gel of the total protein extracts, characterized the protein contents, and compared with Matrigel® using a set of *in vitro* experiments. Based on the results we conclude that the tumor tissue solution/gel derived from human leiomyoma offers an excellent human TMEM tool for analyzing human carcinoma cells *in vitro*. This novel Myogel product, combined with low melting agarose (LMA), provides an accessible method for analyzing cancer cell invasive, adhesive or migratory properties; potential chemotherapeutic compounds; as well as to test capillary formation. Hence Myogel-LMA together with myoma discs could offer a practical human “TMEM tissue kit” for translational cancer research purposes.

## Methods

### Myogel preparation

Human uterus leiomyoma tissue (after taking samples for histopathological analyses) was received from the Oulu University Hospital, Department of Gynecology [1] and the Tampere University Hospital, Department of Gynecology after obtaining the patients’ written informed consent. The use of myoma tissue was approved by the Ethics Committee of both the Oulu University Hospital, and the Tampere University Hospital. Myogel was prepared according to the method described [9] for the preparation of EHS sarcoma derived Matrigel® (BD Biosciences), with minor modifications. Briefly, myoma tissue, frozen with liquid nitrogen, was ground to a powder with a CryoMill (Retsch, Haan, Germany), and 10 g of tissue powder was suspended in 20 ml of ice cold 3.4 M, pH 7.4 NaCl buffer. After centrifugation, the pellet was homogenized in another 20 ml of the same NaCl buffer using a T18 Ultra-Turrax (IKA®-Werke GmbH & Co. KG, Staufen, Germany). A T18 Ultra-Turrax was also used in all the following homogenizations. The protein concentration in each preparation was measured using a DC Protein Assay (Bio-Rad) according to the manufacturer’s instructions. The absorbance at 590 nm was measured using a Victor^3^V 1420 Multilabel Counter and Wallac 1420 Manager (Perkin Elmer Life and Analytical Sciences, Turku, Finland). The protein concentrations in various Myogel batches were diluted using cell culture media described in the cell culture -section (see below) to match those of Matrigel®. The Myogel solution was then stored in small (≤1 ml) aliquots at −20 °C.

### Gradient SDS-PAGE of Myogel and Matrigel® and proteomic analyses of Myogel

Samples (20 μg) of four different Myogel batches (preparation numbers 12, 15, 16 and 17) and their mixture, as well as four different Matrigel® (BD Matrigel Matrix, BD Biosciences, Cat. Number 354234) samples were loaded on a gradient (4 %, 8 %, 15 %) SDS-PAGE gel and separated using 15 mA for 90 min. A PageRuler Plus Prestained Protein Ladder (Thermo Scientific) was used as a molecular weight marker. Proteins were stained using Coomassie Blue and the gel was washed with elution buffer to remove excess staining. The gel was photographed over a stripping table.

For proteomic analysis, 10 μg and 20 μg of four different Myogel batches (preparation numbers 3, 4, 6, 9) were separated as above. The gel was viewed over a stripping table, and individual bands were cut and collected from the gel. After digestion (0.3 μg of trypsin was used/band) each band was resuspended with formic acid (see μl/band in the Additional file 1: Figure S1B) on three selected Myogel samples (preparations 3, 6, 9) and stored in −20 °C until gel digestion for mass spectrometry. The gel digestion of selected samples for mass spectrometry was done according to Hanna *et al*. [25] with some modifications.

#### Mass spectrometry analysis

An aliquot of 4.5 μl of the resulting peptide mixture was analyzed on an ETD-enabled LTQ Orbitrap Velos mass spectrometer (Thermo Fisher Scientific, Waltham, MA) connected to a nanoflow liquid chromatography (LC-MS/MS) instrument by an EASY-nLC system (Proxeon Biosystems, West Palm Beach, FL) with a Proxeon nanoelectrospray ion source. Peptides were separated with a 2–90 % acetonitrile gradient in 0.1 % formic acid using an analytical column PicoFrit Column (20 cm x ID75 μm, 5 μm particle size, New Objective, Woburn, MA) at a flow of 300 nl/min over 27 min. The nanoelectrospray voltage was set to 2.2 kV and the source temperature was 275 °C. All of the instrument methods were set up in the data-dependent acquisition mode. The full scan MS spectra (m/z 300–1600) were acquired in the Orbitrap analyzer after accumulation to a target value of 10^6^. The resolution in the Orbitrap was set to r = 60,000 and the 20 most intense peptide ions with charge states ≥ 2 were sequentially isolated to a target value of 5000 and fragmented in the linear ion trap using low-energy CID (normalized collision energy of 35 %). The signal threshold for triggering an MS/MS event was set to 1000 counts. Dynamic exclusion was enabled with an exclusion size list of 500, exclusion duration of 60 s, and a repeat count of 1. An activation q = 0.25 and activation time of 10 ms were used.

#### Data analysis

Peak lists (msf) were generated from the raw data files using the Proteome Discoverer software version 1.3.0.339 (Thermo Fisher Scientific) with the Sequest search engine and searched against the UniProt Human Protein Database (release July 11, 2012; 69,711 entries), with the following parameters: carbamidomethylation as the fixed modification (+57.021 Da), oxidation of methionine (+15.995 Da) as the variable modification, one trypsin missed cleavage and a tolerance of 10 ppm for precursor and 1 Da for fragment ions. All datasets were processed using the workflow feature in the Proteome Discoverer software, and the resulting search data were further analyzed in the software ScaffoldQ + v.3.3.1 (Proteome Software, Inc.). The scoring parameters (Xcorr and Peptide Probability) in the ScaffoldQ+ software were set to obtain a false discovery rate (FDR) of less than 1 %, using the number of total spectra output from the ScaffoldQ+ software, strict parsimony principle was enabled, and leucine and isoleucine were considered equal. A normalization criterion, the quantitative value, was applied to the spectral counts.

### Two-Dimensional Gel Electrophoresis (2-DE)

For the proteomic analyses two Myogel and one Matrigel® (BD Matrigel Matrix, BD Biosciences, Cat. Number 354234) samples were further purified by buffer exchange using an Amicon Ultra ultrafiltration unit with a 10 kDa cutoff (Millipore) and urea buffer (7 M urea, 2 M thiourea, 4 % [w/v] CHAPS, 30 mM Tris, pH 8.5). After that the protein samples were sonicated and centrifuged. The protein concentrations in the supernatants were determined in duplicate with a Bradford-based assay according to the manufacturer’s instructions (Roti®-Nanoquant, Carl Roth) with urea buffer as a control and aliquots were stored at −20 °C. For 2-DE, 100 μg of the protein solution of each sample was adjusted with rehydration urea buffer (7 M urea, 2 M thiourea, 4 % [w/v] CHAPS, 0.15 % [w/v] DTT, 0.5 % [v/v] carrier ampholytes 3–10, Complete Mini protease inhibitor cocktail (Roche)) to a final volume of 400 μl. In-gel rehydration with IPG strips (pH 4–7, 18 cm, GE Healthcare) was performed overnight. Isoelectric focusing (IEF) was carried out with the Multiphor II system (GE Healthcare) under paraffin oil for 55 kVh. SDS-PAGE was performed overnight in polyacrylamide gels (12.5 % T, 2.6 % C) with the Ettan DALT II system (GE Healthcare) at 1–2 W per gel and 12 °C. The gels were silver stained as described by Ohlmeier *et al*. [26] and analyzed with the 2-D PAGE image analysis software Melanie 3.0 (GeneBio).

### Zymography

Gelatinolytic enzymes in Myogel and Matrigel® (BD Matrigel Matrix, BD Biosciences, Cat. Number 354234) were detected by a zymography method using fluorescently labeled gelatin [27]. Prestained low-range SDS-PAGE Standards (Bio-Rad) as well as purified control MMP-2 and MMP −9 samples were loaded in adjacent wells. After electrophoresis, gelatinases were activated by incubating the gels with zymography buffer (50 mM Tris–HCl, 5 mM CaCl_2_, 1 μM ZnCl_2_, 0.02 % NaN^3^, pH 7.5) overnight at 37 °C. Gelatin degradation was visualized under long wave UV light and photographed using an AlphaDigiDoc® RT Gel Documentation System (Alpha Innotech, San Leandro, CA).

### Assessing the pH of Myogel and Matrigel®

For pH comparison, Matrigel® (BD Matrigel Matrix, BD Biosciences, Cat. Number 354234) was diluted with an equal volume of serum free medium, and the same amount of total protein for Myogel was obtained by diluting it 10 + 6 with serum free medium. The pH of both gels was measured at the beginning, after 17 h, and at the end of the 48 h experiment. The gels were incubated at 37 °C in a 5 % CO_2_ humidified cell culture chamber with or without HSC-3 cells (see below for the culture conditions) on top of the gels.

### Cell lines

Aggressive human oral tongue squamous cell carcinoma cell line HSC-3 (Japan Health Sciences Foundation, Japan) was cultured in a 1:1 DMEM/F-12 medium (Life Technologies) supplemented with 100 U/ml penicillin, 100 μg/ml streptomycin, 250 ng/ml fungizone, 50 μg/ml ascorbic acid and 0.4 μg/ml hydrocortisone (all from Sigma-Aldrich) and 10 % heat inactivated fetal bovine serum (FBS; Life technologies). HSC-3 cells labeled with GFP were generated by stable transduction with non-silencing GIPZ lentiviral shRNAmir control particles (pGIPZ vector contains GFP in order to track shRNAmir expression; Thermo Fischer Open Biosystems) with puromycin (Sigma-Aldrich) selection according to the manufacturer’s instructions. Nuclear histone-2B (H2B)-coupled mCherry expression vector pLenti6.2 V5/DEST (a gift from Dr. Cindy E. Dieteren, Department of Cell Biology, Radboud UMC, Netherlands) was introduced into the HSC-3 cells with cytosolic GFP labeling using lentivirus mediated infection and selected in culture media containing 5 μg/ml blasticidin-S (Merck Millipore). HSC-3 cells expressing cytoplasmic GFP and H2B-coupled mCherry were cultured in DMEM/F12 medium (Gibco/Thermo Fisher Scientific), 10 % heat-inactivated FBS (HyClone/Thermo Fisher Scientific), 100 U/ml penicillin, 100 μg/ml streptomycin (Sigma-Aldrich), 50 μg/ml L-ascorbic acid (Sigma-Aldrich) and 2 mM L-glutamine (Sigma-Aldrich). HSC-3 cells labeled with RFP were generated by stable transduction with commercial lentiviral particles containing a non-coding control sequence (Amsbio) and selected with puromycin. They were cultured as normal HSC-3 cells.

The SCC-9 cell line (American Type Culture Collection, ATCC) was maintained in DMEM/F-12 medium (Invitrogen) supplemented with 10 % FBS (Cultilab), 400 ng/mL hydrocortisone, and antibiotic/antimycotic solution (Invitrogen). SCC-9 cells were labeled with ZsGreen protein and implanted subcutaneously into the footpads of the left front limb of BALB/c nude mice and LN-1 and LN-2 cell lines with increased metastatic potential were derived by *in vivo* selection from axillary lymph nodes with metastatic cells as described earlier [6]. Both LN-1 and LN-2 cells were maintained in culture as SCC-9.

Normal oral gingival fibroblasts (GF) were established from palatal gingiva mucosa biopsies and cultured in DMEM medium (high glucose, GlutaMAX^TM^ and pyruvate) supplemented with 10 % FBS, 50 U/ml penicillin, 50 μg/ml streptomycin and 2.5 μg/ml amphotericin B (all from Gibco). After obtaining written informed consent, the palatal tissue biopsies were taken from healthy volunteers for another study to be used as a starting material for control fibroblast cell line cultures. The volunteer consent encompassed the use of obtained cell lines for other studies as well. The use of palatal tissue was approved by the Ethics Committee of the Helsinki University Hospital. The carcinoma associated fibroblast (CAF) cell lines were generated from fragments of tongue squamous cell carcinomas by using tissue explants [28]. They were cultured in DMEM medium supplemented with 100 U/ml penicillin, 100 μg/ml streptomycin, 50 μg/ml ascorbic acid, 250 ng/ml fungizone, 1 mmol/L sodium pyruvate (Sigma-Aldrich) and 10 % heat inactivated FBS.

Melanoma cell lines SK-Mel-25 and A2058 (ATCC) were maintained in RPMI medium (Invitrogen) supplemented with 10 % FBS (Cultilab) as described earlier [29]. Human umbilical vein endothelial cells (HUVEC, ATCC) were cultured in a 1:1 mixture of DMEM/F12 medium (Invitrogen) supplemented with 10 % FBS and 400 ng/ml hydrocortisone (Sigma-Aldrich).

All the cells were cultured in a humidified atmosphere of 5 % CO_2_ at 37 °C and passaged routinely using trypsin-EDTA (Sigma-Aldrich). The media were changed every 2–3 days. They were regularly tested and confirmed to be negative for mycoplasma infection using a MycoTrace PCR Detection Kit (PAA Laboratories GmbH). Cell line identity was not routinely performed.

### Adhesion assay

A cell adhesion assay was conducted to determine how many cells bind to Myogel compared to Matrigel® (BD Matrigel Matrix, BD Biosciences, Cat. Number 354234). In this assay, HSC-3 cells were cultured to subconfluence. Wells in a 96-well plate were coated for 24 h either with 100 μl of PBS, BSA (bovine serum albumin, 10 μg/ml, Sigma-Aldrich), Matrigel® or Myogel (two different batches). Matrigel® was diluted to 1:10 in PBS and Myogel was diluted to the same protein concentration. At the same time, the cell culture medium was changed to serum-free medium. The next day the excess liquids were removed and the culture plates were incubated with 100 μl/well of 0.1 % BSA for 2 h and washed with PBS. HSC-3 cells (6000) in 100 μl of serum-free medium were added to each well and the wells were incubated at 37 °C in a 5 % CO_2_ humidified atmosphere for 2 h. The non-adherent cells were rinsed off, and the remaining cells were fixed with 10 % trichloroacetic acid (TCA), stained with crystal violet and quantified using an ELISA reader at 540 nm.

Adhesion of GFs on top of Myogel was studied using 6-well plates coated at 37 °C in a 5 % CO_2_ humidified atmosphere with 0.62 mg/ml Myogel diluted with DMEM without supplements. After 2 h the excess liquids were removed and 150,000 GFs were added in their normal culture medium. The cultures were photographed with Olympus CKX41 inverted microscope (Hamburg, Germany) after 2.5 h and 9.5 h with 20 x magnification to record the morphology of the cells.

### Terminal deoxynucleotidyl transferase dUTP nick end labeling (TUNEL)

An 8-well Nunc™ Lab-Tek™ chambered slide (Thermo Scientific) was used for the experiment. Wells were coated with either rat tail collagen type I (BD Biosciences), one of three different batches of Myogel or an equal (50–50 %) mixture of collagen type I and one type of Myogel. A total of 150 μl of coating mixtures were prepared, with a final protein concentration of each gel mixture of 0.62 mg/ml, which were adjusted with the addition of culture medium. Two wells were not coated and were kept for positive and negative controls for the subsequent TUNEL assay. The Lab-Tek™ chamber slide was covered and placed in the incubator at 37 °C in 5 % CO_2_ for 2 h. Subsequently, 5000 CAFs were added on each well and the slide placed in the incubator overnight. On the following day, the wells were washed twice with PBS, air-dried for few min and fixed with freshly prepared 4 % (w/v) paraformaldehyde in PBS (pH 7.4) for 1 hour at RT. After 2 washes with PBS for 5 min each, the TUNEL assay was performed using a commercially available kit (In Situ Cell Death Detection Kit, Roche). At the end of the assay, samples were counterstained with DAPI for 10 min at RT. After coverslip mounting with a water-based medium, 100 cells were counted under a confocal microscope using the blue channel (405 nm) and apoptosis was detected using the green channel (488 nm).

### CAFs cultured within Myogel

Myogel for CAF embedding was prepared as described for collagen organotypic culture in Nurmenniemi *et al*. [1]. Briefly, a gel mixture including 8 volumes of Myogel (4.5 mg/ml), 1 volume of 10 × DMEM (Sigma Aldrich) and 1 volume of FBS with CAFs (final concentration 70,000 cells/ 400 μl gel mixture) was prepared on ice and 400 μl aliquots were added into the wells of a 48-well plate. After 40 min polymerization, 100 μl of complete CAF medium was added on the polymerized gels. The plates were incubated at 37 °C in a 5 % CO_2_ humidified atmosphere. Cells were fed twice a week and pictures were taken with an EVOS microscope (Advanced Microscopy Group, Bothell, WA) weekly for three weeks.

### Myogel as a supplement in soft agar colony formation -assay

For the soft agar mimicking assay, one percent sterile base low melting agarose (LMA, Sea Plaque Low Melting Agarose, Lonza) melted in PBS was mixed with 10x DMEM/F12, 100 % FBS to give a final 0.8 % agarose with 1x medium, 10 % FBS. One-half ml of the mixture was added to wells in a 24-well plate and allowed to solidify for at least 30 min in the laminar flow hood. 10 000 HSC-3 (H2B-GFP) cells in 50 μl of FBS were mixed with 50 μl of 10x DMEM/F12 and 0.4 ml of 0.5 % LMA (as a control, final agarose concentration 0.4 %) or with 50 μl of 10x DMEM/F12, 0.2 ml of 1.0 % agarose and 0.2 ml Myogel (final agarose concentration 0.4 %, final Myogel protein concentration 2.2 mg/ml). Myogel was centrifuged at 4000 rpm for 10 min prior to the procedure. The agarose mixture was gently mixed by swirling, and 0.5 ml was added on the top of the agarose base. The plates were incubated at 37 °C in a 5 % CO_2_ humidified atmosphere for 28 days. The cells were fed twice a week with 0.25 ml normal HSC-3 medium. Pictures of the colonies were taken with transmitted light and in the GFP & RFP channels in different objectives (10x, 20x & 40x) using an EVOS inverted microscope. Cells in colonies were calculated from the pictures and ImageJ software (Rasband, W.S., ImageJ, U.S. National Institutes of Health, Bethesda, Maryland, USA) was used to measure colony area.

### Hanging drop spheroid cultures in Myogel with low-melting agarose (Myogel-LMA)

The spheroids were formed according to published protocol [30]. 20 μl drops of the cell suspension (70,000 HSC-3 (H2B-GFP) cells per drop) suspended in DMEM/F12 medium with 10 % FBS were placed onto the lids of 10 cm dishes, which were inverted over dishes containing 10 ml PBS. Hanging drop cultures were incubated for 72 h, the resulting cellular aggregates were harvested by a pipette and embedded in two different conditions (Myogel-LMA & LMA) into the wells of a 48- well plate as in the soft agar colony formation –assay above. After 6 h incubation at 37 °C in a 5 % CO_2_ humidified atmosphere, 150 μl normal HSC-3 cell culture medium was added per well. Pictures of the spheroids were taken with 4x objective using an EVOS inverted microscope at 0 h, 24 h, 48 h and 72 h after embedding. ImageJ software was used to measure spheroid area and the results were calculated as a ratio to the area of the implanted spheroid right after embedding (without media).

### Microarray

For microarray analysis, 90,000 HSC-3 cells transduced with RFP were seeded into uncoated or Myogel coated 6-well plates (three wells each). The next day the cells were harvested for RNA extraction using a Qiagen RNA kit. Three samples of each group (on top of plastic or Myogel coating) were pooled; the pools contained an equal amount of RNA from each sample. Affymetrix GeneChip Human Genome U133 Plus 2.0 Arrays were used for microarray analysis and experimental procedures were performed according to the Affymetrix GeneChip Expression Analysis Technical Manual. Briefly, 1 μg of total RNA was used as a template to synthesize biotinylated cRNA by means of the GeneChip 3’IVT Express kit (Affymetrix) according to the manufacturer’s instructions. The cRNA was fragmented to 35 to 200 nt prior to hybridization to Affymetrix Human Genome U133 Plus 2.0 arrays containing approximately 55,000 human transcripts. The array was washed and stained with streptavidin-phycoerythrin (Molecular Probes). Finally, biotinylated anti-streptavidin (Vector Laboratories Inc.) was used to amplify the staining signal and a second staining was performed with streptavidin-phycoerythrin. The arrays were scanned on a GeneChip Scanner 3000 (Affymetrix, High Wycombe, United Kingdom). The expression data was analyzed to find genes with fold changes (FC) of 1.5 or more using dChip software [31]. The genes with FC 1.2 or more were divided into Gene Ontology (GO) categories using a dChip enrichment analysis tool.

### Scratch assay

To analyze the effects of Myogel and Matrigel® (BD Matrigel Matrix, BD Biosciences, Cat. Number 354234) on the migration of HSC-3 cells, 24-well plates were coated with 0.62 mg/ml Myogel (two different batches) or 0.62 mg/ml Matrigel®. The coating was left to solidify for 2 h at 37 °C and then washed twice with PBS. HSC-3 cells (90,000) were allowed to attach overnight, and were then wounded with a pipette tip, rinsed twice with PBS, post-coated for 1 h and rinsed before 1 % FBS medium was added. The wounds were photographed with an EVOS photomicroscope at 0 h and 24 h after scratching. The area of the open wound was measured using ImageJ software and the results were calculated as a percentage of wound closure.

### Transwell invasion through Myogel and Matrigel®

To compare Myogel with Matrigel® (BD Matrigel Matrix, BD Biosciences, Cat. Number 354234) as an invasion assay material, experiments were carried out according to BD Biosciences instructions to coat the filter membranes with Matrigel®. Fifty μl of either 1 + 1 Matrigel® or Myogel (three different batches at the same protein concentration as Matrigel®) diluted with serum-free medium was added to the upper chamber of a Transwell® nylon filter membrane insert (Corning Inc.), incubated at 37 °C in a 5 % CO_2_ humidified atmosphere for 30 min after which 50,000 HSC-3 cells suspended in 100 μl of serum-free medium were seeded onto the upper compartment of the Transwell® chambers. The Transwell® inserts were incubated for 12 h - 48 h at 37 °C in a 5 % CO_2_ humidified atmosphere, after which the cells were fixed in 10 % TCA for 15 min, rinsed and air-dried overnight. Once dry the membranes were stained with crystal violet for 20 min and the excess stain was removed by water rinsing. The non-invasive cells from the upper side of the membrane were removed by carefully sweeping with a cotton swab. Next, the membranes were removed from the inserts and placed on microscope slides and the number of invaded cells through Myogel and Matrigel® were counted. We also tested different mixtures of Myogel and Matrigel® (2 + 1, 1 + 1 and 1 + 2) in the invasion assay.

### Transwell invasion with Myogel solidified with low-melting agarose (Myogel-LMA)

We used a final concentration of 0.2 % LMA in 3.2 mg/ml final Myogel protein concentration to solidify Myogel for invasion assays. To compare the results with Matrigel® we diluted Matrigel® (BD Matrigel Matrix, BD Biosciences, Cat. Number 354234) 1 + 4 (final protein concentration 2.0 mg/ml) with the same concentration of 0.2 % agarose. Serum-free cell culture medium was used for gel dilution. Fifty μl of the Myogel-LMA or Matrigel agarose mixtures was added on the upper chamber of Transwell® nylon filter membrane insert, incubated ½ h at RT and thereafter at 37 °C in a 5 % CO_2_ humidified atmosphere until the cells were ready to be seeded on the top of the gel. HSC-3 cells were trypsinized and counted, trypsin inhibitor instead of serum-containing medium was used to inactivate trypsin. Five hundred μl of 10 % serum containing medium was added into the lower chamber of the Transwell®, and 50,000 HSC-3 cells suspended into 100 μl of medium containing 0.5 % lactalbumin instead of serum were seeded into the upper compartment of the Transwell® chamber. The cells were allowed to invade for one to three days and the invasion was quantified by staining the cells with crystal violet followed by counting the cell number. In this preliminary assay we tested different agaroses and concentrations with only one Transwell® in each condition and 0.2 % LMA was chosen for further experiments. In another set of experiments, the invasion of SCC-9, LN-1, LN-2, HSC-3, SK-Mel-25 and A2058 cells was studied in Myogel-LMA and growth factor-reduced Matrigel® (Matrigel®-GFR, BD Matrigel Matrix, BD Biosciences, Cat. Number 35430) diluted 1 + 1 with serum free medium without LMA. The invasion was quantified with Toluidine Blue -staining. Briefly, after 72 h invasion, the cells were fixed with 4 % formaldehyde for 1 h at RT, washed once with PBS, stained for 5–10 min in Toluidine blue solution (filtered 1 % Toluidine Blue + 1 % disodium tetraborate in ddH_2_0) at RT, excess dye was rinsed out with deionized water, excess gel and the cells from the upper side of the membrane were removed by using cotton swabs and when necessary, excess dye was further removed from the inside and outside of the Transwell® inserts with cotton swabs immersed in a solution of 1:1 water/ethanol. The dye was eluted by dipping the Transwell® inserts in a solution of 1 % SDS (500 μl). The 1 % SDS solution containing the eluted dye was transferred into a 96-well plate and the absorbance was measured at 650 nm.

### Hanging drop method

The gel from rat tail type I collagen (BD Biosciences) was prepared according to the manufacturer’s protocol, with a final collagen concentration of 1.7 mg/ml. The collagen/Matrigel® (BD Matrigel Matrix, BD Biosciences, Cat. Number 354234) (1.5 mg/ml, 1.5 mg/ml) and collagen/Myogel (1.5 mg/ml, 4.3 mg/ml) mixtures were prepared similarly. The hanging drop technique was used to observe the cell movement under 3D culture conditions. HSC-3 (H2B-GFP) cells were washed with PBS, trypsinized and 70,000 cells in 10 μl of DMEM/F12 medium (2 % FBS) were mixed with 50 μl of the matrix mixture. Twenty μl of the cell suspension in each matrix was dropped on the 4 compartment plate. The plate was inverted after 5 min incubation in culturing conditions and incubated for 3 h in a humidified chamber in culturing conditions. Mimosine (200 μM) was added to the medium to synchronize the cell cycle. Images were taken with a Zeiss Axio Observer.Z1 with a EC Plan-Neofluar 40x/0.75 M27 objective (Göttingen, Germany). Images consisting of 1024 x 1024 pixels were taken every 15 min with a 19.8 μm Z-stack volume (0.6 μm thickness) with a Hamamatsu Camera#2 controlled by Zeiss Zen Blue software (Zeiss) for 20 h. This assay was performed twice. The analysis of the cells in hanging drops is described in the Additional file 2: Supplementary Method.

### *In vitro* capillary tube formation assay

96-well culture plates were coated with Myogel with 2 % low melting agarose (Myogel-LMA), Matrigel®-GFR (BD Matrigel Matrix, BD Biosciences, Cat. Number 35430) or ECMatrix™ (ECMatrix – *In vitro* Angiogenesis Assay Kit, Cat. Number ECM625 – Millipore) previously thawed overnight on ice, in a total volume of 50 μl/well and allowed to solidify overnight at 37 °C. HUVEC cells were trypsinized, neutralized with DMEM/F12 with 10 % FBS, washed once with PBS and resuspended in DMEM/F12 at a density of 450,000/ml. Hundred μl of this cell suspension was added into each well and the plates were incubated at 37 °C for 12 h. Tube formation was observed under an inverted microscope (4x, Eclipse Ti-S, Nikon, Tokyo, Japan) and photos were taken and analyzed using the Motic Images Plus 2.0 software (Motic). Tubule perimeters were assessed by drawing a line around each tubule and measuring the length of the line.

### Statistical analysis

SPSS for Windows software version 21.0 (IBM) or GraphPad Prism 6 (GraphPad Software) were used for statistical analyses. To establish the statistical significance of differences between the two independent cell culture groups, a Mann–Whitney U test was used to compare the groups.

## Results

### The protein composition in different Myogel batches is reproducible and differs from that of Matrigel®

The reproducibility of different Myogel batches was first investigated in Coomassie Blue stained gradient SDS-PAGE gels. Different Myogel preparations and the mixture of various batches showed relatively similar protein band patterns, and only slight variations in the intensities of differently sized bands were visible (Additional file 1: Figures S1A and S1B). Two Myogel batches were further investigated by 2-DE (Additional file 1: Figure S1C) which confirmed with almost similar protein patterns in the silver stained 2D gels likewise the high reproducibility of these Myogel preparations.

After that the protein compositions of Myogel and Matrigel® were compared. In SDS-PAGE gels more bands were detected for the Myogel (Additional file 1: Figure S1A) and also the 2-DE separation showed a significant difference between Matrigel® and Myogel samples (Additional file 1: Figure S1C). If similar protein amounts (100 μg) were separated significantly more spots were visible for the Myogel. Increasing the amount of Matrigel® proteins to 300 μg resulted in a significantly higher number of detectable spots (Additional file 1: Figure S1C). This suggests that a few high abundant proteins, including these visible in the gel with lower protein amount, comprise a major part of the Matrigel® proteome. However, also with higher protein amounts Matrigel® and Myogel showed still distinct protein patterns. The here shown differences between Myogel and Matrigel® proteomes are possibly due to the difference between species (human *vs*. mouse) and the nature of the starting material (leiomyoma *vs*. sarcoma).

We next analyzed the protein content of Myogel and compared it with the published data of Matrigel® [32–34]. For further proteomic analyses, different Myogel batches were separated by gradient SDS-PAGE (Additional file 1: Figure S1B). Since Myogel batch number 4 differed most from the other three batches (3, 6 and 9; Additional file 1: Figure S1B), it was omitted from the mass spectrometry analysis. Two of the Myogel samples gave successful results, and altogether 765 proteins were identified (Additional file 3: Table S1). Among the Myogel proteins, 34 % (259 proteins) were the same as in Matrigel® where 1030 proteins were identified. Based on the comparison, for instance laminin, type IV collagen, heparan sulfate proteoglycans, nidogen and epidermal growth factor were found in both. Based on mass spectrometry analysis, Myogel lacked enactin, which is present in Matrigel®, but contained for example tenascin-C, collagen types XII and XIV, etc., which were lacking in Matrigel®. Based on zymography, Myogel contained both latent and active forms of MMP-2, whereas in Matrigel® latent and active forms of both MMP-2 and MMP-9 were present (Additional file 1: Figure S1D) as shown earlier [35].

### The pH of Myogel is neutral and more stable than the pH of Matrigel®

The pH of Myogel was initially between 7.0 and 7.5, and after 48 h incubation the pH remained the same. The pH of Myogel with HSC-3 cells on top dropped slightly from 7.0–7.5 to 6.5–7.0 (0 h and 48 h incubations, respectively), whereas in Matrigel® the pH at the same time dropped from a slightly alkali 8.0–8.5 to as low as 6.0–6.5 (Additional file 4: Table S2). This indicates that Myogel samples are closer than Matrigel® to neutral pH, and Myogel keeps the pH more stable than Matrigel® during cancer cells culture experiments.

### Fibroblasts adhere to Myogel, but only HSC-3 cells form colonies within Myogel- soft agar assay

**Fig. 1:**
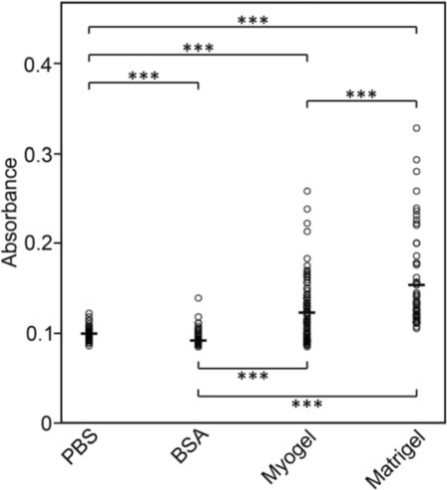
*Adhesion of HSC-3 cells to Myogel and Matrigel®.* HSC-3 cells were left to adhere to wells for 2 h. Wells coated with BSA and plain wells kept in PBS served as controls for adhesion. The adherent cells were fixed with 10 % trichloroacetic acid (TCA), stained with crystal violet and quantified using an ELISA reader at 540 nm. The number of wells was altogether 54 in PBS and BSA, 101 in Myogel and 53 in Matrigel® in three independent experiments. Horizontal lines indicate mean values, Mann–Whitney U test, *** *P* < 0.001

**Fig. 2:**
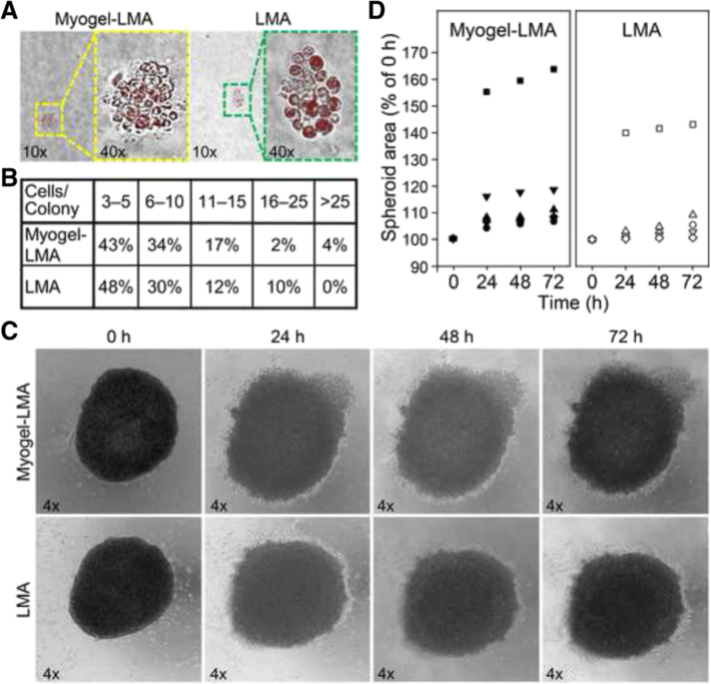
*Colony formation and hanging drop spheroid culture of HSC-3 cells within Myogel combined with LMA and plain LMA.* Colonies were photographed with 10x and 40x objectives (**a**). Cell numbers per colony were counted from the photographs (**b**). Spheroids were photographed with 4x objective (**c**). Spheroid area in ratio to 0 h spheroid area was calculated after 24 h, 48 h and 72 h incubations (**d**). Altogether 47 colonies were calculated from four Myogel-LMA and 50 colonies from four LMA wells in two independent experiments. Enlargement of altogether five spheroids in both Myogel-LMA and LMA was followed in two independent experiments

HSC-3 cells adhered significantly more to plates pre-coated with Myogel than with BSA, or to the plain plates kept in PBS. However, they adhered even more readily to Matrigel® coated plates (Fig. 1). The adhesion experiment with gingival fibroblasts (GFs) showed that after 9.5 h incubation on top of Myogel, fibroblasts were well spread and vital (not shown). Based on the TUNEL assay, from 98 to 99 % of the carcinoma associated fibroblasts (CAFs) seeded on top of various batches of Myogel were alive even after 24 h incubation (not shown). However, when CAFs were embedded within the Myogel matrix, almost all died within 21 days (not shown). In contrast, embedded HSC-3 cells stayed alive up to 28 days, divided and formed colonies within Myogel combined with LMA. The results with HSC-3 cells were relatively similar using either Myogel-LMA or a conventional soft agarose (LMA) assay (Figs. 2a and b); in Myogel-LMA the colony number was six percent less than in LMA (47 *vs.* 50). However, more colonies with the lowest cell number were present in LMA, while the highest cell number/colony was present in Myogel-LMA (Fig. 2b). According to nuclear RFP expression, 92 % of the cancer cells were alive in Myogel-LMA colonies, whereas 85 % were alive in LMA colonies. The total average area of cell colonies in Myogel-LMA was three percent less than in LMA (not shown). In HSC-3 cell hanging drop spheroid cultures the area of the spheroids embedded in Myogel-LMA grew more in 24 h incubation than in spheroids embedded in plain LMA (Fig. 2c and d). The area growing rate remained higher in Myogel-LMA even during 72 h follow-up and the spheroid area enlarged more in ratio to 0 h area in Myogel-LMA cultures than in plain LMA cultures (Fig. 2c and d). On average the increase at 72 h in ratio to 0 h was 21.5 % in Myogel-LMA and 12.2 % in plain LMA. In the most enlarged spheroids, the area increase at 72 h in Myoogel-LMA was 63.5 % while in plain LMA it was only 43.1 % (Fig. 2d). In less enlarged spheroids, the average increase at 72 h in Myogel-LMA was 11.0 % whereas in plain LMA it was 4.5 % compared to 0 h spheroids (Fig. 2d).

### Gene expression is changed when HSC-3 cells are cultured on top of Myogel compared to the same cells cultured on plastic

Gene expression assays are usually done with cells grown on tissue culture plastic. We wanted to see if Myogel coating has an effect on expressed genes in HSC-3 cells compared to cells grown on plain plastic. Approximately 1.4 % of the genes (totally 751) were differentially expressed (FC 1.5 or more) when the HSC-3 cells cultured on the top of plastic were compared to the corresponding cells grown on Myogel (raw data presented in Additional file 5: Table S3 showing 807 probes). When the genes were divided into groups according to their biological function we found that the Myogel coating affected those genes that are related to intracellular organelle and cytoskeleton organization and biogenesis. A significant change in pathway analysis was found only in the G13 signaling pathway that is related to actin polymerization and reorganization (www.genmapp.org).

### The vertical migration of HSC-3 cells is faster on Myogel than on Matrigel® coated wells

**Fig. 3:**
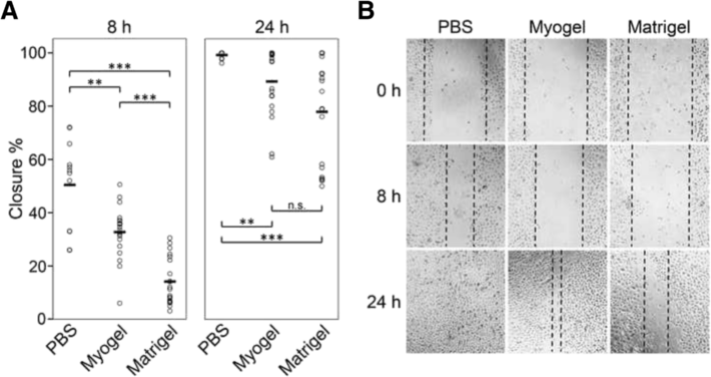
*Migration of HSC-3 cells on Myogel and Matrigel*®. HSC-3 cells migrated for 24 h in the scratch assay on Myogel and Matrigel® coated wells (**a**). Dash line in (**b**) represents the edges of the wounds. Altogether twelve wounds without coating (PBS) and twenty with Myogel or Matrigel® coating were measured in two independent experiments. Horizontal lines indicate mean values, Mann–Whitney U test, n.s. not significant, ** *P* < 0.01, *** *P* < 0.001

In the scratch assay, HSC-3 cells migrated significantly more in Myogel coated wells than in wells coated with Matrigel® (Fig. 3a and b). However, in uncoated wells their migration was faster than in coated wells (Fig. 3a and b).

### HSC-3 cells invade more efficiently through Myogel than Matrigel®

**Fig. 4:**
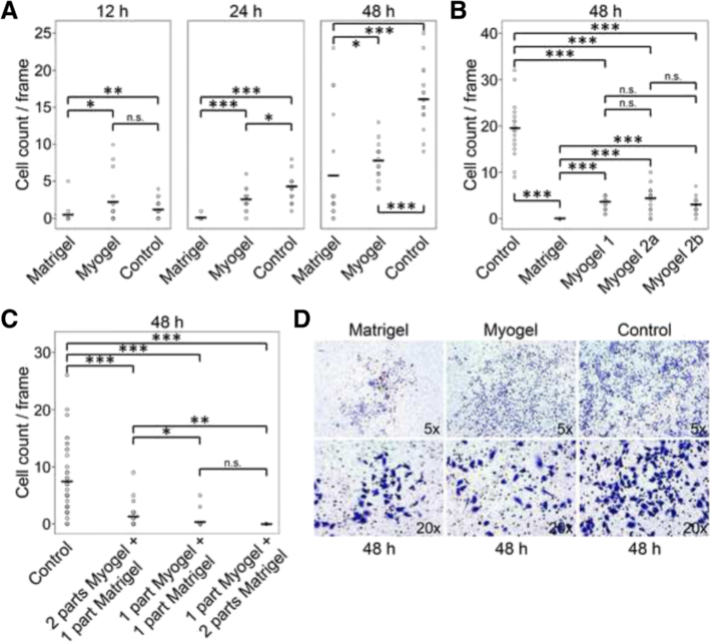
*Invasion of HSC-3 cells through Myogel and Matrigel®.* HSC-3 cells were allowed to invade for 12 – 48 h through Myogel and Matrigel® (**a**). Three different Myogel batches were compared to Matrigel® in HSC-3 cells invasion (**b**). Invasion of HSC-3 cells in different mixtures of Myogel and Matrigel® (**c**). Invasion pattern of HSC-3 cells through Myogel and Matrigel® (**d**). In **a** and **b** the number of Transwell®s was three in every group, in **c** Transwell® number was six in the control group and three in the other groups, cells from six areas of each Transwell® were calculated. Each invasion assay (**a**, **b** and **c**) was performed as an independent experiment. Controls were Transwell®s without coating. Horizontal lines indicate mean values, Mann–Whitney U test, n.s. not significant, * *P* < 0.05, ** *P* < 0.01, *** *P* < 0.001

In order to test the use of Myogel on cancer invasion Transwell® -assays, we first compared the invasion of the most aggressive oral tongue carcinoma cell line (HCS-3) using Myogel and Matrigel®. The HSC-3 cells invaded significantly more efficiently through Myogel than Matrigel® (Fig. 4a). Invasion varied slightly in different Myogel batches, but HSC-3 cells invaded in all Myogel samples more than in Matrigel® (Fig. 4b). When Myogel and Matrigel® were mixed, HSC-3 cells invaded more efficiently when the mixture contained more Myogel, and less when the portion of Matrigel® increased (Fig. 4c). The invasion pattern of HSC-3 cells was different in Myogel and Matrigel®. The cells invaded more evenly throughout the whole Transwell® membrane in Myogel coated filters, whereas in Matrigel® experiments they invaded more in clumps (Fig. 4d).

### Myogel solidified with agarose (Myogel-LMA) is a more feasible matrix for invasion assays

**Fig. 5:**
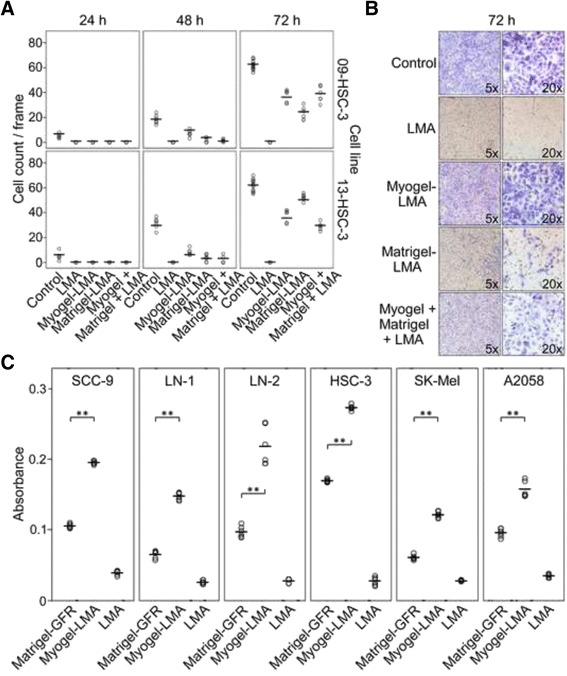
*Invasion of oral squamous cell carcinoma and melanoma cells through Myogel-LMA and Matrigel® or Matrigel®-GFR.* HSC-3 cells were allowed to invade for three days through Myogel and Matrigel® mixed with agarose, 09-HSC-3 cells have a higher passage number than 13-HSC-3 cells (**a**). Invasion pattern of HSC-3 cells through different mixtures of Myogel, Matrigel® and LMA (**b**). Oral squamous cell carcinoma and melanoma cells were allowed to invade for 72 h through Myogel-LMA and through Matrigel®-GFR (**c**). In **a** one Transwell® was analyzed for each group except for control at 72 h there were two filters (controls were Transwell®s without coating), six areas per filter were calculated, in **c** altogether six Transwell®s were analyzed in each group in two independent experiments. As only one filter was analyzed in preliminary experiment with LMA in **a**, statistical significances were not calculated in that experiment. In **c** differences were not statistically compared to plain LMA. Horizontal lines indicate mean values, Mann–Whitney U test, ** *P* < 0.01

Since Myogel batches vary slightly in their protein content, as seen in the gradient gel in Additional file 1: Figures S1A and S1B, we created a more homogenous, reliable and easier to handle matrix by adding low-melting agarose into the Myogel mixture. We noticed that HSC-3 cells did not invade through a plain agarose coated membrane, but they invaded through a Myogel-agarose (Myogel-LMA) mixture (Fig. 5a and b). Interestingly, the passage number of the HSC-3 cells seemed to affect the invasion through Myogel- and Matrigel®-mixtures; cells with higher passages invaded slightly less through Matrigel® mixtures than through Myogel-containing mixtures, whereas cells with a low passage number invaded slightly less through Myogel-LMA than through Matrigel®-LMA (Fig. 5a). The invasion pattern of HSC-3 cells through agarose-containing mixtures was similar to that observed in plain Myogel or Matrigel®; in Myogel-LMA mixtures cells invaded more evenly throughout the whole Transwell® membrane than in Matrigel®-LMA mixtures (Fig. 5b).

In another experiment, Myogel-LMA was compared with Matrigel®-GFR. In general, all the oral squamous cell carcinoma cell lines used for these invasion assays (HSC-3, LN-1, LN-2 and SCC-9) preferred Myogel-LMA to Matrigel®-GFR during the invasion (Fig. 5c). HSC-3 and LN-2 (in this order) seemed to have a higher potential to invade in both Myogel-LMA and Matrigel®-GFR, compared to the LN-1 and SSC-9 cell lines. The cells invading the Myogel-LMA seemed to keep more of the morphological characteristics observed in monolayer cultures. In contrast, the cells grouped together as they invaded the Matrigel®-GFR matrix, and lost the “fusiform-starshape” characteristic of oral carcinoma cell lines (not shown). In addition to oral carcinoma cell lines, we tested Myogel-LMA on SK-Mel and A2058 melanoma cell lines and found that also they invaded Myogel-LMA more efficiently than Matrigel®-GFR (Fig. 5c).

### HSC-3 cells move faster in Myogel-collagen matrix than in Matrigel®-collagen matrix in the 3D hanging-drop cultures

To observe the cell behavior in 3D culture in different matrices, we have established an optimized hanging drop method modified from the previously reported protocol [36]. HSC-3 cells moved in a relatively similar fashion in pure collagen, Myogel-collagen and Matrigel®-collagen matrices (Additional file 6: Movie S1, Additional file 7: Movie S2 and Additional file 8: Movie S3). However, interestingly the speed of the cells was highest in the Myogel-collagen matrix and lowest in the Matrigel®-collagen matrix (Additional file 1: Figure S2, Additional file 6: Movie S1, Additional file 7: Movie S2 and Additional file 8: Movie S3).

### Myogel efficiently induces endothelial cell tube formation

**Fig. 6:**
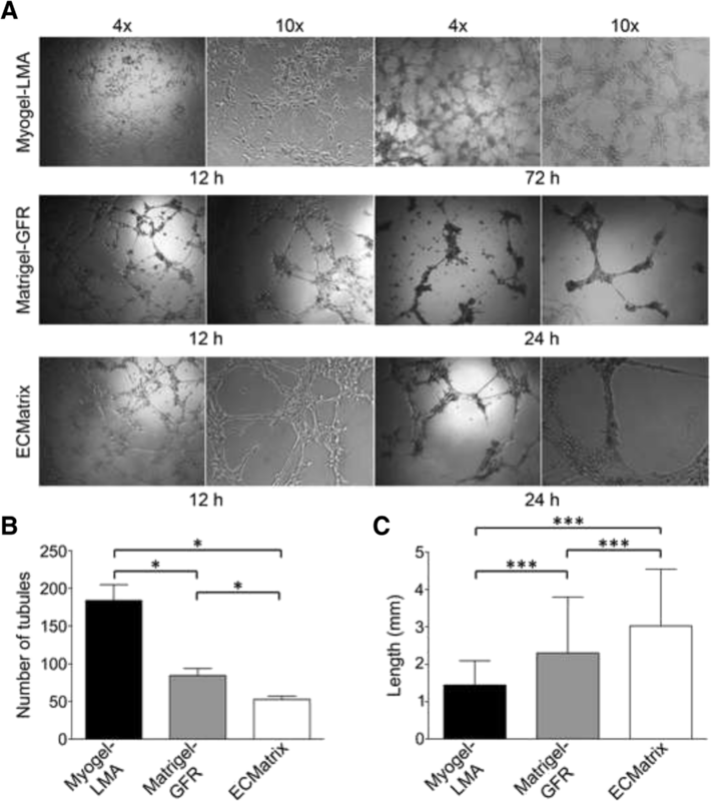
*In vitro capillary tube formation assay.* Photomicrographs showing the typical appearance of tubules formed by HUVECs in the Myogel-LMA, Matrigel®-GFR and ECMatrix™ after 12, 24 and 72 h (only for Myogel-LMA) with the original magnifications of 4x and 10x (**a**). The number of tubules formed (**b**), and the diameter of the tubules formed (**c**) in each of the three matrices is shown. Three wells were coated for each coating and three visual fields per each well were analyzed, the average value per well was used for statistical analyses. Altogether nine wells were measured in triplicate. Error bars, mean ± s.d., Mann–Whitney U test, * *P* < 0.05, *** *P* < 0.001

*In vitro* angiogenesis assays are commonly used to assess pro- or anti-angiogenic drug properties [37]. Here, tube formation could be quantified in all matrix-assays tested (Fig. 6a). With Myogel-LMA, tube formation was visible after 12 h, and even after 72 h the endothelial cells were alive (Fig. 6a), unlike in Matrigel®-GFR or ECMatrix™ (not shown), where most of the HUVEC cells were already apoptotic after 24 h. We found that the number of tubules formed was three times higher in Myogel-LMA compared to ECMatrix™ (Fig. 6b). Otherwise, measuring the diameters of the capillaries, the tubule parameters in Matrigel®-GFR, and especially in ECMatrix™ assays, were significantly higher than in tubes formed in Myogel-LMA (Fig. 6c).

## Discussion

There has been a lack of a gelatinous soluble human tumor microenvironment matrix (TMEM) for human cell line experiments *in vitro*. Today, the most widely used TMEM gelatinous material is mouse sarcoma-derived Matrigel® and other commercial and modified derivatives of mouse EHS tumors. Here, we described the preparation and use of a novel TMEM matrix, Myogel, which is derived from human uterus leiomyoma tissue. We have previously reported the use of leiomyoma tumor solid discs in several invasion studies [1–8]. Now we demonstrate that both Myogel and its combination with low melting agarose, Myogel-LMA, provide practical *in vitro* gelatinous TMEM for testing cancer cells invasive, adhesive or migratory properties, that is similar to Matrigel®, Matrigel®-GFR or ECMatrix™. The benefit of Myogel over various EHS tumor derived products is that the protein composition of human Myogel differs significantly from the mouse sarcoma matrix as seen by gradient SDS-PAGE gels, 2-DE, zymography and proteomics analyses. Moreover, the pH is more stable in Myogel than in Matrigel® during cell culture. In addition, human uterus myomas, which are by-products of surgical operations, are an ethically superior starting material compared to EHS tumors in mice, which are grown purely for preparing TMEM for research purposes. Myogel and in particular Myogel-LMA, is easy to prepare and handle. Finally, and most importantly, in all assays we have tested thus far, the results have been reproducible and similar or even superior to Matrigel®. Namely, the various cancer cells analyzed invaded through Myogel or Myogel-LMA generally more efficiently than through Matrigel® or Matrigel®-GFR. Embedded HSC-3 cells tended to form larger colonies and spheroids in soft agar colony formation -assay and in hanging drop spheroid cultures, respectively, within Myogel-LMA than within plain LMA. HUVEC cells in Myogel-LMA formed more and smaller tubules which lasted longer than in Matrigel-GFR® or in ECMatrix. In addition, we demonstrated in hanging drop assays that carcinoma cells were able to move more rapidly in a Myogel-collagen matrix than in collagen, or in Matrigel®-collagen combinations.

Uterine leiomyomas are benign smooth muscle tumors affecting the health of millions of females. The tumors have at least four molecular subclasses and contain chromothripsis, which has previously been associated with aggressive cancer [38, 39]. As a patient derived material, there is heterogeneity between myomas. The operating surgeon omits peculiar ones. The prepared batch is always tested in an invasion assay with our reference HSC-3 cells. To ensure the reproducibility between the Myogel batches, we combine several myomas for each batch. As a solid tumor mass, easy to obtain and handle, myoma tissue is an ideal matrix material for several experimental cancer study applications *in vitro*. At present, all the other human ECM matrices to investigate the properties of cancer cell lines are derived either from normal human tissues or cultured cells. Since the composition of Myogel simulates well the TMEM of human solid cancers, it clearly offers improved characteristics also over mouse tumor matrix derivative Matrigel® which is mouse EHS tumor-derived and has batch-to-batch variation. Myogel, used for coating cell culture wells, offered a matrix on which normal and transformed cells attached more efficiently than on BSA or plain plastic wells with PBS. It was beneficial also in vertical scratch assays. In addition, it was interesting to note that a Myogel coating affected the expression of 750 genes in HSC-3 carcinoma cells compared to plain plastic, the method commonly used in RNA-microarray analyses. These genes were related to actin filament reorganization and contributed to cell motility providing some insight into the mechanisms behind the more intense migration of cancer cells on, through or in Myogel containing matrix in the experiments measuring cell movement. The factors in this G13 signaling pathway, such as Wiskott-Aldrich syndrome-like (WASP), play important roles in cell response to extracellular stimuli by actin filament reorganization, and they are also known to contribute crucially to cancer cell motility [40]. Therefore, our results suggest that Myogel provides a more complex, natural human TMEM substrate for oncogenic assays than cell culture on plain plastic or even wells coated with individual molecules, such as collagen, fibronectin or vitronectin.

The mesenchymal fibroblasts (GFs and CAFs) remained viable when seeded on Myogel, but when embedded in a thick layer of Myogel-LMA they became apoptotic. However, carcinoma cells formed vital colonies even for up to 28 days. This indicates that Myogel, as a homogenous protein solution, cannot provide the collagenous fiber structures required for non-transformed cells, like fibroblasts, to survive. Instead, Myogel-LMA is ideal for soft agar -type assay commonly used to select normal from transformed cells [41]. Adding Myogel in agarose provides the cells a more natural TMEM, which most probably affects their growing potential. Here we saw a slight tendency of the carcinoma cells to form more large colonies in Myogel-LMA than in the plain LMA matrix. The hanging drop spheroids embedded in Myogel-LMA also seemed to grow more than the ones embedded in plain LMA.

The Transwell® invasion assay, conducted classically by applying either Matrigel® or Matrigel®-GFR to Transwell® inserts [11], worked well with either Myogel or Myogel-LMA. Interestingly, we were able to show that carcinoma cells invaded in Myogel even more efficiently than in Matrigel®. Cancer cells also invaded through Myogel-LMA mixtures more evenly throughout the whole membrane, whereas in Matrigel®-mixtures the cells tended to invade in clumps. Myogel seems to offer an excellent, natural human TMEM, which helps to simulate the proper, natural interaction between TMEM and cancer cells. In our preliminary experiments we have also succeeded in using Myogel-LMA, instead of Matrigel®, in several chemotherapeutic and irradiation *in vitro* experiments (data not shown). Based on these experiments, we believe that Myogel-LMA is able to highly improve the quality of various preclinical human cancer studies.

For the study of tumor cell behavior, it is important to observe the cells under biological conditions since the cellular motility, form and growth in monolayer culture differ from that observed in 3D culture [42]. The hanging drop is a method of 3D cell culture, which mimics the environment of the cells within a tissue [43, 44]. This method is preferred compared to the other 3D cell culture settings since it allows the cells to form spheroids, in which cells are cultured in close contact to each other, making a small aggregated component. In hanging drop assay, spheroid is a form of an aggregated cluster of cells cultured in 3D physiological condition. The spheroids developed using the hanging drop method are widely used as a model for the study of cell-cell interactions [45], *in vivo* tumor tissue organization and microenvironment [46], and the melanoma reconstruction model [47]. In this study, we have optimized the method for the observation of single tumor cell motility in different matrices. When the speed of cell movement was compared between the matrices, the difference was clear: HSC-3 cells moved most rapidly in Myogel-collagen and their movement was the slowest in Matrigel®-collagen.

We demonstrated that Myogel-LMA could also be applied to angiogenesis studies, where it represented a reliable way to produce and analyze data regarding vascular tube formation. In Myogel-LMA the number of tubes formed was higher but they were smaller in circumference compared to the other matrices. The vessel formation assay using Myogel-LMA could thus be suitable for pro- or anti-angiogenic drug assays where the number of tubes is the most critical factor to be evaluated. The differences between Matrigel®-GFR and ECMatrix™ assays could be explained by the fact that the levels of stimulatory cytokines and growth factors have been markedly reduced in the growth factor reduced Matrigel® to avoid problems associated with the over-stimulation of endothelial cells. However, according to the manufacturer, ECMatrix™ is derived from EHS tumors and is similar to “normal” Matrigel® and contains *e.g*. laminin, collagen type IV, heparan sulfate proteoglycans, entactin and nidogen. It also includes various growth factors (TGF-β, EFG) and proteolytic enzymes (plasminogen, tPA, MMPs) that should optimize it for maximal tube-formation. In all matrices, cellular network structures were fully developed by 12 h, with the first signs apparent after 5–6 h. However, using Myogel-LMA, tube formation continued for as long as for 72 h, whereas at that time the endothelial cells in Matrigel®-GFR and ECMatrix™ were already in apoptosis.

## Conclusions

In conclusion, Myogel, especially easy-to-use Myogel-LMA, is well suited for *in vitro* cancer and angiogenesis studies. In some experiments Myogel is even superior to the Matrigel®, or rat tail type I collagen -based matrices. Using easily obtained, human uterus leiomyoma tissue to produce Myogel, production costs are relatively low. In addition, the use of myoma tissue is ethically superior to the sacrifice thousands of mice with EHS tumors required for the production of Matrigel®. Myogel together with the myoma disc organotypic model, could offer a natural human TMEM based kit to study various cancer cell lines, chemotherapy or irradiation effects *in vitro.* In theory, these materials may well be usable also in the future for personalized medicine. Myogel-LMA could facilitate the culture of cells from a fresh tumor tissue biopsy or a cell suspension and the effects of drugs or chemoradiation therapies tested to optimize the treatment for the patient.

## Availability of Data

The microarray data reported in this paper have been deposited in the Gene Expression Omnibus (GEO) database, www.ncbi.nlm.nih.gov/geo (accession no. GSE68722).

## Additional files

Additional file 1:
**Figure S1.** *Gradient SDS-PAGE, 2-DE and zymography from Myogel and Matrigel®.* Coomassie Blue stained gradient SDS-PAGE gel from four Myogel batches (12, 15–17) and their mixture and four commercial Matrigel® samples (20 μg protein, A). Coomassie Blue stained gradient SDS-PAGE gel from four Myogel samples (3, 4, 6 and 9) for Myogel proteomics by mass spectrometry (10 μg and 20 μg protein, B). Silver 2D gels of Myogel (batches 16 and 17, 100 μg protein) and Matrigel® (100 and 300 μg protein) samples (C). Gelatinolytic activities in Myogel and Matrigel® detected under long wave UV light by zymography. The empty lanes between the standard and Myogel-sample, Myogel- and Matrigel®-samples and Matrigel®-sample and control MMP-2 were deleted from the picture and these lanes are divided by white lines, control MMP-2 and −9 were initially in adjacent wells. (D). Gradient gel in A and zymography were run twice, gradient gel for proteomics and 2-DE gels once. **Figure S2.** *Average* *speed of HSC-3 cell movement in hanging drops*. HSC-3 cells were cultured in hanging drops in pure collagen, Myogel-collagen and Matrigel®-collagen matrices. Pictures were taken every 15 min for 20 h with a 40x objective. The hanging drop -assay was performed twice, but the analysis was performed from one experiment. (PDF 660 kb) Additional file 2: Supplementary Method. The analysis of the cells in hanging drops. (PDF 591 kb) Additional file 3: Table S1. Mass spectrometry results on myogel. (PDF 640 kb) Additional file 4: Table S2. The pH-measurements of Myogel and Matrigel®. (PDF 111 kb) Additional file 5: Table S3. Microarray results showing genes with FC 1.5 or more when the HSC-3 cells cultured on the top of plastic were compared to the corresponding cells grown on Myogel. (PDF 1329 kb) Additional file 6: Movie S1. Nuclear stain, collagen.avi. Video showing cell movements in the hanging drop culture in pure collagen matrix tracked by nuclear stain. (AVI 4793 kb) Additional file 7: Movie S2. Nuclear stain, Myogel-collagen.avi. Video showing cell movements in the hanging drop culture in Myogel-collagen matrix tracked by nuclear stain. (AVI 3832 kb) Additional file 8: Movie S3. Nuclear stain, Matrigel-collagen.avi. Video showing cell movements in the hanging drop culture in Matrigel®-collagen matrix tracked by nuclear stain. (AVI 4273 kb)

## Abbreviations

2-DE: Two-dimensional gel electrophoresis
ATCC: American type culture collection
BSA: Bovine serum albumin
CAF: Carcinoma associated fibroblast
ECM: Extracellular matrix
EGF: Epidermal growth factor
EHS: Engelbreth-Holm-Swarm
FBS: Fetal bovine serum
FC: Fold change
FDR: False discovery rate
FGF: Fibroblast growth factor
GEO: Gene expression omnibus
GF: Gingival fibroblast
GFP: Green fluorescent protein
GO: Gene ontology
H2B: Histone-2B
HUVEC: Human umbilical vein endothelial cell
IEF: Isoelectric focusing
LC: Liquid chromatography
LMA: Low melting agarose
MMP: Matrix metalloproteinase
MS: Mass spectrometry
Myogel-LMA: Myogel mixed with low-melting agarose
PBS: Phosphate-buffered saline
PDGF: Platelet-derived growth factor
TCA: Trichloroacetic acid
TGF-α: Transforming growth factor alpha
TGF-ß: Transforming growth factor beta
TME: Tumor microenvironment
TMEM: Tumor microenvironment matrix
tPA: Tissue plasminogen activator
TUNEL: Terminal deoxynucleotidyl transferase dUTP nick end labeling
WASP: Wiskott-Aldrich syndrome-like
