# Supplementary material for: A novel human leiomyoma tissue derived matrix for cell culture studies
Source: BMC Cancer. 2015 Dec 16;15:981. doi: 10.1186/s12885-015-1944-z (PMC4682271; doi:10.1186/s12885-015-1944-z)
Supplement: Additional file 1: — Figure S1. Gradient SDS-PAGE, 2-DE and zymography from Myogel and Matrigel®. Coomassie Blue stained gradient SDS-PAGE gel from four Myogel batches (12, 15–17) and their mixture and four commercial Matrigel® samples (20 μg protein, A). Coomassie Blue stained gradient SDS-PAGE gel from four Myogel samples (3, 4, 6 and 9) for Myogel proteomics by mass spectrometry (10 μg and 20 μg protein, B). Silver 2D gels of Myogel (batches 16 and 17, 100 μg protein) and Matrigel® (100 and 300 μg protein) samples (C). Gelatinolytic activities in Myogel and Matrigel® detected under long wave UV light by zymography. The empty lanes between the standard and Myogel-sample, Myogel- and Matrigel®-samples and Matrigel®-sample and control MMP-2 were deleted from the picture and these lanes are divided by white lines, control MMP-2 and −9 were initially in adjacent wells. (D). Gradient gel in A and zymography were run twice, gradient gel for proteomics and 2-DE gels once. Figure S2. Average speed of HSC-3 cell movement in hanging drops. HSC-3 cells were cultured in hanging drops in pure collagen, Myogel-collagen and Matrigel®-collagen matrices. Pictures were taken every 15 min for 20 h with a 40x objective. The hanging drop -assay was performed twice, but the analysis was performed from one experiment. (PDF 660 kb) [file 12885_2015_1944_MOESM1_ESM.pdf]

Supplementary Figures

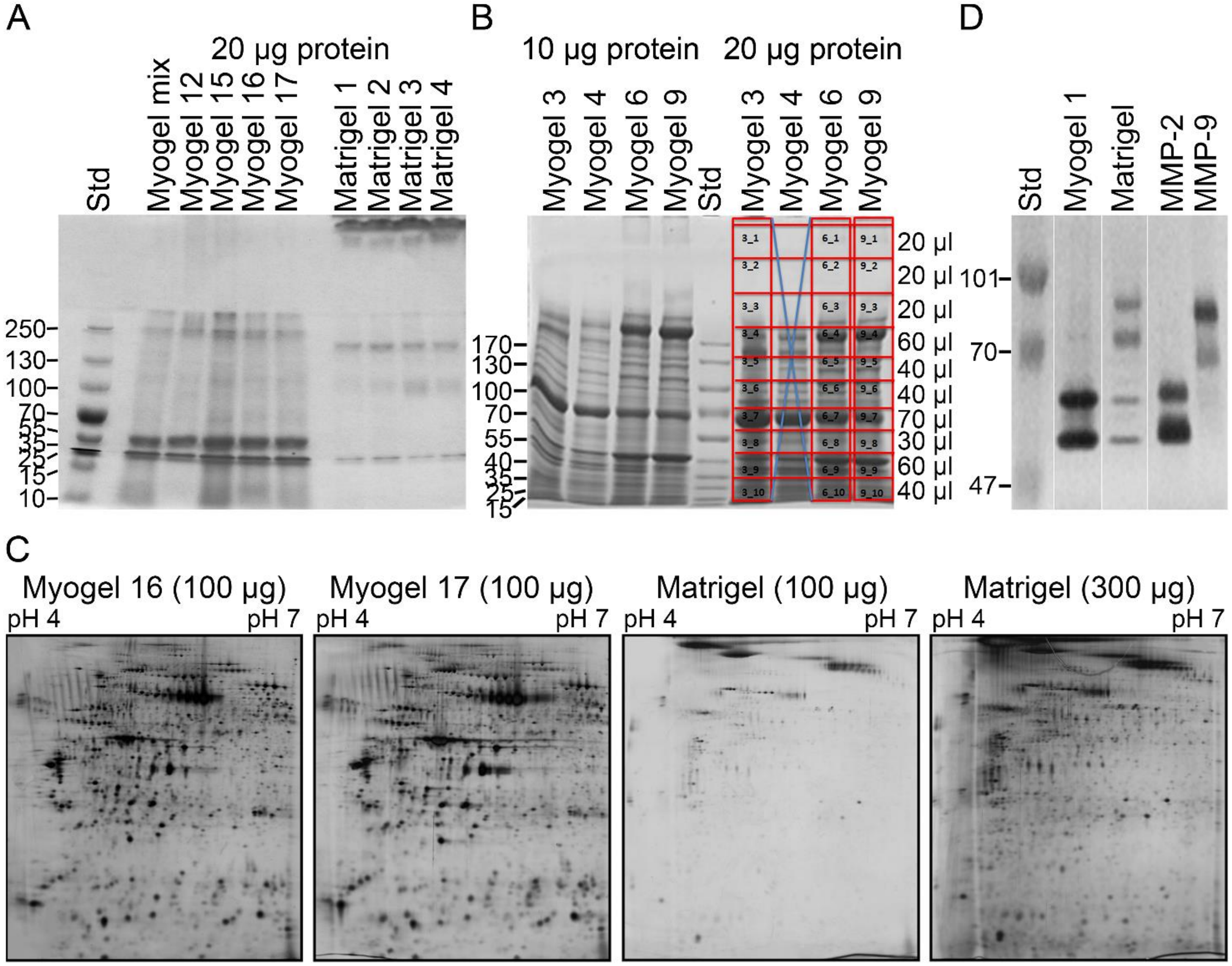

Figure S1. *Gradient SDS-PAGE, 2-DE and zymography from Myogel and Matrigel®.* Coomassie Blue stained gradient SDS-PAGE gel from four Myogel batches (12, 15-17) and their mixture and four commercial Matrigel® samples (20 µg protein, **A**). Coomassie Blue stained gradient SDS-PAGE gel from four Myogel samples (3, 4, 6 and 9) for Myogel proteomics by mass spectrometry (10 µg and 20 µg protein, **B**). Silver 2D gels of Myogel (batches 16 and 17, 100 µg protein) and Matrigel® (100 and 300 µg protein) samples (**C**). Gelatinolytic activities in Myogel and Matrigel® detected under long wave UV light by zymography. The empty lanes between the standard and Myogel-sample, Myogel- and Matrigel®-samples and Matrigel®-sample and control MMP-2 were deleted from the picture and these lanes are divided by white lines, control MMP-2 and -9 were initially in adjacent wells. (**D**). Gradient gel in **A** and zymography were run twice, gradient gel for proteomics and 2-DE gels once.

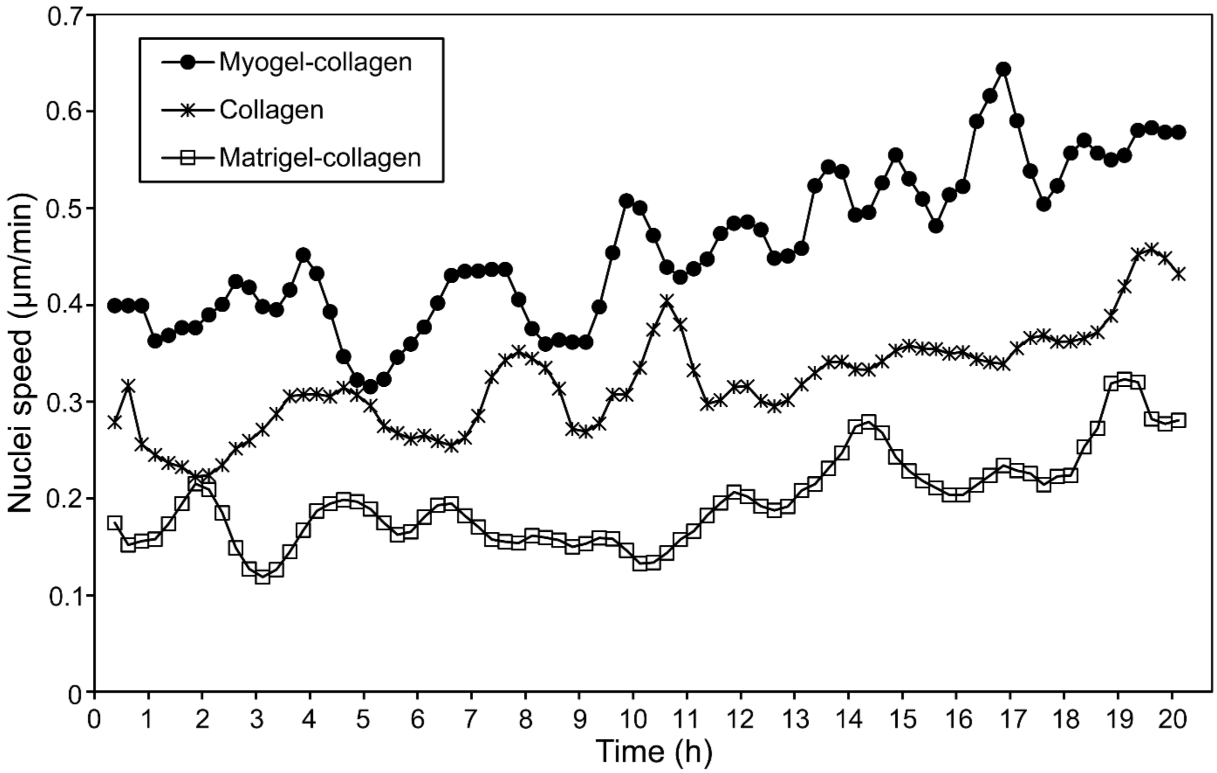

Figure S2. *Average speed of HSC-3 cell movement in hanging drops.* HSC-3 cells were cultured in hanging drops in pure collagen, Myogel-collagen and Matrigel®-collagen matrices. Pictures were taken every 15 min for 20 h with a 40x objective. The hanging drop -assay was performed twice, but the analysis was performed from one experiment.
