# Supplementary material for: A novel human leiomyoma tissue derived matrix for cell culture studies
Source: BMC Cancer. 2015 Dec 16;15:981. doi: 10.1186/s12885-015-1944-z (PMC4682271; doi:10.1186/s12885-015-1944-z)
Supplement: Additional file 2: Supplementary Method. — The analysis of the cells in hanging drops. (PDF 591 kb) [file 12885_2015_1944_MOESM2_ESM.pdf]

Supplementary Method. The analysis of the cells in hanging drops

1. Cell Analysis Method

1.1. Segmentation

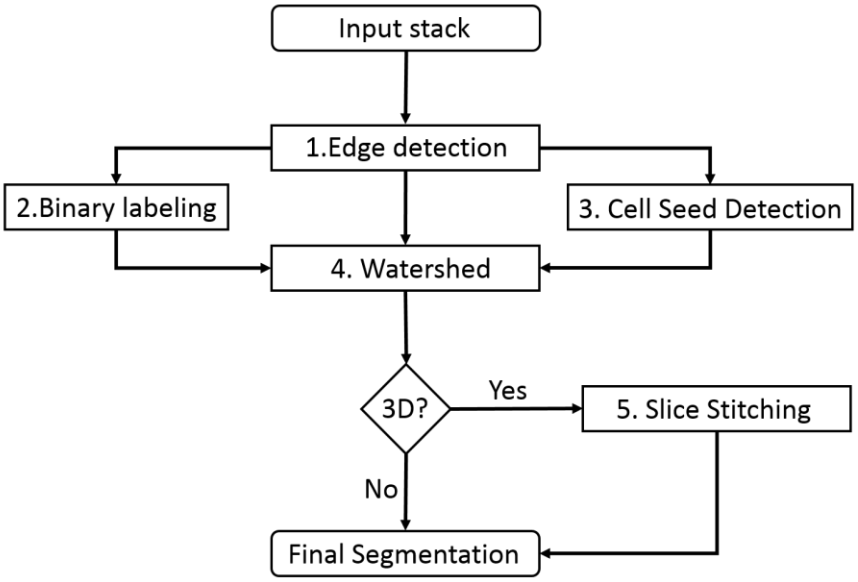

Fig. 1: Flow chart showing the overview of our segmentation method.

The overview of our segmentation method [1] is shown in Fig. 1 and here we describe it very briefly. The method consists of five stages for 3D stacks, stages 1-4 process one slice at a time. In first stage 'Edge Detection', image noise is reduced using median filtering. Then edge detector [2] which uses structured random forest and a large set of features from multi-scale local windows, is used to compute the edge probabilities at each pixel. In second stage, we separate background and cell pixels by creating a grid graph in which terminal edge weights are set using the pixel intensity and threshold computed from its local neighborhood. Edge costs between neighboring pixels are set using the directional edge probabilities computed in first stage. Graph

cut is used to solve this graph and find the minimum energy labeling. In the third stage 'Cell Seed Detection', we create a second graph in which the terminal edge costs also take into account the edge probability at that pixel. Using the edge probabilities allows us to create a barrier between cells which are in contact with each other. We solve this graph again using graph cuts, which produces a unique connected component for each cell. We label these components and use them as cell seeds. In stage four we use marker controlled watershed to expand the cell seeds. Finally in stage five, we combine the segmented objects in each slice to obtain the 3D segmented stack.

1.2. Tracking

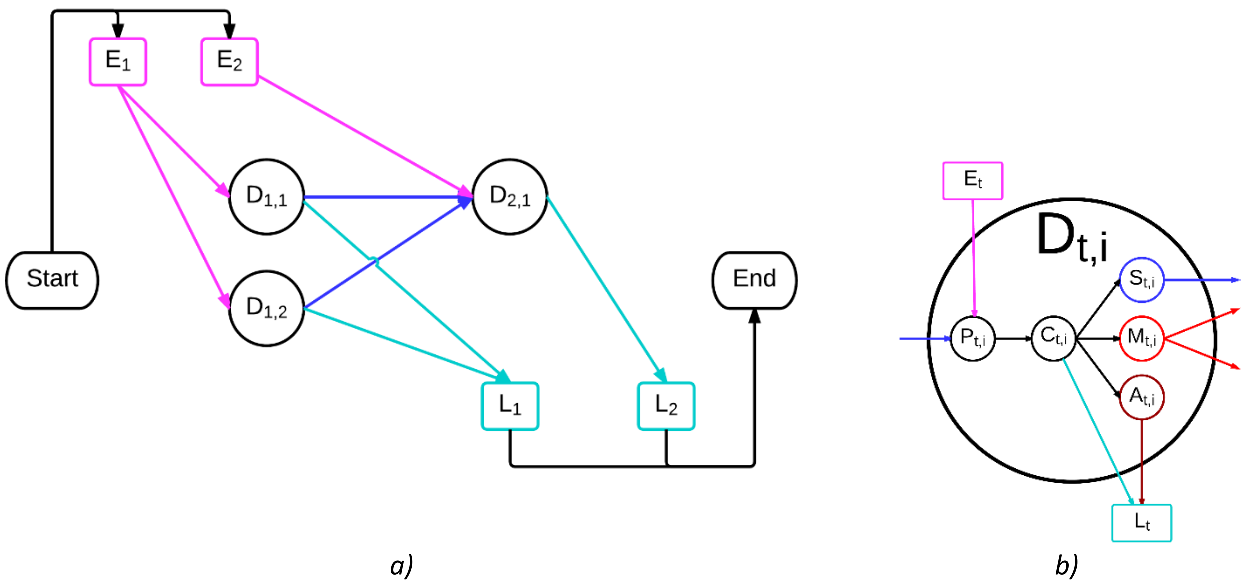

Fig. 2: a) Basic structure of DAG used for tracking. b) Structure of a detection node (super-node).

We use an iterative tracking method similar to [3] which iteratively finds cell tracks by searching for the minimum cost path in a directed acyclic graph (DAG), which encodes the transition probabilities for each detection. This type of approach has a limitation that transition probabilities cannot depend on information older than one previous frame and have to satisfy the Markov assumption. In case of cell sequences most of the transition probabilities satisfy this assumption to a large degree so it does not hamper the performance of the system significantly.

Fig. 2 shows the basic structure of the DAG that we use and the detailed structure of detection super-nodes. The DAG consists of a '*Start*' and '*End*' node, which are the first and last nodes respectively for all tracks. The '*Start*' node is connected to entry nodes, E_t, which allow a track to begin from frame '*t*'. Similarly the '*End*' node is connected to leave nodes, L_t, which allow tracks to exit from that frame. The edges of entry and leave nodes to detections are set based on the distance of their centroids from the boundary of region being imaged. The cells are assumed to be following a Brownian motion model and edge costs connecting nodes in adjacent frames are set using the distance between their centroids.

Segmentation methods are prone to failures so it is necessary for a tracking method to be able to cope and correct some segmentation errors. We connect detections in one frame with few future frames using skip edges to handle (prevent splitting of tracks) situations in which segmentation fails to detect a cell in some frames. One common segmentation error in dense sequences is under-segmentation error (multiple cells are segmented as one cell), this tracking

approach can handle this problem by allowing multiple cell tracks to pass through a single detection. We encode the probability of a detection containing multiple cells using the edge between nodes $P_{t,i}$ and $C_{t,i}$. We have used a simple Gaussian model of cell size to compute this probability due to lack of any training data. Each super-node, $D_{t,i}$ contains some additional nodes that can be used to encode cell event (mitosis, apoptosis) probabilities. We have not utilized the mitosis and apoptosis nodes in these experiments since we do not have any training data and it is not trivial to model the probabilities associated with these events.

We use Viterbi algorithm to find the lowest cost path. Then we modify the graph by updating the number of cells passing through each detection in that path and updating the cost of edge between nodes $P_{t,i}$ and $C_{t,i}$. We also create swap nodes for each track at each frame that it is active in so that future tracks can split and swap their second part with pre-existing tracks if the total cost is reduced by this swapping.

Once the cells and nuclei have been segmented and tracked, then their mean speed, size and roundness (eccentricity) are computed and the changes in these variables for each sequence are analyzed.

2. Results

The nuclei count was higher and varied more in all matrices than the cytosomal cell count; the count was highest in pure collagen in both (not shown). The nuclei size was about the same and rather even in all the matrices while the cell size varied more, both between the matrices and during the recording in each matrix. Both the nuclei and the cell sizes were largest in the Matrigel®-collagen matrix (not shown). The average eccentricity/roundness measured by nuclear stain was about the same and rather even in all the matrices while it was highest in pure collagen and varied more when measured by cytosomal stain (not shown). The speed of cell migration was rather similar if measured by nuclear or cytosomal stain in each matrix.

3. Discussion

We observed both the nuclei and cell cytoplasm according to different stains used to track them. There were some differences in count as not all the cells have both signals equally visible. Nuclei were readily detected (they have relatively small variation in their intensity). The cytoplasmic variation in intensity was very large and it was more difficult to find a good balance between detecting actual cells without resulting in the detection of false cells. The nuclei size variation was small as changes are mainly due to cell division whereas cell size varies more due to changes in cell shape during the cell movement. We did not observe differences in nuclei eccentricity/roundness (as expected), nor did we distinguish any clear changes in cell shape (circular vs. elongated) during movement of carcinoma cells in these three matrices used.

However, this method is useful for the future analyses. The speed of cell movement was similar in one matrix measured by nuclear and cytosomal stains.

4. References

1. Akram S, Kannala J, Kaakinen M, Eklund L, Heikkilä J. Segmentation of cells from spinning disk confocal images using a multi-stage approach. In Asian Conference on Computer Vision (ACCV, Singapore) 2014.

2. Dollár P, Zitnick CL. Structured forests for fast edge detection. In International Conference on Computer Vision (ICCV). 2013.

3. Magnusson K, Joakim J. A batch algorithm using iterative application of the viterbi algorithm to track cells and construct cell lineages. In Biomedical Imaging (ISBI) 9th IEEE International Symposium on (Barcelona). 2012;doi:10.1109/ISBI.2012.6235564.
