# Supplementary material for: A novel human leiomyoma tissue derived matrix for cell culture studies
Source: BMC Cancer. 2015 Dec 16;15:981. doi: 10.1186/s12885-015-1944-z (PMC4682271; doi:10.1186/s12885-015-1944-z)
Supplement: Additional file 3: Table S1. — Mass spectrometry results on myogel. (PDF 640 kb) [file 12885_2015_1944_MOESM3_ESM.pdf]

Table S1. Mass spectrometry results on myogel

	Identified Proteins (765)	UniProt Accession Number
1	Myosin-11 OS=Homo sapiens GN=MYH11 PE=1 SV=3	sp|P35749|MYH11_HUMAN
2	Isoform 2 of Filamin-A OS=Homo sapiens GN=FLNA	sp|P21333-2|FLNA_HUMAN
3	Collagen alpha-1(XII) chain OS=Homo sapiens GN=COL12A1 PE=1 SV=2	sp|Q99715|COCA1_HUMAN (+1)
4	Serum albumin OS=Homo sapiens GN=ALB PE=1 SV=2	sp|P02768|ALBU_HUMAN
5	Talin-1 OS=Homo sapiens GN=TLN1 PE=1 SV=3	sp|Q9Y490|TLN1_HUMAN
6	Actin, aortic smooth muscle OS=Homo sapiens GN=ACTA2 PE=1 SV=1	sp|P62736|ACTA_HUMAN (+1)
7	Collagen alpha-1(XIV) chain OS=Homo sapiens GN=COL14A1 PE=1 SV=3	sp|Q05707|COEA1_HUMAN
8	Isoform 2 of Collagen alpha-3(VI) chain OS=Homo sapiens GN=COL6A3	sp|P12111-2|CO6A3_HUMAN (+1)
9	Isoform 1 of Vinculin OS=Homo sapiens GN=VCL	sp|P18206-2|VINC_HUMAN (+1)
10	Myosin-9 OS=Homo sapiens GN=MYH9 PE=1 SV=4	sp|P35579|MYH9_HUMAN
11	Actin, cytoplasmic 1 OS=Homo sapiens GN=ACTB PE=1 SV=1	sp|P60709|ACTB_HUMAN (+1)
12	Isoform 2 of Alpha-actinin-1 OS=Homo sapiens GN=ACTN1	sp|P12814-2|ACTN1_HUMAN (+1)
13	Isoform 2 of Filamin-C OS=Homo sapiens GN=FLNC	sp|Q14315-2|FLNC_HUMAN (+1)
14	Tenascin OS=Homo sapiens GN=TNC PE=1 SV=3	sp|P24821|TENA_HUMAN
15	Desmin OS=Homo sapiens GN=DES PE=1 SV=3	sp|P17661|DESM_HUMAN
16	Isoform 3 of Plectin OS=Homo sapiens GN=PLEC	sp|Q15149-3|PLEC_HUMAN
17	Alpha-1-antitrypsin OS=Homo sapiens GN=SERPINA1 PE=1 SV=3	sp|P01009|A1AT_HUMAN
18	Transgelin OS=Homo sapiens GN=TAGLN PE=1 SV=4	sp|Q01995|TAGL_HUMAN
19	Prelamin-A/C OS=Homo sapiens GN=LMNA PE=1 SV=1	sp|P02545|LMNA_HUMAN
20	Caldesmon OS=Homo sapiens GN=CALD1 PE=1 SV=3	sp|Q05682|CALD1_HUMAN
21	Gelsolin OS=Homo sapiens GN=GSN PE=1 SV=1	sp|P06396|GELS_HUMAN
22	Collagen alpha-2(I) chain OS=Homo sapiens GN=COL1A2 PE=1 SV=7	sp|P08123|CO1A2_HUMAN
23	Collagen alpha-1(VI) chain OS=Homo sapiens GN=COL6A1 PE=1 SV=3	sp|P12109|CO6A1_HUMAN
24	Serotransferrin OS=Homo sapiens GN=TF PE=1 SV=3	sp|P02787|TRFE_HUMAN
25	Vimentin OS=Homo sapiens GN=VIM PE=1 SV=4	sp|P08670|VIME_HUMAN
26	Basement membrane-specific heparan sulfate proteoglycan core protein OS=Homo sapiens GN=HSPG2 PE=1 SV=4	sp|P98160|PGBM_HUMAN
27	Complement C3 OS=Homo sapiens GN=C3 PE=1 SV=2	sp|P01024|CO3_HUMAN
28	Tropomyosin 1 (Alpha), isoform CRA_f OS=Homo sapiens GN=TPM1 PE=2 SV=1	tr|Q6ZN40|Q6ZN40_HUMAN
29	Isoform 3 of Tenascin-X OS=Homo sapiens GN=TNXB	sp|P22105-3|TENX_HUMAN (+2)

30	Collagen alpha-2(VI) chain OS=Homo sapiens GN=COL6A2 PE=1 SV=4	sp|P12110|CO6A2_HUMAN
31	Keratin, type II cytoskeletal 1 OS=Homo sapiens GN=KRT1 PE=1 SV=6	sp|P04264|K2C1_HUMAN
32	Annexin OS=Homo sapiens GN=ANXA6 PE=2 SV=3	tr|A6NN80|A6NN80_HUMAN
33	Alpha-actinin-4 OS=Homo sapiens GN=ACTN4 PE=1 SV=2	sp|O43707|ACTN4_HUMAN
34	Isoform 2 of Tropomyosin beta chain OS=Homo sapiens GN=TPM2	sp|P07951-2|TPM2_HUMAN
35	Heat shock 70 kDa protein 1A/1B OS=Homo sapiens GN=HSPA1A PE=1 SV=5	sp|P08107|HSP71_HUMAN
36	Periostin OS=Homo sapiens GN=POSTN PE=2 SV=1	tr|B1ALD8|B1ALD8_HUMAN
37	Cytoplasmic dynein 1 heavy chain 1 OS=Homo sapiens GN=DYNC1H1 PE=1 SV=5	sp|Q14204|DYHC1_HUMAN
38	Lumican OS=Homo sapiens GN=LUM PE=1 SV=2	sp|P51884|LUM_HUMAN
39	Moesin OS=Homo sapiens GN=MSN PE=1 SV=3	sp|P26038|MOES_HUMAN
40	Isoform 2 of Myosin light chain kinase, smooth muscle OS=Homo sapiens GN=MYLK	sp|Q15746-2|MYLK_HUMAN (+3)
41	Galectin-1 OS=Homo sapiens GN=LGALS1 PE=1 SV=2	sp|P09382|LEG1_HUMAN
42	Collagen alpha-1(I) chain OS=Homo sapiens GN=COL1A1 PE=1 SV=5	sp|P02452|CO1A1_HUMAN
43	Laminin subunit gamma-1 OS=Homo sapiens GN=LAMC1 PE=1 SV=3	sp|P11047|LAMC1_HUMAN
44	Alpha-2-macroglobulin OS=Homo sapiens GN=A2M PE=1 SV=3	sp|P01023|A2MG_HUMAN
45	Ras GTPase-activating-like protein IQGAP1 OS=Homo sapiens GN=IQGAP1 PE=1 SV=1	sp|P46940|IQGA1_HUMAN
46	Pyruvate kinase PKM OS=Homo sapiens GN=PKM PE=1 SV=4	sp|P14618|KPYM_HUMAN
47	Phosphoglucomutase-like protein 5 OS=Homo sapiens GN=PGM5 PE=1 SV=2	sp|Q15124|PGM5_HUMAN
48	Laminin subunit beta-1 OS=Homo sapiens GN=LAMB1 PE=1 SV=2	sp|P07942|LAMB1_HUMAN (+1)
49	Tubulin beta chain OS=Homo sapiens GN=TUBB PE=1 SV=2	sp|P07437|TBB5_HUMAN (+1)
50	Isoform 17 of Fibronectin OS=Homo sapiens GN=FN1	sp|P02751-17|FINC_HUMAN (+5)
51	Alpha-enolase OS=Homo sapiens GN=ENO1 PE=1 SV=2	sp|P06733|ENOA_HUMAN
52	Isoform 9 of Sorbin and SH3 domain-containing protein 1 OS=Homo sapiens GN=SORBS1	sp|Q9BX66-9|SRBS1_HUMAN
53	ATP synthase subunit beta, mitochondrial OS=Homo sapiens GN=ATP5B PE=1 SV=3	sp|P06576|ATPB_HUMAN
54	Isoform LCRMP-4 of Dihydropyrimidinase-related protein 3 OS=Homo sapiens GN=DPYSL3	sp|Q14195-2|DPYL3_HUMAN
55	Keratin, type I cytoskeletal 10 OS=Homo sapiens GN=KRT10 PE=1 SV=6	sp|P13645|K1C10_HUMAN
56	Isoform 2 of Clathrin heavy chain 1 OS=Homo sapiens GN=CLTC	sp|Q00610-2|CLH1_HUMAN (+1)
57	Tubulin alpha-1C chain OS=Homo sapiens GN=TUBA1C PE=1 SV=1	sp|Q9BQE3|TBA1C_HUMAN (+1)
58	Keratin, type I cytoskeletal 9 OS=Homo sapiens GN=KRT9 PE=1 SV=3	sp|P35527|K1C9_HUMAN
59	Histone H2A type 1-B/E OS=Homo sapiens GN=HIST1H2AB PE=1 SV=2	sp|P04908|H2A1B_HUMAN (+7)
60	Ig kappa chain C region OS=Homo sapiens GN=IGKC PE=1 SV=1	sp|P01834|IGKC_HUMAN
61	Transforming growth factor-beta-induced protein ig-h3 OS=Homo sapiens GN=TGFBI PE=1 SV=1	sp|Q15582|BGH3_HUMAN (+1)
62	Isoform 4 of Myosin-10 OS=Homo sapiens GN=MYH10	sp|P35580-4|MYH10_HUMAN

63	Isoform 3 of Spectrin alpha chain, non-erythrocytic 1 OS=Homo sapiens GN=SPTAN1	sp|Q13813-3|SPTN1_HUMAN (+1)
64	Ig gamma-1 chain C region OS=Homo sapiens GN=IGHG1 PE=1 SV=1	sp|P01857|IGHG1_HUMAN
65	Creatine kinase B-type OS=Homo sapiens GN=CKB PE=1 SV=1	sp|P12277|KCRB_HUMAN
66	Laminin subunit alpha-2 OS=Homo sapiens GN=LAMA2 PE=1 SV=4	sp|P24043|LAMA2_HUMAN
67	Protein disulfide-isomerase OS=Homo sapiens GN=P4HB PE=1 SV=3	sp|P07237|PDIA1_HUMAN
68	ATP synthase F(0) complex subunit C1, mitochondrial OS=Homo sapiens GN=ATP5G1 PE=2 SV=2	sp|P05496|AT5G1_HUMAN (+7)
69	Endoplasmin OS=Homo sapiens GN=HSP90B1 PE=1 SV=1	sp|P14625|ENPL_HUMAN
70	Keratin, type II cytoskeletal 2 epidermal OS=Homo sapiens GN=KRT2 PE=1 SV=2	sp|P35908|K22E_HUMAN
71	Heat shock protein HSP 90-beta OS=Homo sapiens GN=HSP90AB1 PE=1 SV=4	sp|P08238|HS90B_HUMAN
72	Isoform 3 of Fermitin family homolog 2 OS=Homo sapiens GN=FERMT2	sp|Q96AC1-3|FERM2_HUMAN (+2)
73	Calponin-1 OS=Homo sapiens GN=CNN1 PE=1 SV=2	sp|P51911|CNN1_HUMAN
74	Myosin regulatory light polypeptide 9 OS=Homo sapiens GN=MYL9 PE=1 SV=4	sp|P24844|MYL9_HUMAN
75	Transitional endoplasmic reticulum ATPase OS=Homo sapiens GN=VCP PE=1 SV=4	sp|P55072|TERA_HUMAN
76	Isoform 2 of Glyceraldehyde-3-phosphate dehydrogenase OS=Homo sapiens GN=GAPDH	sp|P04406-2|G3P_HUMAN (+2)
77	Cysteine and glycine-rich protein 1 OS=Homo sapiens GN=CSRP1 PE=1 SV=3	sp|P21291|CSRP1_HUMAN
78	Heat shock cognate 71 kDa protein OS=Homo sapiens GN=HSPA8 PE=1 SV=1	sp|P11142|HSP7C_HUMAN
79	Isoform 3 of Palladin OS=Homo sapiens GN=PALLD	sp|Q8WX93-3|PALLD_HUMAN (+1)
80	Ubiquitin-like modifier-activating enzyme 1 OS=Homo sapiens GN=UBA1 PE=1 SV=3	sp|P22314|UBA1_HUMAN
81	Peroxidasin homolog OS=Homo sapiens GN=PXDN PE=1 SV=2	sp|Q92626|PXDN_HUMAN
82	Myosin light polypeptide 6 OS=Homo sapiens GN=MYL6 PE=1 SV=2	sp|P60660|MYL6_HUMAN (+1)
83	Prolow-density lipoprotein receptor-related protein 1 OS=Homo sapiens GN=LRP1 PE=1 SV=2	sp|Q07954|LRP1_HUMAN
84	Elongation factor 1-alpha 1 OS=Homo sapiens GN=EEF1A1 PE=1 SV=1	sp|P68104|EF1A1_HUMAN (+1)
85	Isoform 2 of Nidogen-1 OS=Homo sapiens GN=NID1	sp|P14543-2|NID1_HUMAN (+1)
86	Adenylyl cyclase-associated protein 1 OS=Homo sapiens GN=CAP1 PE=1 SV=5	sp|Q01518|CAP1_HUMAN
87	78 kDa glucose-regulated protein OS=Homo sapiens GN=HSPA5 PE=1 SV=2	sp|P11021|GRP78_HUMAN
88	14-3-3 protein zeta/delta OS=Homo sapiens GN=YWHAZ PE=1 SV=1	sp|P63104|1433Z_HUMAN
89	Ig gamma-3 chain C region OS=Homo sapiens GN=IGHG3 PE=1 SV=2	sp|P01860|IGHG3_HUMAN
90	Histone H4 OS=Homo sapiens GN=HIST1H4A PE=1 SV=2	sp|P62805|H4_HUMAN
91	Isoform 2 of Laminin subunit alpha-4 OS=Homo sapiens GN=LAMA4	sp|Q16363-2|LAMA4_HUMAN (+1)
92	Profilin-1 OS=Homo sapiens GN=PFN1 PE=1 SV=2	sp|P07737|PROF1_HUMAN
93	Prolargin OS=Homo sapiens GN=PRELP PE=1 SV=1	sp|P51888|PRELP_HUMAN
94	Isoform Short of Spectrin beta chain, non-erythrocytic 1 OS=Homo sapiens GN=SPTBN1	sp|Q01082-2|SPTB2_HUMAN (+2)
95	Alpha-1-antichymotrypsin OS=Homo sapiens GN=SERPINA3 PE=1 SV=2	sp|P01011|AACT_HUMAN (+1)

96	Isoform 2 of DNA-dependent protein kinase catalytic subunit OS=Homo sapiens GN=PRKDC	sp|P78527-2|PRKDC_HUMAN (+2)
97	Neuroblast differentiation-associated protein AHNAK OS=Homo sapiens GN=AHNAK PE=1 SV=2	sp|Q09666|AHNK_HUMAN
98	Isoform 2 of Heat shock protein HSP 90-alpha OS=Homo sapiens GN=HSP90AA1	sp|P07900-2|HS90A_HUMAN (+1)
99	Ceruloplasmin OS=Homo sapiens GN=CP PE=1 SV=1	sp|P00450|CERU_HUMAN
100	Cofilin-1 OS=Homo sapiens GN=CFL1 PE=1 SV=3	sp|P23528|COF1_HUMAN (+1)
101	Isoform B2 of Smoothelin OS=Homo sapiens GN=SMTN	sp|P53814-5|SMTN_HUMAN (+1)
102	Lipoma-preferred partner OS=Homo sapiens GN=LPP PE=1 SV=1	sp|Q93052|LPP_HUMAN
103	Stress-induced-phosphoprotein 1 OS=Homo sapiens GN=STIP1 PE=1 SV=1	sp|P31948|STIP1_HUMAN (+2)
104	Annexin A5 OS=Homo sapiens GN=ANXA5 PE=1 SV=2	sp|P08758|ANXA5_HUMAN
105	Protein disulfide-isomerase A3 OS=Homo sapiens GN=PDIA3 PE=1 SV=4	sp|P30101|PDIA3_HUMAN (+1)
106	Isoform 2 of Tropomyosin alpha-4 chain OS=Homo sapiens GN=TPM4	sp|P67936-2|TPM4_HUMAN
107	Staphylococcal nuclease domain-containing protein 1 OS=Homo sapiens GN=SND1 PE=1 SV=1	sp|Q7KZF4|SND1_HUMAN
108	Antithrombin-III OS=Homo sapiens GN=SERPINC1 PE=1 SV=1	sp|P01008|ANT3_HUMAN
109	Isoform 2 of Synaptopodin-2 OS=Homo sapiens GN=SYNPO2	sp|Q9UMS6-2|SYNP2_HUMAN (+3)
110	Heterogeneous nuclear ribonucleoprotein U-like protein 2 OS=Homo sapiens GN=HNRNPUL2 PE=1 SV=1	sp|Q1KMD3|HNRL2_HUMAN (+1)
111	Apolipoprotein A-I OS=Homo sapiens GN=APOA1 PE=1 SV=1	sp|P02647|APOA1_HUMAN
112	Histone H2B type 1-J OS=Homo sapiens GN=HIST1H2BJ PE=1 SV=3	sp|P06899|H2B1J_HUMAN (+3)
113	Isoform 12 of Titin OS=Homo sapiens GN=TTN	sp|Q8WZ42-12|TITIN_HUMAN
114	Versican core protein OS=Homo sapiens GN=VCAN PE=1 SV=3	sp|P13611|CSPG2_HUMAN
115	Lamin-B2 OS=Homo sapiens GN=LMNB2 PE=1 SV=3	sp|Q03252|LMNB2_HUMAN (+1)
116	Complement C4-A OS=Homo sapiens GN=C4A PE=1 SV=2	sp|P0C0L4|CO4A_HUMAN
117	Biglycan OS=Homo sapiens GN=BGN PE=1 SV=2	sp|P21810|PGS1_HUMAN (+1)
118	WD repeat-containing protein 1 OS=Homo sapiens GN=WDR1 PE=1 SV=4	sp|O75083|WDR1_HUMAN
119	Elongation factor 2 OS=Homo sapiens GN=EEF2 PE=1 SV=4	sp|P13639|EF2_HUMAN
120	Adipocyte enhancer-binding protein 1 OS=Homo sapiens GN=AEBP1 PE=1 SV=1	sp|Q8IUX7|AEBP1_HUMAN
121	X-ray repair cross-complementing protein 6 OS=Homo sapiens GN=XRCC6 PE=1 SV=2	sp|P12956|XRCC6_HUMAN (+1)
122	Rab GDP dissociation inhibitor beta OS=Homo sapiens GN=GDI2 PE=1 SV=2	sp|P50395|GDIB_HUMAN (+1)
123	Calreticulin OS=Homo sapiens GN=CALR PE=1 SV=1	sp|P27797|CALR_HUMAN
124	Isoform 2 of Testin OS=Homo sapiens GN=TES	sp|Q9UGI8-2|TES_HUMAN (+1)
125	Isoform XA of Plasma membrane calcium-transporting ATPase 4 OS=Homo sapiens GN=ATP2B4	sp|P23634-2|AT2B4_HUMAN (+7)
126	Microtubule-actin cross-linking factor 1, isoforms 1/2/3/5 OS=Homo sapiens GN=MACF1 PE=2 SV=1	tr|H3BQK9|H3BQK9_HUMAN (+1)

127	Isoform 2 of Transketolase OS=Homo sapiens GN=TKT	sp|P29401-2|TKT_HUMAN (+2)
128	Isoform 3 of Vitamin D-binding protein OS=Homo sapiens GN=GC	sp|P02774-3|VTDB_HUMAN (+2)
129	POTE ankyrin domain family member I OS=Homo sapiens GN=POTEI PE=3 SV=1	sp|P0CG38|POTEI_HUMAN
130	Hemoglobin subunit alpha OS=Homo sapiens GN=HBA1 PE=1 SV=2	sp|P69905|HBA_HUMAN
131	Isoform 2 of Heterogeneous nuclear ribonucleoprotein K OS=Homo sapiens GN=HNRNPK	sp|P61978-2|HNRPK_HUMAN (+2)
132	Alpha-parvin OS=Homo sapiens GN=PARVA PE=1 SV=1	sp|Q9NVD7|PARVA_HUMAN (+1)
133	SPARC-like protein 1 OS=Homo sapiens GN=SPARCL1 PE=1 SV=2	sp|Q14515|SPRL1_HUMAN
134	X-ray repair cross-complementing protein 5 OS=Homo sapiens GN=XRCC5 PE=1 SV=3	sp|P13010|XRCC5_HUMAN
135	Immunoglobulin lambda-like polypeptide 5 OS=Homo sapiens GN=IGLL5 PE=2 SV=2	sp|B9A064|IGLL5_HUMAN
136	Integrin beta-1 OS=Homo sapiens GN=ITGB1 PE=1 SV=2	sp|P05556|ITB1_HUMAN
137	Heat shock-related 70 kDa protein 2 OS=Homo sapiens GN=HSPA2 PE=1 SV=1	sp|P54652|HSP72_HUMAN
138	Isoform 2 of Leiomodin-1 OS=Homo sapiens GN=LMOD1	sp|P29536-2|LMOD1_HUMAN (+1)
139	Isoform 2 of ATP synthase subunit alpha, mitochondrial OS=Homo sapiens GN=ATP5A1	sp|P25705-2|ATPA_HUMAN (+1)
140	Peroxiredoxin-6 OS=Homo sapiens GN=PRDX6 PE=1 SV=3	sp|P30041|PRDX6_HUMAN
141	Nucleolin OS=Homo sapiens GN=NCL PE=1 SV=3	sp|P19338|NUCL_HUMAN
142	Serine/threonine-protein phosphatase 2A 65 kDa regulatory subunit A alpha isoform OS=Homo sapiens GN=PPP2R1A PE=1 SV=4	sp|P30153|2AAA_HUMAN (+1)
143	Isoform 4 of Four and a half LIM domains protein 1 OS=Homo sapiens GN=FHL1	sp|Q13642-4|FHL1_HUMAN (+1)
144	Isoform 2 of Fructose-bisphosphate aldolase A OS=Homo sapiens GN=ALDOA	sp|P04075-2|ALDOA_HUMAN (+2)
145	Isoform 2 of Filamin-B OS=Homo sapiens GN=FLNB	sp|O75369-2|FLNB_HUMAN (+3)
146	Decorin OS=Homo sapiens GN=DCN PE=1 SV=1	sp|P07585|PGS2_HUMAN
147	PDZ and LIM domain protein 7 OS=Homo sapiens GN=PDLIM7 PE=1 SV=1	sp|Q9NR12|PDLI7_HUMAN
148	Heat shock protein beta-1 OS=Homo sapiens GN=HSPB1 PE=1 SV=2	sp|P04792|HSPB1_HUMAN
149	Heterogeneous nuclear ribonucleoprotein R OS=Homo sapiens GN=HNRNPR PE=1 SV=1	sp|O43390|HNRPR_HUMAN
150	Laminin subunit alpha-5 OS=Homo sapiens GN=LAMA5 PE=1 SV=8	sp|O15230|LAMA5_HUMAN
151	Isoform 3 of Malate dehydrogenase, cytoplasmic OS=Homo sapiens GN=MDH1	sp|P40925-3|MDHC_HUMAN (+1)
152	Calnexin OS=Homo sapiens GN=CANX PE=1 SV=2	sp|P27824|CALX_HUMAN (+1)
153	ATP-dependent RNA helicase A OS=Homo sapiens GN=DHX9 PE=1 SV=4	sp|Q08211|DHX9_HUMAN
154	EH domain-containing protein 2 OS=Homo sapiens GN=EHD2 PE=1 SV=2	sp|Q9NZN4|EHD2_HUMAN
155	Complement factor H OS=Homo sapiens GN=CFH PE=1 SV=4	sp|P08603|CFAH_HUMAN
156	Hemopexin OS=Homo sapiens GN=HPX PE=1 SV=2	sp|P02790|HEMO_HUMAN
157	Phosphoglycerate kinase 1 OS=Homo sapiens GN=PGK1 PE=1 SV=3	sp|P00558|PGK1_HUMAN (+1)
158	Haptoglobin OS=Homo sapiens GN=HP PE=1 SV=1	sp|P00738|HPT_HUMAN (+1)

159	Isoform 2 of KN motif and ankyrin repeat domain-containing protein 2 OS=Homo sapiens GN=KANK2	sp|Q63ZY3-2|KANK2_HUMAN (+2)
160	EMILIN-1 OS=Homo sapiens GN=EMILIN1 PE=1 SV=2	sp|Q9Y6C2|EMIL1_HUMAN
161	Laminin subunit beta-2 OS=Homo sapiens GN=LAMB2 PE=1 SV=2	sp|P55268|LAMB2_HUMAN
162	Isoform 3 of Unconventional myosin-Ic OS=Homo sapiens GN=MYO1C	sp|O00159-3|MYO1C_HUMAN (+2)
163	60 kDa heat shock protein, mitochondrial OS=Homo sapiens GN=HSPD1 PE=1 SV=2	sp|P10809|CH60_HUMAN
164	Isoform 2 of Glucose-6-phosphate isomerase OS=Homo sapiens GN=GPI	sp|P06744-2|G6PI_HUMAN (+1)
165	Isoform C1 of Heterogeneous nuclear ribonucleoproteins C1/C2 OS=Homo sapiens GN=HNRNPC	sp|P07910-2|HNRPC_HUMAN (+4)
166	Ras-related protein Rap-1A OS=Homo sapiens GN=RAP1A PE=1 SV=1	sp|P62834|RAP1A_HUMAN
167	Serpin H1 OS=Homo sapiens GN=SERPINH1 PE=1 SV=2	sp|P50454|SERPH_HUMAN
168	Polyubiquitin-B OS=Homo sapiens GN=UBB PE=1 SV=1	sp|P0CG47|UBB_HUMAN (+17)
169	Isoform 2 of Protein disulfide-isomerase A6 OS=Homo sapiens GN=PDIA6	sp|Q15084-2|PDIA6_HUMAN (+4)
170	Rho GTPase-activating protein 1 OS=Homo sapiens GN=ARHGAP1 PE=1 SV=1	sp|Q07960|RHG01_HUMAN
171	Stress-70 protein, mitochondrial OS=Homo sapiens GN=HSPA9 PE=1 SV=2	sp|P38646|GRP75_HUMAN
172	UTP--glucose-1-phosphate uridylyltransferase OS=Homo sapiens GN=UGP2 PE=1 SV=5	sp|Q16851|UGPA_HUMAN (+1)
173	Tensin-1 OS=Homo sapiens GN=TNS1 PE=1 SV=2	sp|Q9HBL0|TENS1_HUMAN (+2)
174	Isoform 4 of Myosin-11 OS=Homo sapiens GN=MYH11	sp|P35749-4|MYH11_HUMAN
175	Isoform 3 of Collagen alpha-1(XVIII) chain OS=Homo sapiens GN=COL18A1	sp|P39060-2|COIA1_HUMAN
176	Importin subunit beta-1 OS=Homo sapiens GN=KPNB1 PE=1 SV=2	sp|Q14974|IMB1_HUMAN
177	Integrin-linked protein kinase OS=Homo sapiens GN=ILK PE=1 SV=2	sp|Q13418|ILK_HUMAN
178	Calmodulin OS=Homo sapiens GN=CALM1 PE=1 SV=2	sp|P62158|CALM_HUMAN (+3)
179	BTB/POZ domain-containing protein KCTD12 OS=Homo sapiens GN=KCTD12 PE=1 SV=1	sp|Q96CX2|KCD12_HUMAN
180	Heterogeneous nuclear ribonucleoprotein D0 OS=Homo sapiens GN=HNRNPD PE=2 SV=1	tr|B4DTC3|B4DTC3_HUMAN
181	Collagen alpha-2(IV) chain OS=Homo sapiens GN=COL4A2 PE=1 SV=4	sp|P08572|CO4A2_HUMAN
182	Collagen alpha-1(III) chain OS=Homo sapiens GN=COL3A1 PE=1 SV=4	sp|P02461|CO3A1_HUMAN
183	Polymerase I and transcript release factor OS=Homo sapiens GN=PTRF PE=1 SV=1	sp|Q6NZI2|PTRF_HUMAN
184	Eukaryotic initiation factor 4A-I OS=Homo sapiens GN=EIF4A1 PE=1 SV=1	sp|P60842|IF4A1_HUMAN
185	Isoform 3 of Drebrin OS=Homo sapiens GN=DBN1	sp|Q16643-3|DREB_HUMAN (+1)
186	Alanine--tRNA ligase, cytoplasmic OS=Homo sapiens GN=AARS PE=1 SV=2	sp|P49588|SYAC_HUMAN
187	Isoform 2 of Coronin-1C OS=Homo sapiens GN=CORO1C	sp|Q9ULV4-2|COR1C_HUMAN (+2)
188	Integrin alpha-1 OS=Homo sapiens GN=ITGA1 PE=1 SV=2	sp|P56199|ITA1_HUMAN
189	Integrin alpha-5 OS=Homo sapiens GN=ITGA5 PE=1 SV=2	sp|P08648|ITA5_HUMAN
190	Isoform 2 of Annexin A2 OS=Homo sapiens GN=ANXA2	sp|P07355-2|ANXA2_HUMAN (+1)

191	Fibrillin-1 OS=Homo sapiens GN=FBN1 PE=1 SV=3	sp|P35555|FBN1_HUMAN
192	Isoform 2 of Septin-7 OS=Homo sapiens GN=SEPT7	sp|Q16181-2|SEPT7_HUMAN (+4)
193	Non-POU domain-containing octamer-binding protein OS=Homo sapiens GN=NONO PE=1 SV=4	sp|Q15233|NONO_HUMAN
194	Collagen alpha-2(V) chain OS=Homo sapiens GN=COL5A2 PE=1 SV=3	sp|P05997|CO5A2_HUMAN
195	High mobility group protein B1 OS=Homo sapiens GN=HMGB1 PE=1 SV=3	sp|P09429|HMGB1_HUMAN (+2)
196	Isoform 2 of Kinectin OS=Homo sapiens GN=KTN1	sp|Q86UP2-2|KTN1_HUMAN (+1)
197	Alpha-1B-glycoprotein OS=Homo sapiens GN=A1BG PE=1 SV=4	sp|P04217|A1BG_HUMAN
198	Isoform 2 of Nuclear mitotic apparatus protein 1 OS=Homo sapiens GN=NUMA1	sp|Q14980-2|NUMA1_HUMAN (+1)
199	Cytochrome c oxidase subunit 7A2, mitochondrial OS=Homo sapiens GN=COX7A2 PE=1 SV=1	sp|P14406|CX7A2_HUMAN (+1)
200	Brain acid soluble protein 1 OS=Homo sapiens GN=BASP1 PE=1 SV=2	sp|P80723|BASP1_HUMAN
201	Dihydropyrimidinase-related protein 2 OS=Homo sapiens GN=DPYSL2 PE=1 SV=1	sp|Q16555|DPYL2_HUMAN
202	Mimecan OS=Homo sapiens GN=OGN PE=1 SV=1	sp|P20774|MIME_HUMAN
203	Isoform 3 of Dynactin subunit 1 OS=Homo sapiens GN=DCTN1	sp|Q14203-3|DCTN1_HUMAN (+4)
204	Angiotensinogen OS=Homo sapiens GN=AGT PE=1 SV=1	sp|P01019|ANGT_HUMAN
205	Chondroitin sulfate proteoglycan 4 OS=Homo sapiens GN=CSPG4 PE=1 SV=2	sp|Q6UVK1|CSPG4_HUMAN
206	Isochorismatase domain-containing protein 1 OS=Homo sapiens GN=ISOC1 PE=1 SV=3	sp|Q96CN7|ISOC1_HUMAN
207	14-3-3 protein epsilon OS=Homo sapiens GN=YWHAE PE=1 SV=1	sp|P62258|1433E_HUMAN
208	Caveolin-1 OS=Homo sapiens GN=CAV1 PE=1 SV=4	sp|Q03135|CAV1_HUMAN (+1)
209	Lamin-B1 OS=Homo sapiens GN=LMNB1 PE=1 SV=2	sp|P20700|LMNB1_HUMAN
210	Collagen alpha-1(XV) chain OS=Homo sapiens GN=COL15A1 PE=1 SV=2	sp|P39059|COFA1_HUMAN
211	Isoform 3 of L-lactate dehydrogenase A chain OS=Homo sapiens GN=LDHA	sp|P00338-3|LDHA_HUMAN (+1)
212	L-lactate dehydrogenase B chain OS=Homo sapiens GN=LDHB PE=1 SV=2	sp|P07195|LDHB_HUMAN
213	Isoform 2 of Hydroxyacyl-coenzyme A dehydrogenase, mitochondrial OS=Homo sapiens GN=HADH	sp|Q16836-2|HCDH_HUMAN (+3)
214	Coagulation factor XIII A chain OS=Homo sapiens GN=F13A1 PE=1 SV=4	sp|P00488|F13A_HUMAN
215	Isoform 3 of Dystrophin OS=Homo sapiens GN=DMD	sp|P11532-4|DMD_HUMAN (+2)
216	Isoform Short of Ubiquitin carboxyl-terminal hydrolase 5 OS=Homo sapiens GN=USP5	sp|P45974-2|UBP5_HUMAN (+1)
217	Heterogeneous nuclear ribonucleoprotein L OS=Homo sapiens GN=HNRNPL PE=1 SV=2	sp|P14866|HNRPL_HUMAN
218	Fibulin-1 OS=Homo sapiens GN=FBLN1 PE=1 SV=4	sp|P23142|FBLN1_HUMAN
219	Isoform 2 of Spliceosome RNA helicase DDX39B OS=Homo sapiens GN=DDX39B	sp|Q13838-2|DX39B_HUMAN (+2)
220	Ig alpha-1 chain C region OS=Homo sapiens GN=IGHA1 PE=1 SV=2	sp|P01876|IGHA1_HUMAN
221	Isoform 2 of Septin-2 OS=Homo sapiens GN=SEPT2	sp|Q15019-2|SEPT2_HUMAN (+1)
222	Hypoxia up-regulated protein 1 OS=Homo sapiens GN=HYOU1 PE=1 SV=1	sp|Q9Y4L1|HYOU1_HUMAN (+1)

223	Trifunctional enzyme subunit alpha, mitochondrial OS=Homo sapiens GN=HADHA PE=1 SV=2	sp|P40939|ECHA_HUMAN
224	Neutral alpha-glucosidase AB OS=Homo sapiens GN=GANAB PE=1 SV=3	sp|Q14697|GANAB_HUMAN (+1)
225	Isoform 2 of Polypyrimidine tract-binding protein 1 OS=Homo sapiens GN=PTBP1	sp|P26599-2|PTBP1_HUMAN (+2)
226	Isoform LMW of Kininogen-1 OS=Homo sapiens GN=KNG1	sp|P01042-2|KNG1_HUMAN (+2)
227	Protein-glutamine gamma-glutamyltransferase 2 OS=Homo sapiens GN=TGM2 PE=1 SV=2	sp|P21980|TGM2_HUMAN (+1)
228	Isoform 2 of Triosephosphate isomerase OS=Homo sapiens GN=TPI1	sp|P60174-1|TPIS_HUMAN (+1)
229	DNA damage-binding protein 1 OS=Homo sapiens GN=DDB1 PE=1 SV=1	sp|Q16531|DDB1_HUMAN (+1)
230	Protein disulfide-isomerase A4 OS=Homo sapiens GN=PDIA4 PE=1 SV=2	sp|P13667|PDIA4_HUMAN
231	Pigment epithelium-derived factor OS=Homo sapiens GN=SERPINF1 PE=1 SV=4	sp|P36955|PEDF_HUMAN
232	14-3-3 protein gamma OS=Homo sapiens GN=YWHAG PE=1 SV=2	sp|P61981|1433G_HUMAN
233	Glutamate dehydrogenase 1, mitochondrial OS=Homo sapiens GN=GLUD1 PE=1 SV=2	sp|P00367|DHE3_HUMAN
234	Isoform 2 of Transforming growth factor beta-1-induced transcript 1 protein OS=Homo sapiens GN=TGFB1I1	sp|O43294-2|TGFI1_HUMAN (+1)
235	Tripartite motif-containing protein 47 OS=Homo sapiens GN=TRIM47 PE=1 SV=2	sp|Q96LD4|TRI47_HUMAN
236	Ig lambda-2 chain C regions OS=Homo sapiens GN=IGLC2 PE=1 SV=1	sp|P0CG05|LAC2_HUMAN (+1)
237	Isoform Short of Heterogeneous nuclear ribonucleoprotein U OS=Homo sapiens GN=HNRNPU	sp|Q00839-2|HNRPU_HUMAN (+1)
238	Microtubule-associated protein 1B OS=Homo sapiens GN=MAP1B PE=1 SV=2	sp|P46821|MAP1B_HUMAN
239	Isoform 3 of Laminin subunit alpha-3 OS=Homo sapiens GN=LAMA3	sp|Q16787-3|LAMA3_HUMAN (+1)
240	Aconitate hydratase, mitochondrial OS=Homo sapiens GN=ACO2 PE=1 SV=2	sp|Q99798|ACON_HUMAN (+1)
241	26S proteasome non-ATPase regulatory subunit 2 OS=Homo sapiens GN=PSMD2 PE=1 SV=3	sp|Q13200|PSMD2_HUMAN (+1)
242	Beta-2-glycoprotein 1 OS=Homo sapiens GN=APOH PE=1 SV=3	sp|P02749|APOH_HUMAN
243	Fibulin-5 OS=Homo sapiens GN=FBLN5 PE=1 SV=1	sp|Q9UBX5|FBLN5_HUMAN (+3)
244	Isoform 3 of Importin-5 OS=Homo sapiens GN=IPO5	sp|O00410-3|IPO5_HUMAN (+2)
245	Actin-related protein 3 OS=Homo sapiens GN=ACTR3 PE=1 SV=3	sp|P61158|ARP3_HUMAN (+1)
246	Isoform 1 of Ribosome-binding protein 1 OS=Homo sapiens GN=RRBP1	sp|Q9P2E9-2|RRBP1_HUMAN (+1)
247	Asporin OS=Homo sapiens GN=ASPN PE=1 SV=2	sp|Q9BXN1|ASPN_HUMAN
248	Peptidyl-prolyl cis-trans isomerase FKBP10 OS=Homo sapiens GN=FKBP10 PE=1 SV=1	sp|Q96AY3|FKB10_HUMAN (+1)
249	T-complex protein 1 subunit beta OS=Homo sapiens GN=CCT2 PE=1 SV=4	sp|P78371|TCPB_HUMAN
250	Leucine--tRNA ligase, cytoplasmic OS=Homo sapiens GN=LARS PE=1 SV=2	sp|Q9P2J5|SYLC_HUMAN (+3)
251	Glutathione S-transferase P OS=Homo sapiens GN=GSTP1 PE=1 SV=2	sp|P09211|GSTP1_HUMAN
252	Transaldolase OS=Homo sapiens GN=TALDO1 PE=1 SV=2	sp|P37837|TALDO_HUMAN
253	Myosin regulatory light chain 12B OS=Homo sapiens GN=MYL12B PE=1 SV=2	sp|O14950|ML12B_HUMAN (+2)
254	LIM and SH3 domain protein 1 OS=Homo sapiens GN=LASP1 PE=1 SV=2	sp|Q14847|LASP1_HUMAN

255	Poly(rC)-binding protein 1 OS=Homo sapiens GN=PCBP1 PE=1 SV=2	sp|Q15365|PCBP1_HUMAN
256	Isoform 4 of CD109 antigen OS=Homo sapiens GN=CD109	sp|Q6YHK3-4|CD109_HUMAN (+1)
257	Isoform 2 of Tubulin alpha-1A chain OS=Homo sapiens GN=TUBA1A	sp|Q71U36-2|TBA1A_HUMAN (+1)
258	Keratin, type I cytoskeletal 14 OS=Homo sapiens GN=KRT14 PE=1 SV=4	sp|P02533|K1C14_HUMAN
259	Isoform 2 of Heterogeneous nuclear ribonucleoprotein M OS=Homo sapiens GN=HNRNPM	sp|P52272-2|HNRPM_HUMAN (+1)
260	Isoform 2 of Eukaryotic translation initiation factor 3 subunit B OS=Homo sapiens GN=EIF3B	sp|P55884-2|EIF3B_HUMAN (+1)
261	Isoform 2 of Calumenin OS=Homo sapiens GN=CALU	sp|O43852-2|CALU_HUMAN (+1)
262	Complement factor B OS=Homo sapiens GN=CFB PE=1 SV=2	sp|P00751|CFAB_HUMAN (+1)
263	Puromycin-sensitive aminopeptidase OS=Homo sapiens GN=NPEPPS PE=1 SV=2	sp|P55786|PSA_HUMAN (+2)
264	Isoform 2 of Collagen alpha-1(VII) chain OS=Homo sapiens GN=COL7A1	sp|Q02388-2|CO7A1_HUMAN (+1)
265	Plastin-3 OS=Homo sapiens GN=PLS3 PE=1 SV=4	sp|P13797|PLST_HUMAN
266	Amine oxidase [flavin-containing] B OS=Homo sapiens GN=MAOB PE=1 SV=3	sp|P27338|AOFB_HUMAN (+1)
267	Isoform A2 of Heterogeneous nuclear ribonucleoproteins A2/B1 OS=Homo sapiens GN=HNRNPA2B1	sp|P22626-2|ROA2_HUMAN (+1)
268	Protein DJ-1 (Fragment) OS=Homo sapiens GN=PARK7 PE=2 SV=1	tr|K7EN27|K7EN27_HUMAN
269	Alcohol dehydrogenase class-3 OS=Homo sapiens GN=ADH5 PE=1 SV=4	sp|P11766|ADHX_HUMAN
270	Isoform 3 of Nucleoside diphosphate kinase B OS=Homo sapiens GN=NME2	sp|P22392-2|NDKB_HUMAN (+1)
271	Prolyl endopeptidase OS=Homo sapiens GN=PREP PE=1 SV=2	sp|P48147|PPCE_HUMAN
272	Ig gamma-2 chain C region OS=Homo sapiens GN=IGHG2 PE=1 SV=2	sp|P01859|IGHG2_HUMAN
273	40S ribosomal protein SA OS=Homo sapiens GN=RPSA PE=1 SV=4	sp|P08865|RSSA_HUMAN (+1)
274	Membrane primary amine oxidase OS=Homo sapiens GN=AOC3 PE=1 SV=3	sp|Q16853|AOC3_HUMAN
275	Apolipoprotein B-100 OS=Homo sapiens GN=APOB PE=1 SV=2	sp|P04114|APOB_HUMAN
276	Isoform 2 of Septin-9 OS=Homo sapiens GN=SEPT9	sp|Q9UHD8-2|SEPT9_HUMAN (+4)
277	Peroxiredoxin-1 OS=Homo sapiens GN=PRDX1 PE=1 SV=1	sp|Q06830|PRDX1_HUMAN
278	Cullin-associated NEDD8-dissociated protein 1 OS=Homo sapiens GN=CAND1 PE=1 SV=2	sp|Q86VP6|CAND1_HUMAN
279	Isoform 2 of 26S proteasome non-ATPase regulatory subunit 1 OS=Homo sapiens GN=PSMD1	sp|Q99460-2|PSMD1_HUMAN (+1)
280	Elongation factor 1-gamma OS=Homo sapiens GN=EEF1G PE=2 SV=1	tr|B4DTG2|B4DTG2_HUMAN (+1)
281	Dystonin OS=Homo sapiens GN=DST PE=2 SV=2	tr|E9PEB9|E9PEB9_HUMAN-R
282	Hsp90 co-chaperone Cdc37 OS=Homo sapiens GN=CDC37 PE=1 SV=1	sp|Q16543|CDC37_HUMAN
283	Isoform 2B of Cytoplasmic dynein 1 intermediate chain 2 OS=Homo sapiens GN=DYNC1I2	sp|Q13409-2|DC1I2_HUMAN (+6)
284	Inter-alpha-trypsin inhibitor heavy chain H1 OS=Homo sapiens GN=ITIH1 PE=1 SV=3	sp|P19827|ITIH1_HUMAN (+1)
285	Synaptic vesicle membrane protein VAT-1 homolog OS=Homo sapiens GN=VAT1 PE=1 SV=2	sp|Q99536|VAT1_HUMAN
286	von Willebrand factor OS=Homo sapiens GN=VWF PE=1 SV=4	sp|P04275|VWF_HUMAN

287	AP-2 complex subunit beta OS=Homo sapiens GN=AP2B1 PE=2 SV=1	tr|K7EJT8|K7EJT8_HUMAN
288	U5 small nuclear ribonucleoprotein 200 kDa helicase OS=Homo sapiens GN=SNRNP200 PE=1 SV=2	sp|O75643|U520_HUMAN
289	Glycogen phosphorylase, brain form OS=Homo sapiens GN=PYGB PE=1 SV=5	sp|P11216|PYGB_HUMAN
290	Fascin OS=Homo sapiens GN=FSCN1 PE=1 SV=3	sp|Q16658|FSCN1_HUMAN (+1)
291	Isoform 2 of F-actin-capping protein subunit beta OS=Homo sapiens GN=CAPZB	sp|P47756-2|CAPZB_HUMAN (+2)
292	Splicing factor 3B subunit 3 OS=Homo sapiens GN=SF3B3 PE=1 SV=4	sp|Q15393|SF3B3_HUMAN
293	Eukaryotic initiation factor 4A-III OS=Homo sapiens GN=EIF4A3 PE=1 SV=4	sp|P38919|IF4A3_HUMAN
294	Isoform 2 of Alpha-aminoadipic semialdehyde dehydrogenase OS=Homo sapiens GN=ALDH7A1	sp|P49419-2|AL7A1_HUMAN
295	Hemoglobin subunit beta OS=Homo sapiens GN=HBB PE=1 SV=2	sp|P68871|HBB_HUMAN
296	Isoform 2 of 116 kDa U5 small nuclear ribonucleoprotein component OS=Homo sapiens GN=EFTUD2	sp|Q15029-2|U5S1_HUMAN
297	Isoform 2 of Programmed cell death 6-interacting protein OS=Homo sapiens GN=PDCD6IP	sp|Q8WUM4-2|PDC6I_HUMAN (+1)
298	Ras suppressor protein 1 OS=Homo sapiens GN=RSU1 PE=1 SV=3	sp|Q15404|RSU1_HUMAN
299	14-3-3 protein theta OS=Homo sapiens GN=YWHAQ PE=1 SV=1	sp|P27348|1433T_HUMAN
300	Isoform 1 of Core histone macro-H2A.1 OS=Homo sapiens GN=H2AFY	sp|O75367-2|H2AY_HUMAN
301	Parathymosin OS=Homo sapiens GN=PTMS PE=1 SV=2	sp|P20962|PTMS_HUMAN
302	Peptidyl-prolyl cis-trans isomerase B OS=Homo sapiens GN=PPIB PE=1 SV=2	sp|P23284|PPIB_HUMAN
303	Heat shock 70 kDa protein 4 OS=Homo sapiens GN=HSPA4 PE=1 SV=4	sp|P34932|HSP74_HUMAN
304	Splicing factor, proline- and glutamine-rich OS=Homo sapiens GN=SFPQ PE=1 SV=2	sp|P23246|SFPQ_HUMAN
305	Dihydropyrimidinase-related protein 1 OS=Homo sapiens GN=CRMP1 PE=1 SV=1	sp|Q14194|DPYL1_HUMAN
306	Fibromodulin OS=Homo sapiens GN=FMOD PE=1 SV=2	sp|Q06828|FMOD_HUMAN
307	Coatomer subunit gamma-1 OS=Homo sapiens GN=COPG1 PE=1 SV=1	sp|Q9Y678|COPG1_HUMAN
308	Isoform 3 of Protein flightless-1 homolog OS=Homo sapiens GN=FLII	sp|Q13045-3|FLII_HUMAN (+2)
309	Isoform 2 of 2-oxoglutarate dehydrogenase, mitochondrial OS=Homo sapiens GN=OGDH	sp|Q02218-2|ODO1_HUMAN (+4)
310	Isoform 2 of Ig mu chain C region OS=Homo sapiens GN=IGHM	sp|P01871-2|IGHM_HUMAN (+1)
311	Tropomodulin-1 OS=Homo sapiens GN=TMOD1 PE=1 SV=1	sp|P28289|TMOD1_HUMAN
312	Phosphoglucomutase-2 OS=Homo sapiens GN=PGM2 PE=1 SV=4	sp|Q96G03|PGM2_HUMAN
313	Supervillin OS=Homo sapiens GN=SVIL PE=1 SV=2	sp|O95425|SVIL_HUMAN (+1)
314	Vacuolar protein sorting-associated protein 35 OS=Homo sapiens GN=VPS35 PE=1 SV=2	sp|Q96QK1|VPS35_HUMAN
315	6-phosphofructokinase, liver type OS=Homo sapiens GN=PFKL PE=1 SV=6	sp|P17858|K6PL_HUMAN
316	Cytoplasmic aconitate hydratase OS=Homo sapiens GN=ACO1 PE=1 SV=3	sp|P21399|ACOC_HUMAN (+1)
317	Cell surface glycoprotein MUC18 OS=Homo sapiens GN=MCAM PE=1 SV=2	sp|P43121|MUC18_HUMAN

318	Complement component C9 OS=Homo sapiens GN=C9 PE=1 SV=2	sp|P02748|CO9_HUMAN
319	DNA-(apurinic or apyrimidinic site) lyase OS=Homo sapiens GN=APEX1 PE=1 SV=2	sp|P27695|APEX1_HUMAN
320	Malate dehydrogenase, mitochondrial OS=Homo sapiens GN=MDH2 PE=1 SV=3	sp|P40926|MDHM_HUMAN
321	60S acidic ribosomal protein P0 OS=Homo sapiens GN=RPLP0 PE=1 SV=1	sp|P05388|RLA0_HUMAN
322	Hexokinase-1 OS=Homo sapiens GN=HK1 PE=2 SV=1	tr|E7ENR4|E7ENR4_HUMAN (+4)
323	Fatty acid-binding protein, heart OS=Homo sapiens GN=FABP3 PE=1 SV=4	sp|P05413|FABPH_HUMAN (+1)
324	Structural maintenance of chromosomes protein 3 OS=Homo sapiens GN=SMC3 PE=1 SV=2	sp|Q9UQE7|SMC3_HUMAN
325	F-actin-capping protein subunit alpha-1 OS=Homo sapiens GN=CAPZA1 PE=1 SV=3	sp|P52907|CAZA1_HUMAN
326	3-mercaptopyruvate sulfurtransferase OS=Homo sapiens GN=MPST PE=1 SV=3	sp|P25325|THTM_HUMAN (+1)
327	SH3 domain-binding glutamic acid-rich-like protein OS=Homo sapiens GN=SH3BGRL PE=1 SV=1	sp|O75368|SH3L1_HUMAN
328	Dolichyl-diphosphooligosaccharide--protein glycosyltransferase subunit 2 OS=Homo sapiens GN=RPN2 PE=1 SV=3	sp|P04844|RPN2_HUMAN
329	Peptidyl-prolyl cis-trans isomerase FKBP5 OS=Homo sapiens GN=FKBP5 PE=1 SV=2	sp|Q13451|FKBP5_HUMAN
330	RuvB-like 2 OS=Homo sapiens GN=RUVBL2 PE=1 SV=3	sp|Q9Y230|RUVB2_HUMAN (+1)
331	Isoform 2 of Inter-alpha-trypsin inhibitor heavy chain H4 OS=Homo sapiens GN=ITIH4	sp|Q14624-2|ITIH4_HUMAN
332	Isoform 2 of Fibrinogen alpha chain OS=Homo sapiens GN=FGA	sp|P02671-2|FIBA_HUMAN (+1)
333	Eosinophil peroxidase OS=Homo sapiens GN=EPX PE=1 SV=2	sp|P11678|PERE_HUMAN
334	Hsc70-interacting protein OS=Homo sapiens GN=ST13 PE=1 SV=2	sp|P50502|F10A1_HUMAN (+1)
335	Isoform 3 of Heterogeneous nuclear ribonucleoprotein Q OS=Homo sapiens GN=SYNCRIP	sp|O60506-3|HNRPQ_HUMAN (+1)
336	Complement C5 OS=Homo sapiens GN=C5 PE=1 SV=4	sp|P01031|CO5_HUMAN
337	Isoform 2 of Annexin A7 OS=Homo sapiens GN=ANXA7	sp|P20073-2|ANXA7_HUMAN (+2)
338	Annexin A11 OS=Homo sapiens GN=ANXA11 PE=1 SV=1	sp|P50995|ANX11_HUMAN (+1)
339	Pre-mRNA-processing-splicing factor 8 OS=Homo sapiens GN=PRPF8 PE=1 SV=2	sp|Q6P2Q9|PRP8_HUMAN
340	Isoform 2 of Filamin A-interacting protein 1-like OS=Homo sapiens GN=FILIP1L	sp|Q4L180-2|FIL1L_HUMAN (+4)
341	Isoform 2 of Elongation factor 1-delta OS=Homo sapiens GN=EEF1D	sp|P29692-2|EF1D_HUMAN (+4)
342	Isocitrate dehydrogenase [NADP], mitochondrial OS=Homo sapiens GN=IDH2 PE=1 SV=2	sp|P48735|IDHP_HUMAN (+1)
343	Isoform 2 of Ryanodine receptor 2 OS=Homo sapiens GN=RYR2	sp|Q92736-2|RYR2_HUMAN-R (+3)
344	Alpha-1-acid glycoprotein 1 OS=Homo sapiens GN=ORM1 PE=1 SV=1	sp|P02763|A1AG1_HUMAN
345	Rab GDP dissociation inhibitor alpha OS=Homo sapiens GN=GDI1 PE=1 SV=2	sp|P31150|GDIA_HUMAN
346	Tyrosine--tRNA ligase, cytoplasmic OS=Homo sapiens GN=YARS PE=1 SV=4	sp|P54577|SYYC_HUMAN
347	Isoform 3 of T-complex protein 1 subunit eta OS=Homo sapiens GN=CCT7	sp|Q99832-3|TCPH_HUMAN (+1)
348	Isoform 2 of Vigilin OS=Homo sapiens GN=HDLBP	sp|Q00341-2|VIGLN_HUMAN (+1)
349	RuvB-like 1 OS=Homo sapiens GN=RUVBL1 PE=1 SV=1	sp|Q9Y265|RUVB1_HUMAN

350	Isoform 2 of LIM and senescent cell antigen-like-containing domain protein 1 OS=Homo sapiens GN=LIMS1	sp|P48059-2|LIMS1_HUMAN (+4)
351	Isoform 2 of Bifunctional purine biosynthesis protein PURH OS=Homo sapiens GN=ATIC	sp|P31939-2|PUR9_HUMAN (+2)
352	Histone H3.2 OS=Homo sapiens GN=HIST2H3A PE=1 SV=3	sp|Q71DI3|H32_HUMAN
353	Fibrinogen beta chain OS=Homo sapiens GN=FGB PE=1 SV=2	sp|P02675|FIBB_HUMAN
354	Isoform Monomeric of Arginine--tRNA ligase, cytoplasmic OS=Homo sapiens GN=RARS	sp|P54136-2|SYRC_HUMAN (+1)
355	Isocitrate dehydrogenase [NADP] cytoplasmic OS=Homo sapiens GN=IDH1 PE=1 SV=2	sp|O75874|IDHC_HUMAN
356	Catalase OS=Homo sapiens GN=CAT PE=1 SV=3	sp|P04040|CATA_HUMAN
357	Isoform 2 of Neural cell adhesion molecule 1 OS=Homo sapiens GN=NCAM1	sp|P13591-1|NCAM1_HUMAN (+2)
358	Isoform 2 of Dynactin subunit 2 OS=Homo sapiens GN=DCTN2	sp|Q13561-2|DCTN2_HUMAN (+5)
359	Isoform 2 of Probable ATP-dependent RNA helicase DDX17 OS=Homo sapiens GN=DDX17	sp|Q92841-1|DDX17_HUMAN (+5)
360	Cofilin-2 OS=Homo sapiens GN=CFL2 PE=1 SV=1	sp|Q9Y281|COF2_HUMAN
361	Isoform 2 of Glucosidase 2 subunit beta OS=Homo sapiens GN=PRKCSH	sp|P14314-2|GLU2B_HUMAN (+2)
362	Asparagine--tRNA ligase, cytoplasmic OS=Homo sapiens GN=NARS PE=1 SV=1	sp|O43776|SYNC_HUMAN
363	Isoform 2 of Eukaryotic translation initiation factor 5A-1 OS=Homo sapiens GN=EIF5A	sp|P63241-2|IF5A1_HUMAN (+3)
364	Isoform Short of 14-3-3 protein beta/alpha OS=Homo sapiens GN=YWHAB	sp|P31946-2|1433B_HUMAN (+1)
365	Phosphoglucomutase-1 OS=Homo sapiens GN=PGM1 PE=1 SV=3	sp|P36871|PGM1_HUMAN
366	Isoform 2 of Tryptophan--tRNA ligase, cytoplasmic OS=Homo sapiens GN=WARS	sp|P23381-2|SYWC_HUMAN (+1)
367	Isoform 2 of 5'-nucleotidase OS=Homo sapiens GN=NT5E	sp|P21589-2|5NTD_HUMAN (+1)
368	Protein AMBP OS=Homo sapiens GN=AMBP PE=1 SV=1	sp|P02760|AMBP_HUMAN (+1)
369	Zyxin OS=Homo sapiens GN=ZYX PE=1 SV=1	sp|Q15942|ZYX_HUMAN
370	Aldehyde dehydrogenase, mitochondrial OS=Homo sapiens GN=ALDH2 PE=1 SV=2	sp|P05091|ALDH2_HUMAN
371	Isoform 2 of ATP-citrate synthase OS=Homo sapiens GN=ACLY	sp|P53396-2|ACLY_HUMAN (+1)
372	Heterogeneous nuclear ribonucleoprotein H OS=Homo sapiens GN=HNRNPH1 PE=1 SV=4	sp|P31943|HNRH1_HUMAN (+2)
373	Isoform 2 of Coatomer subunit alpha OS=Homo sapiens GN=COPA	sp|P53621-2|COPA_HUMAN (+1)
374	26S protease regulatory subunit 7 OS=Homo sapiens GN=PSMC2 PE=1 SV=3	sp|P35998|PRS7_HUMAN
375	Actin-related protein 2/3 complex subunit 2 OS=Homo sapiens GN=ARPC2 PE=1 SV=1	sp|O15144|ARPC2_HUMAN
376	Calpain-2 catalytic subunit OS=Homo sapiens GN=CAPN2 PE=1 SV=6	sp|P17655|CAN2_HUMAN
377	Poly [ADP-ribose] polymerase 1 OS=Homo sapiens GN=PARP1 PE=1 SV=4	sp|P09874|PARP1_HUMAN
378	Inter-alpha-trypsin inhibitor heavy chain H2 OS=Homo sapiens GN=ITIH2 PE=1 SV=2	sp|P19823|ITIH2_HUMAN (+1)
379	Lupus La protein OS=Homo sapiens GN=SSB PE=1 SV=2	sp|P05455|LA_HUMAN
380	Dihydrolipoyllysine-residue succinyltransferase component of 2-oxoglutarate dehydrogenase complex, mitochondrial OS=Homo sapiens GN=DLST PE=1 SV=4	sp|P36957|ODO2_HUMAN

381	Collagen alpha-1(V) chain OS=Homo sapiens GN=COL5A1 PE=1 SV=3	sp|P20908|CO5A1_HUMAN
382	Plasma protease C1 inhibitor OS=Homo sapiens GN=SERPING1 PE=1 SV=2	sp|P05155|IC1_HUMAN (+2)
383	Protein kinase C delta-binding protein OS=Homo sapiens GN=PRKCDBP PE=1 SV=3	sp|Q969G5|PRDBP_HUMAN
384	Isoform Cytoplasmic of Fumarate hydratase, mitochondrial OS=Homo sapiens GN=FH	sp|P07954-2|FUMH_HUMAN (+1)
385	Isoform 2 of Nidogen-2 OS=Homo sapiens GN=NID2	sp|Q14112-2|NID2_HUMAN (+1)
386	Actin-related protein 2 OS=Homo sapiens GN=ACTR2 PE=1 SV=1	sp|P61160|ARP2_HUMAN
387	F-actin-capping protein subunit alpha-2 OS=Homo sapiens GN=CAPZA2 PE=1 SV=3	sp|P47755|CAZA2_HUMAN
388	Calpain-1 catalytic subunit OS=Homo sapiens GN=CAPN1 PE=1 SV=1	sp|P07384|CAN1_HUMAN
389	Isoform 2 of Interleukin enhancer-binding factor 3 OS=Homo sapiens GN=ILF3	sp|Q12906-2|ILF3_HUMAN (+6)
390	3-hydroxyacyl-CoA dehydrogenase type-2 OS=Homo sapiens GN=HSD17B10 PE=1 SV=3	sp|Q99714|HCD2_HUMAN
391	N(G),N(G)-dimethylarginine dimethylaminohydrolase 2 OS=Homo sapiens GN=DDAH2 PE=1 SV=1	sp|O95865|DDAH2_HUMAN
392	KDEL motif-containing protein 2 OS=Homo sapiens GN=KDELC2 PE=1 SV=2	sp|Q7Z4H8|KDEL2_HUMAN
393	Peptidyl-prolyl cis-trans isomerase A OS=Homo sapiens GN=PPIA PE=1 SV=2	sp|P62937|PPIA_HUMAN
394	Guanine nucleotide-binding protein G(I)/G(S)/G(T) subunit beta-1 OS=Homo sapiens GN=GNB1 PE=1 SV=3	sp|P62873|GBB1_HUMAN (+2)
395	Chloride intracellular channel protein 4 OS=Homo sapiens GN=CLIC4 PE=1 SV=4	sp|Q9Y696|CLIC4_HUMAN
396	Prostacyclin synthase OS=Homo sapiens GN=PTGIS PE=1 SV=1	sp|Q16647|PTGIS_HUMAN
397	Plasminogen OS=Homo sapiens GN=PLG PE=1 SV=2	sp|P00747|PLMN_HUMAN
398	ATP-dependent RNA helicase DDX1 OS=Homo sapiens GN=DDX1 PE=1 SV=2	sp|Q92499|DDX1_HUMAN
399	Phosphoglycerate mutase 1 OS=Homo sapiens GN=PGAM1 PE=1 SV=2	sp|P18669|PGAM1_HUMAN
400	Isoform Long of Proteasome subunit alpha type-1 OS=Homo sapiens GN=PSMA1	sp|P25786-2|PSA1_HUMAN (+1)
401	Interleukin enhancer-binding factor 2 OS=Homo sapiens GN=ILF2 PE=1 SV=2	sp|Q12905|ILF2_HUMAN
402	Alcohol dehydrogenase [NADP(+)] OS=Homo sapiens GN=AKR1A1 PE=1 SV=3	sp|P14550|AK1A1_HUMAN
403	Stathmin OS=Homo sapiens GN=STMN1 PE=1 SV=3	sp|P16949|STMN1_HUMAN
404	Chloride intracellular channel protein 1 OS=Homo sapiens GN=CLIC1 PE=1 SV=4	sp|O00299|CLIC1_HUMAN
405	Isoform B of Ras-related C3 botulinum toxin substrate 1 OS=Homo sapiens GN=RAC1	sp|P63000-2|RAC1_HUMAN (+1)
406	Gamma-enolase OS=Homo sapiens GN=ENO2 PE=1 SV=3	sp|P09104|ENOG_HUMAN (+1)
407	Valine--tRNA ligase OS=Homo sapiens GN=VARS PE=1 SV=4	sp|P26640|SYVC_HUMAN
408	Isoform 2 of Olfactomedin-like protein 3 OS=Homo sapiens GN=OLFML3	sp|Q9NRN5-2|OLFL3_HUMAN (+1)
409	D-3-phosphoglycerate dehydrogenase OS=Homo sapiens GN=PHGDH PE=1 SV=4	sp|O43175|SERA_HUMAN (+1)
410	Cleavage and polyadenylation-specificity factor subunit 6 OS=Homo sapiens GN=CPSF6 PE=2 SV=1	tr|F8WJN3|F8WJN3_HUMAN
411	ADP/ATP translocase 3 OS=Homo sapiens GN=SLC25A6 PE=1 SV=4	sp|P12236|ADT3_HUMAN

412	Isoform SM-B of Small nuclear ribonucleoprotein-associated proteins B and B' OS=Homo sapiens GN=SNRPB	sp|P14678-2|RSMB_HUMAN (+2)
413	Isoform 2 of Sulfhydryl oxidase 1 OS=Homo sapiens GN=QSOX1	sp|O00391-2|QSOX1_HUMAN (+1)
414	Isoform C of AP-1 complex subunit beta-1 OS=Homo sapiens GN=AP1B1	sp|Q10567-3|AP1B1_HUMAN (+4)
415	Isoform 2 of Clusterin OS=Homo sapiens GN=CLU	sp|P10909-2|CLUS_HUMAN (+3)
416	Transgelin-2 OS=Homo sapiens GN=TAGLN2 PE=1 SV=3	sp|P37802|TAGL2_HUMAN
417	Isoform 2 of Histone H1.0 OS=Homo sapiens GN=H1F0	sp|P07305-2|H10_HUMAN (+1)
418	PDZ and LIM domain protein 5 OS=Homo sapiens GN=PDLIM5 PE=1 SV=5	sp|Q96HC4|PDLI5_HUMAN
419	Isoform 2 of AP-2 complex subunit alpha-2 OS=Homo sapiens GN=AP2A2	sp|O94973-2|AP2A2_HUMAN (+1)
420	Importin-7 OS=Homo sapiens GN=IPO7 PE=1 SV=1	sp|O95373|IPO7_HUMAN
421	Vitronectin OS=Homo sapiens GN=VTN PE=1 SV=1	sp|P04004|VTNC_HUMAN
422	Dynein heavy chain 2, axonemal OS=Homo sapiens GN=DNAH2 PE=2 SV=3	sp|Q9P225|DYH2_HUMAN (+1)
423	6-phosphofructokinase type C OS=Homo sapiens GN=PFKP PE=1 SV=2	sp|Q01813|K6PP_HUMAN
424	Fatty acid synthase OS=Homo sapiens GN=FASN PE=1 SV=3	sp|P49327|FAS_HUMAN
425	Protein phosphatase 1 regulatory subunit 7 (Fragment) OS=Homo sapiens GN=PPP1R7 PE=2 SV=1	tr|C9J177|C9J177_HUMAN
426	Isoform 2 of Protein SET OS=Homo sapiens GN=SET	sp|Q01105-2|SET_HUMAN
427	T-complex protein 1 subunit theta OS=Homo sapiens GN=CCT8 PE=1 SV=4	sp|P50990|TCPQ_HUMAN (+2)
428	Plexin domain-containing protein 2 OS=Homo sapiens GN=PLXDC2 PE=1 SV=1	sp|Q6UX71|PXDC2_HUMAN
429	Isoform 3 of Selenium-binding protein 1 OS=Homo sapiens GN=SELENBP1	sp|Q13228-3|SBP1_HUMAN (+2)
430	Protein transport protein Sec23A OS=Homo sapiens GN=SEC23A PE=1 SV=2	sp|Q15436|SC23A_HUMAN (+3)
431	Isoform 2 of Collagen alpha-6(VI) chain OS=Homo sapiens GN=COL6A6	sp|A6NMZ7-2|CO6A6_HUMAN (+2)
432	Glutathione S-transferase Mu 3 OS=Homo sapiens GN=GSTM3 PE=1 SV=3	sp|P21266|GSTM3_HUMAN
433	Serpin B6 OS=Homo sapiens GN=SERPINB6 PE=1 SV=3	sp|P35237|SPB6_HUMAN
434	Isoform 8 of Tropomyosin alpha-1 chain OS=Homo sapiens GN=TPM1	sp|P09493-8|TPM1_HUMAN
435	Quinone oxidoreductase OS=Homo sapiens GN=CRYZ PE=1 SV=1	sp|Q08257|QOR_HUMAN
436	Isoform 2 of 26S proteasome non-ATPase regulatory subunit 5 OS=Homo sapiens GN=PSMD5	sp|Q16401-2|PSMD5_HUMAN (+1)
437	Isoform 2 of Cytosol aminopeptidase OS=Homo sapiens GN=LAP3	sp|P28838-2|AMPL_HUMAN (+1)
438	Proteasome subunit alpha type-6 OS=Homo sapiens GN=PSMA6 PE=1 SV=1	sp|P60900|PSA6_HUMAN (+2)
439	60S ribosomal protein L6 OS=Homo sapiens GN=RPL6 PE=1 SV=3	sp|Q02878|RL6_HUMAN
440	Alpha-1-acid glycoprotein 2 OS=Homo sapiens GN=ORM2 PE=1 SV=2	sp|P19652|A1AG2_HUMAN
441	Apolipoprotein A-II OS=Homo sapiens GN=APOA2 PE=1 SV=1	sp|P02652|APOA2_HUMAN
442	Cathepsin D OS=Homo sapiens GN=CTSD PE=1 SV=1	sp|P07339|CATD_HUMAN
443	Isoform 4 of Leukotriene A-4 hydrolase OS=Homo sapiens GN=LTA4H	sp|P09960-4|LKHA4_HUMAN (+1)

444	Citrate synthase, mitochondrial OS=Homo sapiens GN=CS PE=1 SV=2	sp|O75390|CISY_HUMAN (+1)
445	Aspartate--tRNA ligase, cytoplasmic OS=Homo sapiens GN=DARS PE=1 SV=2	sp|P14868|SYDC_HUMAN
446	Isoform 2 of T-complex protein 1 subunit delta OS=Homo sapiens GN=CCT4	sp|P50991-2|TCPD_HUMAN (+1)
447	Protein argonaute-1 OS=Homo sapiens GN=AGO1 PE=1 SV=3	sp|Q9UL18|AGO1_HUMAN (+1)
448	UDP-glucose 6-dehydrogenase OS=Homo sapiens GN=UGDH PE=1 SV=1	sp|O60701|UGDH_HUMAN (+1)
449	Isoform 1 of Ubiquitin-conjugating enzyme E2 variant 1 OS=Homo sapiens GN=UBE2V1	sp|Q13404-1|UB2V1_HUMAN (+4)
450	Isoleucine--tRNA ligase, cytoplasmic OS=Homo sapiens GN=IARS PE=1 SV=2	sp|P41252|SYIC_HUMAN (+1)
451	40S ribosomal protein S21 OS=Homo sapiens GN=RPS21 PE=1 SV=1	sp|P63220|RS21_HUMAN (+1)
452	Exportin-1 OS=Homo sapiens GN=XPO1 PE=1 SV=1	sp|O14980|XPO1_HUMAN
453	Isoform 4 of Leucine-rich repeat flightless-interacting protein 1 OS=Homo sapiens GN=LRRFIP1	sp|Q32MZ4-4|LRRF1_HUMAN (+1)
454	Isoform 2 of ATP-dependent RNA helicase DDX3X OS=Homo sapiens GN=DDX3X	sp|O00571-2|DDX3X_HUMAN (+1)
455	Heat shock 70 kDa protein 6 OS=Homo sapiens GN=HSPA6 PE=1 SV=2	sp|P17066|HSP76_HUMAN
456	Isoform 2 of Prohibitin-2 OS=Homo sapiens GN=PHB2	sp|Q99623-2|PHB2_HUMAN (+4)
457	Isoform 3 of Obg-like ATPase 1 OS=Homo sapiens GN=OLA1	sp|Q9NTK5-3|OLA1_HUMAN (+3)
458	Isoform 2 of Inactive tyrosine-protein kinase 7 OS=Homo sapiens GN=PTK7	sp|Q13308-2|PTK7_HUMAN (+4)
459	Bifunctional glutamate/proline--tRNA ligase OS=Homo sapiens GN=EPRS PE=1 SV=5	sp|P07814|SYEP_HUMAN
460	Soluble scavenger receptor cysteine-rich domain-containing protein SSC5D OS=Homo sapiens GN=SSC5D PE=2 SV=3	sp|A1L4H1|SRCRL_HUMAN
461	Protein farnesyltransferase/geranylgeranyltransferase type-1 subunit alpha OS=Homo sapiens GN=FNTA PE=1 SV=1	sp|P49354|FNTA_HUMAN
462	Isoform Alpha-6X2A of Integrin alpha-6 OS=Homo sapiens GN=ITGA6	sp|P23229-4|ITA6_HUMAN (+7)
463	Isoform 2 of Protein phosphatase 1 regulatory subunit 12A OS=Homo sapiens GN=PPP1R12A	sp|O14974-2|MYPT1_HUMAN (+5)
464	26S proteasome non-ATPase regulatory subunit 3 OS=Homo sapiens GN=PSMD3 PE=1 SV=2	sp|O43242|PSMD3_HUMAN
465	Coiled-coil domain-containing protein 6 OS=Homo sapiens GN=CCDC6 PE=1 SV=2	sp|Q16204|CCDC6_HUMAN
466	Aldehyde dehydrogenase X, mitochondrial OS=Homo sapiens GN=ALDH1B1 PE=1 SV=3	sp|P30837|AL1B1_HUMAN
467	Isoform 3 of Nucleophosmin OS=Homo sapiens GN=NPM1	sp|P06748-3|NPM_HUMAN (+1)
468	Kinesin-1 heavy chain OS=Homo sapiens GN=KIF5B PE=1 SV=1	sp|P33176|KINH_HUMAN
469	Thioredoxin-dependent peroxide reductase, mitochondrial OS=Homo sapiens GN=PRDX3 PE=1 SV=3	sp|P30048|PRDX3_HUMAN (+1)
470	Alpha-2-HS-glycoprotein OS=Homo sapiens GN=AHSG PE=1 SV=1	sp|P02765|FETUA_HUMAN
471	Isoform 2 of Basigin OS=Homo sapiens GN=BSG	sp|P35613-2|BASI_HUMAN (+3)
472	cAMP-dependent protein kinase type II-alpha regulatory subunit OS=Homo sapiens GN=PRKAR2A PE=1 SV=2	sp|P13861|KAP2_HUMAN (+1)

473	Isoform 2 of 72 kDa type IV collagenase OS=Homo sapiens GN=MMP2	sp|P08253-2|MMP2_HUMAN (+2)
474	Keratin, type II cytoskeletal 5 OS=Homo sapiens GN=KRT5 PE=1 SV=3	sp|P13647|K2C5_HUMAN
475	Isoform 2 of Mitogen-activated protein kinase 1 OS=Homo sapiens GN=MAPK1	sp|P28482-2|MK01_HUMAN (+1)
476	Isoform 2 of Nck-associated protein 1 OS=Homo sapiens GN=NCKAP1	sp|Q9Y2A7-2|NCKP1_HUMAN (+1)
477	Dynein light chain 1, cytoplasmic OS=Homo sapiens GN=DYNLL1 PE=1 SV=1	sp|P63167|DYL1_HUMAN
478	Isoform 2 of KH domain-containing, RNA-binding, signal transduction-associated protein 1 OS=Homo sapiens GN=KHDRBS1	sp|Q07666-2|KHDR1_HUMAN (+1)
479	26S protease regulatory subunit 6A OS=Homo sapiens GN=PSMC3 PE=1 SV=3	sp|P17980|PRS6A_HUMAN (+2)
480	Coatomer subunit delta OS=Homo sapiens GN=ARCN1 PE=1 SV=1	sp|P48444|COPD_HUMAN (+1)
481	Ubiquitin-like modifier-activating enzyme 6 OS=Homo sapiens GN=UBA6 PE=1 SV=1	sp|A0AVT1|UBA6_HUMAN
482	Dolichyl-diphosphooligosaccharide--protein glycosyltransferase 48 kDa subunit OS=Homo sapiens GN=DDOST PE=1 SV=4	sp|P39656|OST48_HUMAN
483	Heterogeneous nuclear ribonucleoprotein A3 OS=Homo sapiens GN=HNRNPA3 PE=1 SV=2	sp|P51991|ROA3_HUMAN
484	Cytoskeleton-associated protein 4 OS=Homo sapiens GN=CKAP4 PE=1 SV=2	sp|Q07065|CKAP4_HUMAN
485	Isoform 2 of Nexilin OS=Homo sapiens GN=NEXN	sp|Q0ZGT2-2|NEXN_HUMAN (+3)
486	Isoform 3 of Basic leucine zipper and W2 domain-containing protein 1 OS=Homo sapiens GN=BZW1	sp|Q7L1Q6-3|BZW1_HUMAN (+2)
487	Keratin, type II cytoskeletal 6A OS=Homo sapiens GN=KRT6A PE=1 SV=3	sp|P02538|K2C6A_HUMAN (+1)
488	Isoform 2 of Mitotic checkpoint protein BUB3 OS=Homo sapiens GN=BUB3	sp|O43684-2|BUB3_HUMAN (+1)
489	60S ribosomal protein L3 OS=Homo sapiens GN=RPL3 PE=1 SV=2	sp|P39023|RL3_HUMAN (+1)
490	T-complex protein 1 subunit epsilon OS=Homo sapiens GN=CCT5 PE=1 SV=1	sp|P48643|TCPE_HUMAN (+4)
491	Protein FAM49B OS=Homo sapiens GN=FAM49B PE=1 SV=1	sp|Q9NUQ9|FA49B_HUMAN
492	40S ribosomal protein S10 OS=Homo sapiens GN=RPS10 PE=1 SV=1	sp|P46783|RS10_HUMAN (+2)
493	Isoform 2 of Argininosuccinate lyase OS=Homo sapiens GN=ASL	sp|P04424-2|ARLY_HUMAN (+2)
494	Laminin subunit alpha-1 OS=Homo sapiens GN=LAMA1 PE=1 SV=2	sp|P25391|LAMA1_HUMAN
495	Retinal dehydrogenase 1 OS=Homo sapiens GN=ALDH1A1 PE=1 SV=2	sp|P00352|AL1A1_HUMAN
496	Leucine-rich alpha-2-glycoprotein OS=Homo sapiens GN=LRG1 PE=1 SV=2	sp|P02750|A2GL_HUMAN
497	Isoform Cytoplasmic+peroxisomal of Peroxiredoxin-5, mitochondrial OS=Homo sapiens GN=PRDX5	sp|P30044-2|PRDX5_HUMAN (+1)
498	Proliferation-associated protein 2G4 OS=Homo sapiens GN=PA2G4 PE=1 SV=3	sp|Q9UQ80|PA2G4_HUMAN
499	Carbonyl reductase [NADPH] 1 OS=Homo sapiens GN=CBR1 PE=1 SV=3	sp|P16152|CBR1_HUMAN
500	40S ribosomal protein S3a OS=Homo sapiens GN=RPS3A PE=1 SV=2	sp|P61247|RS3A_HUMAN (+6)
501	Isoform 1 of Voltage-dependent anion-selective channel protein 2 OS=Homo sapiens GN=VDAC2	sp|P45880-1|VDAC2_HUMAN (+2)

502	Isoform 2 of Cytoplasmic FMR1-interacting protein 1 OS=Homo sapiens GN=CYFIP1	sp|Q7L576-2|CYFP1_HUMAN (+1)
503	Aspartyl aminopeptidase OS=Homo sapiens GN=DNPEP PE=1 SV=1	sp|Q9ULA0|DNPEP_HUMAN (+1)
504	40S ribosomal protein S4, X isoform OS=Homo sapiens GN=RPS4X PE=1 SV=2	sp|P62701|RS4X_HUMAN
505	Adenosylhomocysteinase OS=Homo sapiens GN=AHCY PE=1 SV=4	sp|P23526|SAHH_HUMAN
506	Calpain small subunit 1 OS=Homo sapiens GN=CAPNS1 PE=1 SV=1	sp|P04632|CPNS1_HUMAN (+1)
507	Serine/arginine-rich splicing factor 1 OS=Homo sapiens GN=SRSF1 PE=1 SV=2	sp|Q07955|SRSF1_HUMAN (+1)
508	Proteasome subunit alpha type-5 OS=Homo sapiens GN=PSMA5 PE=1 SV=3	sp|P28066|PSA5_HUMAN
509	Isoform 3 of Ubiquitin-conjugating enzyme E2 L3 OS=Homo sapiens GN=UBE2L3	sp|P68036-3|UB2L3_HUMAN (+1)
510	Isoform 2 of Prothymosin alpha OS=Homo sapiens GN=PTMA	sp|P06454-2|PTMA_HUMAN (+5)
511	Carboxypeptidase Q OS=Homo sapiens GN=CPQ PE=1 SV=1	sp|Q9Y646|CBPQ_HUMAN
512	6-phosphogluconate dehydrogenase, decarboxylating OS=Homo sapiens GN=PGD PE=1 SV=3	sp|P52209|6PGD_HUMAN (+1)
513	Barrier-to-autointegration factor OS=Homo sapiens GN=BANF1 PE=1 SV=1	sp|O75531|BAF_HUMAN
514	Rho GDP-dissociation inhibitor 1 OS=Homo sapiens GN=ARHGDIA PE=1 SV=3	sp|P52565|GDIR1_HUMAN (+2)
515	Protein S100-A11 OS=Homo sapiens GN=S100A11 PE=1 SV=2	sp|P31949|S10AB_HUMAN
516	Cytochrome b-c1 complex subunit 2, mitochondrial OS=Homo sapiens GN=UQCRC2 PE=1 SV=3	sp|P22695|QCR2_HUMAN (+2)
517	Corticosteroid-binding globulin OS=Homo sapiens GN=SERPINA6 PE=1 SV=1	sp|P08185|CBG_HUMAN
518	Isoform 2 of Sarcolemmal membrane-associated protein OS=Homo sapiens GN=SLMAP	sp|Q14BN4-2|SLMAP_HUMAN (+2)
519	4-trimethylaminobutyraldehyde dehydrogenase OS=Homo sapiens GN=ALDH9A1 PE=1 SV=3	sp|P49189|AL9A1_HUMAN
520	Major vault protein OS=Homo sapiens GN=MVP PE=1 SV=4	sp|Q14764|MVP_HUMAN
521	Isoform MLC3 of Myosin light chain 1/3, skeletal muscle isoform OS=Homo sapiens GN=MYL1	sp|P05976-2|MYL1_HUMAN
522	Transmembrane emp24 domain-containing protein 2 OS=Homo sapiens GN=TMED2 PE=1 SV=1	sp|Q15363|TMED2_HUMAN (+1)
523	Isoform 2 of Transcription intermediary factor 1-beta OS=Homo sapiens GN=TRIM28	sp|Q13263-2|TIF1B_HUMAN (+2)
524	Ribonuclease inhibitor OS=Homo sapiens GN=RNH1 PE=1 SV=2	sp|P13489|RINI_HUMAN
525	Isoform 2 of LIM and senescent cell antigen-like-containing domain protein 2 OS=Homo sapiens GN=LIMS2	sp|Q7Z4I7-2|LIMS2_HUMAN (+4)
526	Isoform 3 of H/ACA ribonucleoprotein complex subunit 4 OS=Homo sapiens GN=DKC1	sp|O60832-2|DKC1_HUMAN (+3)
527	Fatty acid-binding protein, epidermal OS=Homo sapiens GN=FABP5 PE=1 SV=3	sp|Q01469|FABP5_HUMAN
528	Copine-1 OS=Homo sapiens GN=CPNE1 PE=1 SV=1	sp|Q99829|CPNE1_HUMAN (+3)
529	Tight junction protein 1 (Zona occludens 1), isoform CRA_a OS=Homo sapiens GN=TJP1 PE=2 SV=1	tr|G3V1L9|G3V1L9_HUMAN (+3)
530	Isoform 2 of Phosphatidylinositol-binding clathrin assembly protein OS=Homo sapiens GN=PICALM	sp|Q13492-2|PICAL_HUMAN (+3)
531	Isoform 2 of Xaa-Pro aminopeptidase 1 OS=Homo sapiens GN=XPNPEP1	sp|Q9NQW7-2|XPP1_HUMAN (+4)

532	CD81 antigen (Fragment) OS=Homo sapiens GN=CD81 PE=4 SV=1	tr|H0YDL9|H0YDL9_HUMAN
533	Heterogeneous nuclear ribonucleoprotein A1-like 2 OS=Homo sapiens GN=HNRNPA1L2 PE=2 SV=2	sp|Q32P51|RA1L2_HUMAN
534	Histidine-rich glycoprotein OS=Homo sapiens GN=HRG PE=1 SV=1	sp|P04196|HRG_HUMAN
535	Target of Nesh-SH3 OS=Homo sapiens GN=ABI3BP PE=1 SV=1	sp|Q7Z7G0|TARSH_HUMAN (+1)
536	Isoform 2 of Splicing factor U2AF 65 kDa subunit OS=Homo sapiens GN=U2AF2	sp|P26368-2|U2AF2_HUMAN (+2)
537	Complement component C7 OS=Homo sapiens GN=C7 PE=1 SV=2	sp|P10643|CO7_HUMAN
538	Isoform 2 of Niban-like protein 1 OS=Homo sapiens GN=FAM129B	sp|Q96TA1-2|NIBL1_HUMAN (+1)
539	Histone deacetylase 2 OS=Homo sapiens GN=HDAC2 PE=2 SV=1	tr|J3KPW7|J3KPW7_HUMAN
540	Isoform 4 of Cadherin-13 OS=Homo sapiens GN=CDH13	sp|P55290-4|CAD13_HUMAN (+1)
541	Alpha-2-antiplasmin OS=Homo sapiens GN=SERPINF2 PE=1 SV=3	sp|P08697|A2AP_HUMAN (+1)
542	Isoform 2 of COP9 signalosome complex subunit 3 OS=Homo sapiens GN=COPS3	sp|Q9UNS2-2|CSN3_HUMAN (+1)
543	Protein NDRG1 OS=Homo sapiens GN=NDRG1 PE=1 SV=1	sp|Q92597|NDRG1_HUMAN
544	Isoform Gnas-2 of Guanine nucleotide-binding protein G(s) subunit alpha isoforms short OS=Homo sapiens GN=GNAS	sp|P63092-2|GNAS2_HUMAN (+5)
545	Isoform 2 of Septin-11 OS=Homo sapiens GN=SEPT11	sp|Q9NVA2-2|SEP11_HUMAN (+3)
546	Immunoglobulin superfamily containing leucine-rich repeat protein OS=Homo sapiens GN=ISLR PE=1 SV=1	sp|O14498|ISLR_HUMAN
547	40S ribosomal protein S26 OS=Homo sapiens GN=RPS26 PE=1 SV=3	sp|P62854|RS26_HUMAN (+1)
548	Isoform 2 of Actin-related protein 2/3 complex subunit 5 OS=Homo sapiens GN=ARPC5	sp|O15511-2|ARPC5_HUMAN (+2)
549	HLA class I histocompatibility antigen, A-2 alpha chain OS=Homo sapiens GN=HLA-A PE=1 SV=1	sp|P01892|1A02_HUMAN (+1)
550	Elongation factor 1-beta OS=Homo sapiens GN=EEF1B2 PE=1 SV=3	sp|P24534|EF1B_HUMAN
551	Isoform 2 of Secernin-1 OS=Homo sapiens GN=SCRN1	sp|Q12765-2|SCRN1_HUMAN (+1)
552	Tumor necrosis factor alpha-induced protein 8-like protein 3 OS=Homo sapiens GN=TNFAIP8L3 PE=2 SV=1	sp|Q5GJ75|TP8L3_HUMAN
553	Nucleoredoxin OS=Homo sapiens GN=NXN PE=1 SV=2	sp|Q6DKJ4|NXN_HUMAN
554	Acylamino-acid-releasing enzyme OS=Homo sapiens GN=APEH PE=2 SV=1	tr|C9JIF9|C9JIF9_HUMAN
555	Isoform 2 of NADH-ubiquinone oxidoreductase 75 kDa subunit, mitochondrial OS=Homo sapiens GN=NDUFS1	sp|P28331-2|NDUS1_HUMAN (+5)
556	Destrin OS=Homo sapiens GN=DSTN PE=1 SV=3	sp|P60981|DEST_HUMAN
557	Nucleoprotein TPR OS=Homo sapiens GN=TPR PE=1 SV=3	sp|P12270|TPR_HUMAN
558	Superoxide dismutase [Cu-Zn] OS=Homo sapiens GN=SOD1 PE=1 SV=2	sp|P00441|SODC_HUMAN (+1)
559	Glutamine--tRNA ligase OS=Homo sapiens GN=QARS PE=1 SV=1	sp|P47897|SYQ_HUMAN (+1)

560	Isoform 2 of Synemin OS=Homo sapiens GN=SYNM	sp|O15061-2|SYNEM_HUMAN (+3)
561	26S proteasome non-ATPase regulatory subunit 6 OS=Homo sapiens GN=PSMD6 PE=1 SV=1	sp|Q15008|PSMD6_HUMAN (+1)
562	Transmembrane protein 43 OS=Homo sapiens GN=TMEM43 PE=1 SV=1	sp|Q9BTV4|TMM43_HUMAN
563	Isoform 2 of Pyridoxal kinase OS=Homo sapiens GN=PDXK	sp|O00764-2|PDXK_HUMAN (+2)
564	COP9 signalosome complex subunit 5 OS=Homo sapiens GN=COPS5 PE=1 SV=4	sp|Q92905|CSN5_HUMAN
565	Isoform 2 of 40S ribosomal protein S3 OS=Homo sapiens GN=RPS3	sp|P23396-2|RS3_HUMAN (+2)
566	Isoform 2 of Heterogeneous nuclear ribonucleoprotein H3 OS=Homo sapiens GN=HNRNPH3	sp|P31942-2|HNRH3_HUMAN (+2)
567	Phosphatidylethanolamine-binding protein 1 OS=Homo sapiens GN=PEBP1 PE=1 SV=3	sp|P30086|PEBP1_HUMAN
568	LIM and cysteine-rich domains protein 1 OS=Homo sapiens GN=LMCD1 PE=1 SV=1	sp|Q9NZU5|LMCD1_HUMAN (+1)
569	Carbonic anhydrase 3 OS=Homo sapiens GN=CA3 PE=1 SV=3	sp|P07451|CAH3_HUMAN
570	V-type proton ATPase subunit B, brain isoform OS=Homo sapiens GN=ATP6V1B2 PE=1 SV=3	sp|P21281|VATB2_HUMAN
571	Isoform 2 of Protein arginine N-methyltransferase 5 OS=Homo sapiens GN=PRMT5	sp|O14744-2|ANM5_HUMAN (+4)
572	Isoform 2 of Protein arginine N-methyltransferase 1 OS=Homo sapiens GN=PRMT1	sp|Q99873-2|ANM1_HUMAN (+7)
573	Beta-2-syntrophin OS=Homo sapiens GN=SNTB2 PE=1 SV=1	sp|Q13425|SNTB2_HUMAN
574	Glycine--tRNA ligase OS=Homo sapiens GN=GARS PE=1 SV=3	sp|P41250|SYG_HUMAN
575	Isoform 2 of Xaa-Pro dipeptidase OS=Homo sapiens GN=PEPD	sp|P12955-2|PEPD_HUMAN (+1)
576	Isoform 2 of Actin-like protein 6A OS=Homo sapiens GN=ACTL6A	sp|O96019-2|ACL6A_HUMAN (+1)
577	Glucose 1,6-bisphosphate synthase OS=Homo sapiens GN=PGM2L1 PE=1 SV=3	sp|Q6PCE3|PGM2L_HUMAN
578	Macrophage-capping protein OS=Homo sapiens GN=CAPG PE=1 SV=2	sp|P40121|CAPG_HUMAN (+1)
579	Putative pre-mRNA-splicing factor ATP-dependent RNA helicase DHX15 OS=Homo sapiens GN=DHX15 PE=1 SV=2	sp|O43143|DHX15_HUMAN
580	Coatomer subunit beta' OS=Homo sapiens GN=COPB2 PE=1 SV=2	sp|P35606|COPB2_HUMAN (+1)
581	Isoform 2 of CAP-Gly domain-containing linker protein 1 OS=Homo sapiens GN=CLIP1	sp|P30622-1|CLIP1_HUMAN (+2)
582	26S protease regulatory subunit 4 OS=Homo sapiens GN=PSMC1 PE=1 SV=1	sp|P62191|PRS4_HUMAN (+1)
583	Peptidyl-prolyl cis-trans isomerase FKBP4 OS=Homo sapiens GN=FKBP4 PE=1 SV=3	sp|Q02790|FKBP4_HUMAN
584	Aldose reductase OS=Homo sapiens GN=AKR1B1 PE=1 SV=3	sp|P15121|ALDR_HUMAN
585	Chromobox protein homolog 1 OS=Homo sapiens GN=CBX1 PE=1 SV=1	sp|P83916|CBX1_HUMAN (+2)
586	Isoform 2 of Cullin-2 OS=Homo sapiens GN=CUL2	sp|Q13617-2|CUL2_HUMAN (+2)
587	NHL repeat-containing protein 2 OS=Homo sapiens GN=NHLRC2 PE=1 SV=1	sp|Q8NBF2|NHLC2_HUMAN
588	Interferon-induced guanylate-binding protein 1 OS=Homo sapiens GN=GBP1 PE=1 SV=2	sp|P32455|GBP1_HUMAN
589	Ubiquitin carboxyl-terminal hydrolase 7 OS=Homo sapiens GN=USP7 PE=1 SV=2	sp|Q93009|UBP7_HUMAN (+3)
590	Serine/threonine-protein phosphatase 2A catalytic subunit beta isoform OS=Homo sapiens GN=PPP2CB PE=1 SV=1	sp|P62714|PP2AB_HUMAN (+2)

591	Isoform 3 of Retinal dehydrogenase 2 OS=Homo sapiens GN=ALDH1A2	sp|O94788-3|AL1A2_HUMAN (+3)
592	60S ribosomal protein L12 OS=Homo sapiens GN=RPL12 PE=1 SV=1	sp|P30050|RL12_HUMAN
593	Prostaglandin reductase 1 OS=Homo sapiens GN=PTGR1 PE=1 SV=2	sp|Q14914|PTGR1_HUMAN
594	Zinc-alpha-2-glycoprotein OS=Homo sapiens GN=AZGP1 PE=1 SV=2	sp|P25311|ZA2G_HUMAN
595	Alpha-soluble NSF attachment protein OS=Homo sapiens GN=NAPA PE=1 SV=3	sp|P54920|SNAA_HUMAN (+2)
596	Isoform 2 of 26S protease regulatory subunit 8 OS=Homo sapiens GN=PSMC5	sp|P62195-2|PRS8_HUMAN (+1)
597	60S acidic ribosomal protein P2 OS=Homo sapiens GN=RPLP2 PE=1 SV=1	sp|P05387|RLA2_HUMAN
598	Succinate dehydrogenase [ubiquinone] flavoprotein subunit, mitochondrial OS=Homo sapiens GN=SDHA PE=1 SV=2	sp|P31040|DHSA_HUMAN (+2)
599	Isoform PBX1b of Pre-B-cell leukemia transcription factor 1 OS=Homo sapiens GN=PBX1	sp|P40424-2|PBX1_HUMAN (+4)
600	Isoform 2 of Ras-related protein Rab-5C OS=Homo sapiens GN=RAB5C	sp|P51148-2|RAB5C_HUMAN (+1)
601	60S ribosomal protein L30 OS=Homo sapiens GN=RPL30 PE=1 SV=2	sp|P62888|RL30_HUMAN
602	Tropomyosin alpha-4 chain OS=Homo sapiens GN=TPM4 PE=1 SV=3	sp|P67936|TPM4_HUMAN
603	ADP-ribosylation factor 5 OS=Homo sapiens GN=ARF5 PE=1 SV=2	sp|P84085|ARF5_HUMAN (+1)
604	Alpha/beta hydrolase domain-containing protein 14B OS=Homo sapiens GN=ABHD14B PE=1 SV=1	sp|Q96IU4|ABHEB_HUMAN (+2)
605	Junctophilin-2 OS=Homo sapiens GN=JPH2 PE=1 SV=2	sp|Q9BR39|JPH2_HUMAN
606	Isoform 2 of E3 ubiquitin-protein ligase CHIP OS=Homo sapiens GN=STUB1	sp|Q9UNE7-2|CHIP_HUMAN (+3)
607	Ig kappa chain V-III region WOL OS=Homo sapiens PE=1 SV=1	sp|P01623|KV305_HUMAN (+1)
608	Transthyretin OS=Homo sapiens GN=TTR PE=1 SV=1	sp|P02766|TTHY_HUMAN
609	Isoform 2 of Electron transfer flavoprotein subunit alpha, mitochondrial OS=Homo sapiens GN=ETFA	sp|P13804-2|ETFA_HUMAN (+2)
610	S-phase kinase-associated protein 1 OS=Homo sapiens GN=SKP1 PE=1 SV=2	sp|P63208|SKP1_HUMAN (+1)
611	Core histone macro-H2A.2 OS=Homo sapiens GN=H2AFY2 PE=1 SV=3	sp|Q9P0M6|H2AW_HUMAN
612	Heme-binding protein 2 OS=Homo sapiens GN=HEBP2 PE=1 SV=1	sp|Q9Y5Z4|HEBP2_HUMAN
613	Importin subunit alpha-4 OS=Homo sapiens GN=KPNA3 PE=1 SV=2	sp|O00505|IMA4_HUMAN
614	Isoform 2 of Thymidine phosphorylase OS=Homo sapiens GN=TYMP	sp|P19971-2|TYPH_HUMAN (+2)
615	Isoform 2 of Glutathione synthetase OS=Homo sapiens GN=GSS	sp|P48637-2|GSHB_HUMAN (+2)
616	Eukaryotic translation initiation factor 3 subunit G OS=Homo sapiens GN=EIF3G PE=1 SV=2	sp|O75821|EIF3G_HUMAN (+3)
617	Isoform 2 of 26S proteasome non-ATPase regulatory subunit 13 OS=Homo sapiens GN=PSMD13	sp|Q9UNM6-2|PSD13_HUMAN (+1)
618	Histone-binding protein RBBP7 OS=Homo sapiens GN=RBBP7 PE=1 SV=1	sp|Q16576|RBBP7_HUMAN (+1)
619	Aflatoxin B1 aldehyde reductase member 2 OS=Homo sapiens GN=AKR7A2 PE=1 SV=3	sp|O43488|ARK72_HUMAN (+1)
620	Vacuolar protein sorting-associated protein 26A OS=Homo sapiens GN=VPS26A PE=1 SV=2	sp|O75436|VP26A_HUMAN (+2)

621	Alpha-N-acetylgalactosaminidase OS=Homo sapiens GN=NAGA PE=1 SV=2	sp|P17050|NAGAB_HUMAN
622	Isoform 2 of Multifunctional protein ADE2 OS=Homo sapiens GN=PAICS	sp|P22234-2|PUR6_HUMAN (+1)
623	Probable ATP-dependent RNA helicase DDX6 OS=Homo sapiens GN=DDX6 PE=1 SV=2	sp|P26196|DDX6_HUMAN
624	Aldo-keto reductase family 1 member C3 OS=Homo sapiens GN=AKR1C3 PE=1 SV=4	sp|P42330|AK1C3_HUMAN (+2)
625	Isoform 3 of Exportin-2 OS=Homo sapiens GN=CSE1L	sp|P55060-3|XPO2_HUMAN (+2)
626	Eukaryotic translation initiation factor 3 subunit I OS=Homo sapiens GN=EIF3I PE=1 SV=1	sp|Q13347|EIF3I_HUMAN
627	Isoform 2 of Coiled-coil domain-containing protein 80 OS=Homo sapiens GN=CCDC80	sp|Q76M96-2|CCD80_HUMAN (+1)
628	Isoform 2 of COP9 signalosome complex subunit 4 OS=Homo sapiens GN=COPS4	sp|Q9BT78-2|CSN4_HUMAN (+3)
629	26S proteasome non-ATPase regulatory subunit 7 OS=Homo sapiens GN=PSMD7 PE=1 SV=2	sp|P51665|PSMD7_HUMAN
630	Serum amyloid P-component OS=Homo sapiens GN=APCS PE=1 SV=2	sp|P02743|SAMP_HUMAN
631	Isoform 2 of SWI/SNF complex subunit SMARCC2 OS=Homo sapiens GN=SMARCC2	sp|Q8TAQ2-2|SMRC2_HUMAN (+3)
632	Matrix-remodeling-associated protein 5 OS=Homo sapiens GN=MXRA5 PE=2 SV=3	sp|Q9NR99|MXRA5_HUMAN
633	Isoform 10 of CD44 antigen OS=Homo sapiens GN=CD44	sp|P16070-10|CD44_HUMAN (+21)
634	Serine/threonine-protein kinase MST4 OS=Homo sapiens GN=MST4 PE=1 SV=2	sp|Q9P289|MST4_HUMAN (+3)
635	Isoform 5 of Sex hormone-binding globulin OS=Homo sapiens GN=SHBG	sp|P04278-5|SHBG_HUMAN (+2)
636	Beta-centractin OS=Homo sapiens GN=ACTR1B PE=1 SV=1	sp|P42025|ACTY_HUMAN
637	tRNA-splicing ligase RtcB homolog OS=Homo sapiens GN=RTCB PE=1 SV=1	sp|Q9Y3I0|RTCB_HUMAN
638	Complement component C8 alpha chain OS=Homo sapiens GN=C8A PE=1 SV=2	sp|P07357|CO8A_HUMAN
639	Pre-mRNA-processing factor 19 OS=Homo sapiens GN=PRPF19 PE=1 SV=1	sp|Q9UMS4|PRP19_HUMAN
640	Epidermal growth factor receptor kinase substrate 8 OS=Homo sapiens GN=EPS8 PE=1 SV=1	sp|Q12929|EPS8_HUMAN
641	Neurocalcin-delta OS=Homo sapiens GN=NCALD PE=2 SV=2	sp|P61601|NCALD_HUMAN (+1)
642	Isoform 3 of Aldehyde dehydrogenase family 16 member A1 OS=Homo sapiens GN=ALDH16A1	sp|Q8IZ83-3|A16A1_HUMAN (+3)
643	Alpha-N-acetylglucosaminidase OS=Homo sapiens GN=NAGLU PE=1 SV=2	sp|P54802|ANAG_HUMAN
644	Isoform 2 of UDP-glucose:glycoprotein glucosyltransferase 1 OS=Homo sapiens GN=UGGT1	sp|Q9NYU2-2|UGGG1_HUMAN (+1)
645	Complement C1s subcomponent OS=Homo sapiens GN=C1S PE=1 SV=1	sp|P09871|C1S_HUMAN (+1)
646	Isoform Beta of Signal transducer and activator of transcription 1-alpha/beta OS=Homo sapiens GN=STAT1	sp|P42224-2|STAT1_HUMAN (+2)
647	Isoform 2 of Sarcoplasmic/endoplasmic reticulum calcium ATPase 2 OS=Homo sapiens GN=ATP2A2	sp|P16615-2|AT2A2_HUMAN (+5)
648	Pre-mRNA branch site protein p14 OS=Homo sapiens GN=SF3B14 PE=1 SV=1	sp|Q9Y3B4|PM14_HUMAN
649	Keratin, type I cytoskeletal 16 OS=Homo sapiens GN=KRT16 PE=1 SV=4	sp|P08779|K1C16_HUMAN
650	Isoform 2 of Heterochromatin protein 1-binding protein 3 OS=Homo sapiens GN=HP1BP3	sp|Q5SSJ5-2|HP1B3_HUMAN (+3)

651	Isoform 2 of Calmodulin-regulated spectrin-associated protein 2 OS=Homo sapiens GN=CAMSAP2	sp|Q08AD1-2|CAMP2_HUMAN (+2)
652	40S ribosomal protein S2 OS=Homo sapiens GN=RPS2 PE=1 SV=2	sp|P15880|RS2_HUMAN (+2)
653	Isoform 2 of Sushi repeat-containing protein SRPX OS=Homo sapiens GN=SRPX	sp|P78539-2|SRPX_HUMAN (+3)
654	EF-hand domain-containing protein D1 OS=Homo sapiens GN=EFHD1 PE=1 SV=1	sp|Q9BUP0|EFHD1_HUMAN
655	Tripeptidyl-peptidase 1 OS=Homo sapiens GN=TPP1 PE=1 SV=2	sp|O14773|TPP1_HUMAN
656	Actin-related protein 2/3 complex subunit 1B OS=Homo sapiens GN=ARPC1B PE=1 SV=3	sp|O15143|ARC1B_HUMAN
657	Isoform 2 of Eukaryotic translation initiation factor 3 subunit L OS=Homo sapiens GN=EIF3L	sp|Q9Y262-2|EIF3L_HUMAN (+2)
658	Pyruvate dehydrogenase phosphatase regulatory subunit, mitochondrial OS=Homo sapiens GN=PDPR PE=1 SV=2	sp|Q8NCN5|PDPR_HUMAN (+1)
659	Nodal modulator 1 OS=Homo sapiens GN=NOMO1 PE=1 SV=5	sp|Q15155|NOMO1_HUMAN
660	Isoform Long of Glucose-6-phosphate 1-dehydrogenase OS=Homo sapiens GN=G6PD	sp|P11413-2|G6PD_HUMAN (+5)
661	Dolichyl-diphosphooligosaccharide--protein glycosyltransferase subunit 1 OS=Homo sapiens GN=RPN1 PE=1 SV=1	sp|P04843|RPN1_HUMAN
662	PDZ and LIM domain protein 1 OS=Homo sapiens GN=PDLIM1 PE=1 SV=4	sp|O00151|PDLI1_HUMAN
663	Cystatin-B OS=Homo sapiens GN=CSTB PE=1 SV=2	sp|P04080|CYTB_HUMAN
664	Ectonucleotide pyrophosphatase/phosphodiesterase family member 1 OS=Homo sapiens GN=ENPP1 PE=1 SV=2	sp|P22413|ENPP1_HUMAN
665	UMP-CMP kinase OS=Homo sapiens GN=CMPK1 PE=1 SV=3	sp|P30085|KCY_HUMAN
666	Peroxiredoxin-2 OS=Homo sapiens GN=PRDX2 PE=1 SV=5	sp|P32119|PRDX2_HUMAN
667	14-3-3 protein eta OS=Homo sapiens GN=YWHAH PE=1 SV=4	sp|Q04917|1433F_HUMAN
668	Isoform 2 of Nuclear pore complex protein Nup93 OS=Homo sapiens GN=NUP93	sp|Q8N1F7-2|NUP93_HUMAN (+3)
669	Cytosolic non-specific dipeptidase OS=Homo sapiens GN=CNDP2 PE=1 SV=2	sp|Q96KP4|CNDP2_HUMAN
670	Laminin subunit gamma-3 OS=Homo sapiens GN=LAMC3 PE=1 SV=3	sp|Q9Y6N6|LAMC3_HUMAN (+1)
671	COP9 signalosome complex subunit 2 OS=Homo sapiens GN=COPS2 PE=2 SV=1	tr|B4DIH5|B4DIH5_HUMAN
672	Glutathione S-transferase Mu 2 OS=Homo sapiens GN=GSTM2 PE=1 SV=2	sp|P28161|GSTM2_HUMAN (+1)
673	Isoform 5 of Tropomyosin alpha-3 chain OS=Homo sapiens GN=TPM3	sp|P06753-5|TPM3_HUMAN
674	Spermine synthase OS=Homo sapiens GN=SMS PE=1 SV=2	sp|P52788|SPSY_HUMAN (+2)
675	Lysosome membrane protein 2 OS=Homo sapiens GN=SCARB2 PE=1 SV=2	sp|Q14108|SCRB2_HUMAN
676	ATP-dependent DNA helicase Q1 OS=Homo sapiens GN=RECQL PE=1 SV=3	sp|P46063|RECQ1_HUMAN
677	Adenine phosphoribosyltransferase OS=Homo sapiens GN=APRT PE=1 SV=2	sp|P07741|APT_HUMAN (+1)
678	Isoform Short of Endoglin OS=Homo sapiens GN=ENG	sp|P17813-2|EGLN_HUMAN (+1)

679	Platelet-activating factor acetylhydrolase IB subunit alpha OS=Homo sapiens GN=PAFAH1B1 PE=1 SV=2	sp|P43034|LIS1_HUMAN
680	40S ribosomal protein S9 OS=Homo sapiens GN=RPS9 PE=1 SV=3	sp|P46781|RS9_HUMAN
681	Serine--tRNA ligase, cytoplasmic OS=Homo sapiens GN=SARS PE=1 SV=3	sp|P49591|SYSC_HUMAN (+1)
682	Transforming protein RhoA OS=Homo sapiens GN=RHOA PE=1 SV=1	sp|P61586|RHOA_HUMAN
683	Complement component 1 Q subcomponent-binding protein, mitochondrial OS=Homo sapiens GN=C1QBP PE=1 SV=1	sp|Q07021|C1QBP_HUMAN
684	Guanine nucleotide-binding protein G(I)/G(S)/G(O) subunit gamma-12 OS=Homo sapiens GN=GNG12 PE=1 SV=3	sp|Q9UBI6|GBG12_HUMAN
685	Peroxiredoxin-4 (Fragment) OS=Homo sapiens GN=PRDX4 PE=4 SV=1	tr|H7C3T4|H7C3T4_HUMAN (+1)
686	Isoform Del-701 of Signal transducer and activator of transcription 3 OS=Homo sapiens GN=STAT3	sp|P40763-2|STAT3_HUMAN (+3)
687	Tubulin-specific chaperone A OS=Homo sapiens GN=TBCA PE=1 SV=3	sp|O75347|TBCA_HUMAN (+3)
688	Unconventional myosin-Ig OS=Homo sapiens GN=MYO1G PE=1 SV=2	sp|B0I1T2|MYO1G_HUMAN
689	Eukaryotic translation initiation factor 3 subunit F OS=Homo sapiens GN=EIF3F PE=1 SV=1	sp|O00303|EIF3F_HUMAN (+2)
690	Isoform 2 of Proteasome subunit alpha type-7 OS=Homo sapiens GN=PSMA7	sp|O14818-2|PSA7_HUMAN (+2)
691	Isoform 2 of Protein transport protein Sec24D OS=Homo sapiens GN=SEC24D	sp|O94855-2|SC24D_HUMAN (+3)
692	GDH/6PGL endoplasmic bifunctional protein OS=Homo sapiens GN=H6PD PE=1 SV=2	sp|O95479|G6PE_HUMAN (+1)
693	Adenylate kinase isoenzyme 1 OS=Homo sapiens GN=AK1 PE=1 SV=3	sp|P00568|KAD1_HUMAN (+1)
694	Retinol-binding protein 4 OS=Homo sapiens GN=RBP4 PE=1 SV=3	sp|P02753|RET4_HUMAN (+1)
695	Metallothionein-1A OS=Homo sapiens GN=MT1A PE=1 SV=2	sp|P04731|MT1A_HUMAN
696	Complement factor I OS=Homo sapiens GN=CFI PE=1 SV=2	sp|P05156|CFAI_HUMAN (+2)
697	Apolipoprotein A-IV OS=Homo sapiens GN=APOA4 PE=1 SV=3	sp|P06727|APOA4_HUMAN
698	Isoform 2 of 6-phosphofructokinase, muscle type OS=Homo sapiens GN=PFKM	sp|P08237-2|K6PF_HUMAN (+2)
699	Annexin A4 OS=Homo sapiens GN=ANXA4 PE=1 SV=4	sp|P09525|ANXA4_HUMAN (+2)
700	Insulin-degrading enzyme OS=Homo sapiens GN=IDE PE=1 SV=4	sp|P14735|IDE_HUMAN
701	Ezrin OS=Homo sapiens GN=EZR PE=1 SV=4	sp|P15311|EZRI_HUMAN (+1)
702	E3 ubiquitin-protein ligase TRIM21 OS=Homo sapiens GN=TRIM21 PE=1 SV=1	sp|P19474|RO52_HUMAN (+1)
703	Tryptase beta-2 OS=Homo sapiens GN=TPSB2 PE=1 SV=2	sp|P20231|TRYB2_HUMAN (+3)
704	rRNA 2'-O-methyltransferase fibrillarin OS=Homo sapiens GN=FBL PE=1 SV=2	sp|P22087|FBRL_HUMAN (+3)
705	Isoform 2 of 60S ribosomal protein L13 OS=Homo sapiens GN=RPL13	sp|P26373-2|RL13_HUMAN (+1)
706	Cellular retinoic acid-binding protein 2 OS=Homo sapiens GN=CRABP2 PE=1 SV=2	sp|P29373|RABP2_HUMAN (+1)
707	Guanine nucleotide-binding protein-like 1 OS=Homo sapiens GN=GNL1 PE=1 SV=2	sp|P36915|GNL1_HUMAN

708	Isoform 2 of Lysosomal Pro-X carboxypeptidase OS=Homo sapiens GN=PRCP	sp|P42785-2|PCP_HUMAN (+2)
709	Guanine nucleotide-binding protein G(q) subunit alpha OS=Homo sapiens GN=GNAQ PE=1 SV=4	sp|P50148|GNAQ_HUMAN
710	Isoform 2 of Rap1 GTPase-GDP dissociation stimulator 1 OS=Homo sapiens GN=RAP1GDS1	sp|P52306-2|GDS1_HUMAN (+5)
711	Developmentally-regulated GTP-binding protein 2 OS=Homo sapiens GN=DRG2 PE=1 SV=1	sp|P55039|DRG2_HUMAN (+1)
712	Methionine--tRNA ligase, cytoplasmic OS=Homo sapiens GN=MARS PE=1 SV=2	sp|P56192|SYMC_HUMAN (+1)
713	Eukaryotic translation initiation factor 6 OS=Homo sapiens GN=EIF6 PE=1 SV=1	sp|P56537|IF6_HUMAN
714	Isoform 2 of Actin-related protein 2/3 complex subunit 4 OS=Homo sapiens GN=ARPC4	sp|P59998-2|ARPC4_HUMAN (+4)
715	Eukaryotic translation initiation factor 3 subunit E OS=Homo sapiens GN=EIF3E PE=1 SV=1	sp|P60228|EIF3E_HUMAN (+1)
716	Protein mago nashi homolog OS=Homo sapiens GN=MAGOH PE=1 SV=1	sp|P61326|MGN_HUMAN (+2)
717	Isoform 2 of Serine/threonine-protein phosphatase PP1-alpha catalytic subunit OS=Homo sapiens GN=PPP1CA	sp|P62136-2|PP1A_HUMAN (+4)
718	40S ribosomal protein S16 OS=Homo sapiens GN=RPS16 PE=1 SV=2	sp|P62249|RS16_HUMAN (+1)
719	40S ribosomal protein S23 OS=Homo sapiens GN=RPS23 PE=1 SV=3	sp|P62266|RS23_HUMAN (+1)
720	Eukaryotic peptide chain release factor subunit 1 OS=Homo sapiens GN=ETF1 PE=1 SV=3	sp|P62495|ERF1_HUMAN (+2)
721	Isoform 2 of Ras-related protein Rab-1A OS=Homo sapiens GN=RAB1A	sp|P62820-2|RAB1A_HUMAN (+3)
722	SUMO-conjugating enzyme UBC9 OS=Homo sapiens GN=UBE2I PE=1 SV=1	sp|P63279|UBC9_HUMAN (+3)
723	1,4-alpha-glucan-branching enzyme OS=Homo sapiens GN=GBE1 PE=1 SV=3	sp|Q04446|GLGB_HUMAN (+1)
724	Single-stranded DNA-binding protein, mitochondrial OS=Homo sapiens GN=SSBP1 PE=1 SV=1	sp|Q04837|SSBP_HUMAN
725	Reticulocalbin-2 OS=Homo sapiens GN=RCN2 PE=1 SV=1	sp|Q14257|RCN2_HUMAN (+2)
726	Isoform 2 of 3-hydroxyisobutyryl-CoA hydrolase, mitochondrial OS=Homo sapiens GN=HIBCH	sp|Q6NVY1-2|HIBCH_HUMAN (+1)
727	Eukaryotic translation initiation factor 3 subunit M OS=Homo sapiens GN=EIF3M PE=1 SV=1	sp|Q7L2H7|EIF3M_HUMAN
728	Isoform 2 of Thioredoxin domain-containing protein 5 OS=Homo sapiens GN=TXNDC5	sp|Q8NBS9-2|TXND5_HUMAN (+2)
729	m7GpppX diphosphatase OS=Homo sapiens GN=DCPS PE=1 SV=2	sp|Q96C86|DCPS_HUMAN
730	Nucleosome assembly protein 1-like 4 OS=Homo sapiens GN=NAP1L4 PE=1 SV=1	sp|Q99733|NP1L4_HUMAN (+2)
731	Isoform 2 of Chitinase domain-containing protein 1 OS=Homo sapiens GN=CHID1	sp|Q9BWS9-2|CHID1_HUMAN (+3)
732	Isoform 2 of Haloacid dehalogenase-like hydrolase domain-containing protein 2 OS=Homo sapiens GN=HDHD2	sp|Q9H0R4-2|HDHD2_HUMAN (+2)
733	Aminopeptidase B OS=Homo sapiens GN=RNPEP PE=1 SV=2	sp|Q9H4A4|AMPB_HUMAN (+1)
734	EH domain-containing protein 1 OS=Homo sapiens GN=EHD1 PE=1 SV=2	sp|Q9H4M9|EHD1_HUMAN
735	Isoform 2 of Adipocyte plasma membrane-associated protein OS=Homo sapiens GN=APMAP	sp|Q9HDC9-2|APMAP_HUMAN (+1)
736	Regulation of nuclear pre-mRNA domain-containing protein 1B OS=Homo sapiens GN=RPRD1B PE=1 SV=1	sp|Q9NQG5|RPR1B_HUMAN (+1)
737	Isoform 2 of Endoplasmic reticulum aminopeptidase 1 OS=Homo sapiens GN=ERAP1	sp|Q9NZ08-2|ERAP1_HUMAN (+1)

738	Protein RCC2 OS=Homo sapiens GN=RCC2 PE=1 SV=2	sp|Q9P258|RCC2_HUMAN
739	Isoform 2 of Ankyrin repeat and FYVE domain-containing protein 1 OS=Homo sapiens GN=ANKFY1	sp|Q9P2R3-2|ANFY1_HUMAN (+2)
740	Cathepsin Z OS=Homo sapiens GN=CTSZ PE=1 SV=1	sp|Q9UBR2|CATZ_HUMAN
741	Nuclear receptor-binding protein OS=Homo sapiens GN=NRBP1 PE=1 SV=1	sp|Q9UHY1|NRBP_HUMAN (+1)
742	Ras-related protein Rab-23 OS=Homo sapiens GN=RAB23 PE=1 SV=1	sp|Q9ULC3|RAB23_HUMAN
743	Eukaryotic translation initiation factor 2 subunit 1 OS=Homo sapiens GN=EIF2S1 PE=1 SV=3	sp|P05198|IF2A_HUMAN
744	40S ribosomal protein S18 OS=Homo sapiens GN=RPS18 PE=1 SV=3	sp|P62269|RS18_HUMAN
745	Transcriptional activator protein Pur-beta OS=Homo sapiens GN=PURB PE=1 SV=3	sp|Q96QR8|PURB_HUMAN
746	Isoform 2 of Cysteine--tRNA ligase, cytoplasmic OS=Homo sapiens GN=CARS	sp|P49589-2|SYCC_HUMAN (+4)
747	60S ribosomal protein L23 OS=Homo sapiens GN=RPL23 PE=1 SV=1	sp|P62829|RL23_HUMAN
748	D-dopachrome decarboxylase OS=Homo sapiens GN=DDT PE=1 SV=3	sp|P30046|DOPD_HUMAN (+1)
749	Phenylalanine--tRNA ligase alpha subunit OS=Homo sapiens GN=FARSA PE=1 SV=3	sp|Q9Y285|SYFA_HUMAN (+4)
750	Methionine synthase OS=Homo sapiens GN=MTR PE=1 SV=2	sp|Q99707|METH_HUMAN (+1)
751	Enhancer of rudimentary homolog OS=Homo sapiens GN=ERH PE=1 SV=1	sp|P84090|ERH_HUMAN
752	Isoform 2 of Podocan OS=Homo sapiens GN=PODN	sp|Q7Z5L7-2|PODN_HUMAN (+3)
753	Isoform 2 of Mannose-6-phosphate isomerase OS=Homo sapiens GN=MPI	sp|P34949-2|MPI_HUMAN (+3)
754	Isoform 2 of Ribose-phosphate pyrophosphokinase 2 OS=Homo sapiens GN=PRPS2	sp|P11908-2|PRPS2_HUMAN (+3)
755	Heat shock protein beta-7 OS=Homo sapiens GN=HSPB7 PE=1 SV=1	sp|Q9UBY9|HSPB7_HUMAN (+5)
756	Inorganic pyrophosphatase OS=Homo sapiens GN=PPA1 PE=2 SV=1	tr|Q5SQT6|Q5SQT6_HUMAN (+1)
757	WD40 repeat-containing protein SMU1 OS=Homo sapiens GN=SMU1 PE=1 SV=2	sp|Q2TAY7|SMU1_HUMAN
758	Isoform 3 of Agrin OS=Homo sapiens GN=AGRN	sp|O00468-3|AGRIN_HUMAN (+6)
759	Eukaryotic translation initiation factor 3 subunit K OS=Homo sapiens GN=EIF3K PE=1 SV=1	sp|Q9UBQ5|EIF3K_HUMAN (+4)
760	Calcium-binding protein 39 OS=Homo sapiens GN=CAB39 PE=1 SV=1	sp|Q9Y376|CAB39_HUMAN
761	Isoform Non-brain of Clathrin light chain A OS=Homo sapiens GN=CLTA	sp|P09496-2|CLCA_HUMAN (+1)
762	S-adenosylmethionine synthase isoform type-2 OS=Homo sapiens GN=MAT2A PE=1 SV=1	sp|P31153|METK2_HUMAN
763	Isoform CNPI of 2',3'-cyclic-nucleotide 3'-phosphodiesterase OS=Homo sapiens GN=CNP	sp|P09543-2|CN37_HUMAN (+2)
764	Thioredoxin domain-containing protein 17 OS=Homo sapiens GN=TXNDC17 PE=1 SV=1	sp|Q9BRA2|TXD17_HUMAN
765	Nascent polypeptide-associated complex subunit alpha OS=Homo sapiens GN=NACA PE=2 SV=1	tr|E9PAV3|E9PAV3_HUMAN
