# Supplementary material for: A novel human leiomyoma tissue derived matrix for cell culture studies
Source: BMC Cancer. 2015 Dec 16;15:981. doi: 10.1186/s12885-015-1944-z (PMC4682271; doi:10.1186/s12885-015-1944-z)
Supplement: Additional file 4: Table S2. — The pH-measurements of Myogel and Matrigel®. (PDF 111 kb) [file 12885_2015_1944_MOESM4_ESM.pdf]

Table S2. The pH-measurements of Myogel and Matrigel®

	Myogel with HSC-3	Matrigel® with HSC-3
0 h	7.0 - 7.5	8.0 - 8.5
17 h	7.0 - 7.5	8.0
48 h	6.5 - 7.0	6.0 - 6.5
