# Supplementary material for: A novel human leiomyoma tissue derived matrix for cell culture studies
Source: BMC Cancer. 2015 Dec 16;15:981. doi: 10.1186/s12885-015-1944-z (PMC4682271; doi:10.1186/s12885-015-1944-z)
Supplement: Additional file 5: Table S3. — Microarray results showing genes with FC 1.5 or more when the HSC-3 cells cultured on the top of plastic were compared to the corresponding cells grown on Myogel. (PDF 1329 kb) [file 12885_2015_1944_MOESM5_ESM.pdf]

Table S3. Microarray results showing genes with FC 1.5 or more when the HSC-3 cells cultured on the top of plastic were compared to the corresponding cells grown on Myogel

probe set	gene	Accession	EntrezGene	Description	**Plastic (HG-U1**	Plastic (HG-U1	**Myogel (HG-U1**	Myogel (HG-U1	**fold change**	lower bound o	upper bound o	difference of mear	filtered
240496_at	Centaurin, gamma 3	AI652000	116988	gb:AI652000 /DB_XREF=gi:4735	29,35	25,26	2828,81	0,26	96,37	39,9	100000000	2799,45	*
209189_at	v-fos FBJ murine osteosarcoma viral oncoge	BC004490	2353	gb:BC004490.1 /DB_XREF=gi:13	144,91	17,19	676,2	3,68	4,67	3,9	5,8	531,28	*
1565867_a_at	KIAA0663 gene product	W01876	9877	gb:W01876 /DB_XREF=gi:12740	122,77	7,94	438,99	2,22	3,58	3,23	4	316,21	*
201693_s_at	early growth response 1	AV733950	1958	gb:AV733950 /DB_XREF=gi:1085	537,23	24,63	1859,92	7,11	3,46	3,22	3,75	1322,69	*
1554860_at	protein tyrosine phosphatase, non-receptor	AF394064	5778	gb:AF394064.1 /DB_XREF=gi:21	73,52	4,5	249,35	1,33	3,39	3,08	3,77	175,83	*
201461_s_at	mitogen-activated protein kinase-activated	NM_004759	9261	gb:NM_004759.1 /DB_XREF=gi:	73,39	9,53	240,04	2,91	3,27	2,69	4,16	166,65	*
227404_s_at	Early growth response 1	AI459194	1958	gb:AI459194 /DB_XREF=gi:4311	470,96	69,33	1527,07	21,38	3,24	2,61	4,28	1056,11	*
244396_at	Ras-GTPase-activating protein SH3-domain-	BE673925	10146	gb:BE673925 /DB_XREF=gi:1003	347,42	8,95	1113,1	2,79	3,2	3,07	3,35	765,67	*
201694_s_at	early growth response 1	NM_001964	1958	gb:NM_001964.1 /DB_XREF=gi:	1700,72	79,97	5317,7	25,58	3,13	2,9	3,39	3616,99	*
1559561_at	F-box protein, helicase, 18	AL832251	84893	gb:AL832251.1 /DB_XREF=gi:21	76,32	0,4	237,56	0,13	3,11	3,09	3,14	161,24	*
1559517_a_at	spire homolog 1 (Drosophila)	AL833817	56907	gb:AL833817.1 /DB_XREF=gi:21	133,35	10,97	395,88	3,69	2,97	2,61	3,44	262,53	*
238687_x_at	hypothetical protein FLJ20582	AV753065	54989	gb:AV753065 /DB_XREF=gi:1091	112,1	13,7	329,84	4,65	2,94	2,45	3,69	217,74	*
224580_at	Solute carrier family 38, member 1	BF515894	81539	gb:BF515894 /DB_XREF=gi:1160	371,01	15,91	970,18	6,08	2,62	2,44	2,82	599,18	*
1558960_a_at	Milk fat globule-EGF factor 8 protein	BG740880	4240	gb:BG740880 /DB_XREF=gi:1405	117,71	0,75	302,79	0,29	2,57	2,55	2,6	185,07	*
1561813_at	MRNA full length insert cDNA clone EUROIM	AL109711		gb:AL109711.1 /DB_XREF=gi:56	122,44	2,64	315,15	1,02	2,57	2,48	2,67	192,71	*
1566340_at	RNA, U6C small nucleolar	BG944179	319103	gb:BG944179 /DB_XREF=gi:1434	85,37	7,17	213,9	2,86	2,51	2,2	2,91	128,53	*
235476_at	tripartite motif-containing 59	AW182459	286827	gb:AW182459 /DB_XREF=gi:645	273,4	10,28	677,7	4,15	2,48	2,33	2,64	404,31	*
217502_at	interferon-induced protein with tetratricope	BE888744	3433	gb:BE888744 /DB_XREF=gi:1034	123,55	9,64	305,59	3,9	2,47	2,19	2,84	182,04	*
222880_at	v-akt murine thymoma viral oncogene hom	AF135794	10000	gb:AF135794.1 /DB_XREF=gi:45	304,81	18,31	737,63	7,57	2,42	2,2	2,69	432,82	*
238831_at	Transmembrane protein 33	BF114679	55161	gb:BF114679 /DB_XREF=gi:1098	112,09	10,52	268,11	4,4	2,39	2,07	2,83	156,02	*
227062_at	trophoblast-derived noncoding RNA	AU155361	283131	gb:AU155361 /DB_XREF=gi:1101	162,23	5,83	388,37	2,43	2,39	2,26	2,55	226,14	*
213286_at	zinc finger RNA binding protein	BF445199	51663	gb:BF445199 /DB_XREF=gi:1151	494,42	27,74	1174,28	11,68	2,38	2,17	2,62	679,86	*
241701_at	Rho GTPase activating protein 21	BF369489	57584	gb:BF369489 /DB_XREF=gi:1133	192,52	13,8	454,38	5,85	2,36	2,11	2,68	261,86	*
216555_at	gb:AK026712.1 /DB_XREF=gi:10439629 /FE	AK026712		gb:AK026712.1 /DB_XREF=gi:10	110,37	16,94	260,57	7,17	2,36	1,88	3,17	150,2	*
208151_x_at	DEAD (Asp-Glu-Ala-Asp) box polypeptide 17	NM_030881	10521	gb:NM_030881.1 /DB_XREF=gi:	482,28	16,6	1132,38	7,07	2,35	2,22	2,49	650,11	*
242160_at	Syntaxin 17	AI733506	55014	gb:AI733506 /DB_XREF=gi:5054	100,41	8,33	234,97	3,56	2,34	2,05	2,72	134,56	*
1560017_at	SMILE protein	AK074973	160418	gb:AK074973.1 /DB_XREF=gi:22	245	15,42	565,8	6,68	2,31	2,09	2,58	320,8	*
1570347_at	MAX-like protein X	BC014537	6945	gb:BC014537.1 /DB_XREF=gi:15	146,94	7,15	334,77	3,14	2,28	2,11	2,48	187,83	*
224771_at	neuron navigator 1	AI937060	89796	gb:AI937060 /DB_XREF=gi:5675	90,87	4,67	206,45	2,06	2,27	2,09	2,49	115,58	*
33148_at	zinc finger RNA binding protein	AI459274	51663	Cluster Incl. AI459274:tk11f11.x	438,92	9,57	991,88	4,24	2,26	2,18	2,35	552,96	*
224321_at	transmembrane protein with EGF-like and t	AB004064	23671	gb:AB004064.1 /DB_XREF=gi:69	352,23	13,33	787,48	5,96	2,24	2,1	2,39	435,25	*
210210_at	myelin protein zero-like 1	AF181660	9019	gb:AF181660.1 /DB_XREF=gi:60	171,79	9,87	383,61	4,42	2,23	2,04	2,47	211,82	*
217448_s_at	chromosome 14 open reading frame 92 ///	AL117508	85412 /// 987	gb:AL117508.1 /DB_XREF=gi:59	265,48	23,3	590,26	10,48	2,22	1,94	2,61	324,78	*
238506_at	hypothetical protein LOC116064	BG285274	116064	gb:BG285274 /DB_XREF=gi:1303	278,2	16,82	606,1	7,72	2,18	1,98	2,42	327,9	*
1561093_at	putative UST1-like organic anion transporte	AK091990	387601	gb:AK091990.1 /DB_XREF=gi:21	175,28	25,68	382,79	11,76	2,18	1,75	2,89	207,52	*
242408_at	serine/threonine/tyrosine interacting protei	AW968935	6815	gb:AW968935 /DB_XREF=gi:815	558,22	14,33	1209,63	6,61	2,17	2,08	2,26	651,41	*
235588_at	establishment of cohesion 1 homolog 2 (S. c	AA740849	157570	gb:AA740849 /DB_XREF=gi:2779	245,94	21,7	525,28	10,16	2,14	1,86	2,51	279,34	*
227454_at	TAO kinase 1	AB037782	57551	gb:AB037782.1 /DB_XREF=gi:72	512,14	15,22	1093,06	7,13	2,13	2,03	2,25	580,91	*
211051_s_at	exostoses (multiple)-like 3 /// exostoses (m	BC006363	2137	gb:BC006363.1 /DB_XREF=gi:13	134,28	12,53	285,25	5,9	2,12	1,83	2,52	150,97	*
209013_x_at	triple functional domain (PTPRF interacting)	AF091395	7204	gb:AF091395.1 /DB_XREF=gi:36	228,91	16,97	484,65	8,01	2,12	1,88	2,42	255,74	*
1555546_a_at	protein O-fucosyltransferase 2	AY066015	23275	gb:AY066015.1 /DB_XREF=gi:18	113,82	6,05	241,7	2,85	2,12	1,95	2,33	127,88	*
201473_at	jun B proto-oncogene	NM_002229	3726	gb:NM_002229.1 /DB_XREF=gi:	201,48	30,58	424,41	14,52	2,11	1,67	2,82	222,93	*
201728_s_at	KIAA0100 gene product	AA904674	9703	gb:AA904674 /DB_XREF=gi:3039	271,43	16,89	573,39	8	2,11	1,91	2,36	301,96	*
231735_s_at	PRO1073 protein	NM_014086	29005	gb:NM_014086.1 /DB_XREF=gi:	1548,62	23,67	3252,22	11,27	2,1	2,05	2,16	1703,6	*
212107_s_at	DEAH (Asp-Glu-Ala-His) box polypeptide 9	BE561014	1660	gb:BE561014 /DB_XREF=gi:9804	436,93	21,75	913,04	10,41	2,09	1,93	2,28	476,11	*
212831_at	EGF-like-domain, multiple 5	BF110421	1955	gb:BF110421 /DB_XREF=gi:1094	94,29	3,37	196,68	1,61	2,09	1,97	2,22	102,39	*
235645_at	establishment of cohesion 1 homolog 1 (S. c	AW501507	114799	gb:AW501507 /DB_XREF=gi:711	178,09	7,84	371,54	3,76	2,09	1,94	2,25	193,45	*

200951_s_at	cyclin D2	AW026491	894	gb:AW026491 /DB_XREF=gi:588	995,24	37,86	2074,14	18,17	2,08	1,96	2,23	1078,91	*
204909_at	DEAD (Asp-Glu-Ala-Asp) box polypeptide 6	NM_004397	1656	gb:NM_004397.2 /DB_XREF=gi:1	278,29	7,24	580,03	3,48	2,08	2	2,18	301,74	*
234347_s_at	density-regulated protein	AF038554	8562	gb:AF038554.1 /DB_XREF=gi:28	243,5	14,01	507,07	6,73	2,08	1,9	2,31	263,57	*
213956_at	centrosome-associated protein 350	AW299294	9857	gb:AW299294 /DB_XREF=gi:670	180,57	13,45	375,42	6,47	2,08	1,85	2,38	194,85	*
200727_s_at	ARP2 actin-related protein 2 homolog (yeas	AA699583	10097	gb:AA699583 /DB_XREF=gi:270	1718,78	63,96	3532,32	31,12	2,06	1,93	2,19	1813,54	*
48659_at	hypothetical protein FLJ12438	W60802	60672	Cluster Incl. W60802:zd27e02.s1	495,03	16	1014,6	7,81	2,05	1,94	2,17	519,57	*
235852_at	Stonin 2	BE379761	85439	gb:BE379761 /DB_XREF=gi:9325	388,17	12,45	796,05	6,07	2,05	1,95	2,17	407,88	*
1555153_s_at	FCH domain only 2	BC014311	115548	gb:BC014311.1 /DB_XREF=gi:15	107,15	6,8	219,63	3,32	2,05	1,85	2,29	112,48	*
208178_x_at	triple functional domain (PTPRF interacting)	NM_007118	7204	gb:NM_007118.1 /DB_XREF=gi:	270,61	18,65	551,35	9,15	2,04	1,82	2,3	280,74	*
1555384_a_at	c-Mpl binding protein	BC022377	113251	gb:BC022377.1 /DB_XREF=gi:18	156,51	8,48	319,5	4,16	2,04	1,87	2,25	162,99	*
221220_s_at	SCY1-like 2 (S. cerevisiae)	NM_017988	55681	gb:NM_017988.1 /DB_XREF=gi:8	168,91	13,68	342,63	6,74	2,03	1,78	2,35	173,72	*
1553304_at	family with sequence similarity 61, member	NM_144703	149986	gb:NM_144703.1 /DB_XREF=gi:2	216,31	13,4	435,53	6,66	2,01	1,82	2,25	219,22	*
236207_at	sperm specific antigen 2	BE083088	6744	gb:BE083088 /DB_XREF=gi:8473	130,11	7,04	257,12	3,56	1,98	1,81	2,17	127,01	*
214052_x_at	BAT2 domain containing 1	AW301305	23215	gb:AW301305 /DB_XREF=gi:671	175,05	7,52	346,08	3,8	1,98	1,84	2,13	171,03	*
232198_at	CDNA FLJ12676 fis, clone NT2RM4002383	BF509125		gb:BF509125 /DB_XREF=gi:1159	192,91	12,64	382,03	6,38	1,98	1,78	2,23	189,13	*
200917_s_at	signal recognition particle receptor ('docking	BG474541	6734	gb:BG474541 /DB_XREF=gi:1340	119,42	6,5	235,51	3,29	1,97	1,8	2,17	116,09	*
223888_s_at	leucyl-tRNA synthetase	AF151026	51520	gb:AF151026.1 /DB_XREF=gi:71	501,62	14,81	988,61	7,51	1,97	1,88	2,07	486,99	*
241683_at	HECT domain containing 1	AW207734	25831	gb:AW207734 /DB_XREF=gi:650	109,47	6,08	215,03	3,09	1,96	1,79	2,17	105,56	*
213688_at	calmodulin 1 (phosphorylase kinase, delta)	N25325	801	gb:N25325 /DB_XREF=gi:113947	454,37	11,48	883,27	5,91	1,94	1,86	2,03	428,9	*
205809_s_at	Wiskott-Aldrich syndrome-like	BE504979	8976	gb:BE504979 /DB_XREF=gi:9707	778,76	26,87	1507,7	13,88	1,94	1,83	2,06	728,95	*
214007_s_at	PTK9 protein tyrosine kinase 9	AW665024	5756	gb:AW665024 /DB_XREF=gi:745	325,42	19,76	628,1	10,24	1,93	1,75	2,15	302,68	*
201208_s_at	tumor necrosis factor, alpha-induced protei	BC001643	7126	gb:BC001643.1 /DB_XREF=gi:12	122,62	15,49	236,18	8,04	1,93	1,58	2,45	113,56	*
208664_s_at	tetratricopeptide repeat domain 3	AU131711	7267	gb:AU131711 /DB_XREF=gi:109	289,27	6,4	558,85	3,31	1,93	1,86	2,01	269,58	*
202081_at	immediate early response 2	NM_004907	9592	gb:NM_004907.1 /DB_XREF=gi:4	1053,57	12,89	2033,52	6,68	1,93	1,89	1,97	979,96	*
236957_at	cell division cycle associated 2	AI248208	157313	gb:AI248208 /DB_XREF=gi:3843	592,12	30,09	1140,7	15,62	1,93	1,77	2,11	548,58	*
40284_at	forkhead box A2	AB028021	3170	Cluster Incl. AB028021:Homo sa	115,53	3,03	222,27	1,57	1,92	1,84	2,01	106,74	*
220946_s_at	huntingtin interacting protein B	NM_014159	29072	gb:NM_014159.1 /DB_XREF=gi:7	204,73	14,4	393,12	7,5	1,92	1,71	2,18	188,4	*
89948_at	Cluster Incl. AI743331:wg91f07.x1 Homo sa	AI743331		Cluster Incl. AI743331:wg91f07.x	218,05	12,61	419,07	6,56	1,92	1,75	2,13	201,02	*
214316_x_at	Calreticulin	AI378706	811	gb:AI378706 /DB_XREF=gi:4188	115,96	10,25	222	5,35	1,91	1,66	2,25	106,04	*
209344_at	tropomyosin 4	BC002827	7171	gb:BC002827.1 /DB_XREF=gi:12	662,85	16,04	1268,42	8,38	1,91	1,84	2	605,57	*
212439_at	inositol hexaphosphate kinase 1	BE614199	9807	gb:BE614199 /DB_XREF=gi:9895	165,04	18,52	312,84	9,77	1,9	1,59	2,34	147,8	*
224189_x_at	ets homologous factor	AF124438	26298	gb:AF124438.1 /DB_XREF=gi:48	336,33	15,29	637,75	8,06	1,9	1,76	2,05	301,42	*
242665_at	formin-like 2	AL042120	114793	gb:AL042120 /DB_XREF=gi:5421	265,33	13,48	503,42	7,11	1,9	1,74	2,08	238,09	*
208893_s_at	dual specificity phosphatase 6	BC005047	1848	gb:BC005047.1 /DB_XREF=gi:13	680,19	36,29	1283,35	19,23	1,89	1,73	2,07	603,16	*
227248_at	hypothetical protein FLJ21019	AL563218	79990	gb:AL563218 /DB_XREF=gi:1291	117,46	8,53	221,44	4,53	1,89	1,68	2,15	103,98	*
204538_x_at	nuclear pore complex interacting protein //,	NM_006985	7 /// 440341 //	gb:NM_006985.1 /DB_XREF=gi:5	350,97	14,37	664,58	7,59	1,89	1,77	2,04	313,61	*
214474_at	protein kinase, AMP-activated, beta 2 non-c	NM_005399	5565	gb:NM_005399.1 /DB_XREF=gi:4	315,87	9,65	590,13	5,17	1,87	1,78	1,97	274,26	*
204535_s_at	RE1-silencing transcription factor	AI978576	5978	gb:AI978576 /DB_XREF=gi:5803	116,13	2,16	217,39	1,15	1,87	1,81	1,93	101,26	*
202323_s_at	acyl-Coenzyme A binding domain containing	AI636775	64746	gb:AI636775 /DB_XREF=gi:4688	629,86	31,7	1175,34	16,99	1,87	1,72	2,04	545,48	*
1565765_x_at	Hypothetical protein FLJ14834	AL832478	84935	gb:AL832478.1 /DB_XREF=gi:21	567,98	6,52	1059,66	3,5	1,87	1,83	1,9	491,68	*
204827_s_at	cyclin F	U17105	899	gb:U17105.1 /DB_XREF=gi:5767	163,7	14,82	303,67	7,99	1,86	1,6	2,19	139,97	*
1556651_at	Wolf-Hirschhorn syndrome candidate 2	CA430403	7469	gb:CA430403 /DB_XREF=gi:2479	188,71	3,72	350,64	2	1,86	1,8	1,92	161,93	*
243801_x_at	Mitochondrial ribosomal protein L30	AA971709	51263	gb:AA971709 /DB_XREF=gi:3146	437,8	15,42	811,31	8,32	1,85	1,75	1,97	373,51	*
221753_at	slingshot homolog 1 (Drosophila)	AI651213	54434	gb:AI651213 /DB_XREF=gi:4735	228,87	9,82	423,06	5,31	1,85	1,72	1,99	194,19	*
226543_at	muted homolog (mouse)	AI768114	63915	gb:AI768114 /DB_XREF=gi:5234	421,14	31,73	777,79	17,18	1,85	1,63	2,12	356,66	*
243648_at	gb:AA280627 /DB_XREF=gi:1924737 /DB_XI	AA280627		gb:AA280627 /DB_XREF=gi:1924	640,5	23,21	1187,44	12,52	1,85	1,75	1,98	546,94	*
236940_at	Transcribed locus, weakly similar to XP_539	W60647		gb:W60647 /DB_XREF=gi:13676	192,53	6,38	355,75	3,46	1,85	1,75	1,96	163,22	*
233827_s_at	suppressor of Ty 16 homolog (S. cerevisiae)	AK024072	11198	gb:AK024072.1 /DB_XREF=gi:10	1269,59	44,95	2339,72	24,39	1,84	1,74	1,96	1070,13	*
235507_at	similar to hypothetical protein FLJ10883	AA461195	115294	gb:AA461195 /DB_XREF=gi:2186	394,92	26,84	726,91	14,58	1,84	1,65	2,08	331,98	*
213816_s_at	met proto-oncogene (hepatocyte growth fa	AA005141	4233	gb:AA005141 /DB_XREF=gi:1448	299,65	13,76	547,4	7,53	1,83	1,69	1,98	247,75	*

219679_s_at	WW domain containing adaptor with coiled	NM_018604	51322	gb:NM_018604.1 /DB_XREF=gi:8	507,65	15,02	930,93	8,19	1,83	1,74	1,93	423,28	*
1316_at	thyroid hormone receptor, alpha (erythrobl	X55005	7067	X55005 /FEATURE=mRNA /DEFI	238,7	15,78	435,26	8,65	1,82	1,64	2,05	196,56	*
233819_s_at	zinc finger protein 294	AK023499	26046	gb:AK023499.1 /DB_XREF=gi:104	404,36	16,97	734,69	9,34	1,82	1,69	1,96	330,33	*
1566887_x_at	LOC441699	AY094612	441699	gb:AY094612.1 /DB_XREF=gi:212	175,59	1,01	318,86	0,55	1,82	1,8	1,83	143,27	*
213813_x_at	gb:AI345238 /DB_XREF=gi:4082444 /DB_XR	AI345238		gb:AI345238 /DB_XREF=gi:40824	398,03	21,09	724,43	11,59	1,82	1,67	2	326,41	*
221765_at	gb:AI378044 /DB_XREF=gi:4187897 /DB_XR	AI378044		gb:AI378044 /DB_XREF=gi:41878	1274,84	39,6	2322,49	21,74	1,82	1,73	1,92	1047,65	*
202476_s_at	tubulin, gamma complex associated protein	BF002130	10844	gb:BF002130 /DB_XREF=gi:1070	137,27	8,52	247,92	4,72	1,81	1,63	2,02	110,65	*
242352_at	Nipped-B homolog (Drosophila)	AW272262	25836	gb:AW272262 /DB_XREF=gi:665	1006,58	30,5	1823,07	16,84	1,81	1,72	1,91	816,49	*
211159_s_at	protein phosphatase 2, regulatory subunit B	AB000635	5528	gb:AB000635.1 /DB_XREF=gi:218	253,94	6,66	457,44	3,7	1,8	1,72	1,89	203,5	*
220800_s_at	tropomodulin 3 (ubiquitous)	NM_014547	29766	gb:NM_014547.1 /DB_XREF=gi:7	274,27	15,44	493,75	8,58	1,8	1,64	1,99	219,48	*
218273_s_at	protein phosphatase 2C, magnesium-depen	NM_018444	54704	gb:NM_018444.1 /DB_XREF=gi:8	434,42	10,53	776,44	5,89	1,79	1,72	1,86	342,01	*
238419_at	pleckstrin homology-like domain, family B, r	T68150	90102	gb:T68150 /DB_XREF=gi:679298	319,48	18,76	572,84	10,46	1,79	1,63	1,99	253,36	*
1570126_at	Homo sapiens, clone IMAGE:5015646, mRN	BC033216		gb:BC033216.1 /DB_XREF=gi:231	206,79	4,55	370,98	2,54	1,79	1,73	1,86	164,19	*
211949_s_at	nucleolar and coiled-body phosphoprotein 1	AI355279	9221	gb:AI355279 /DB_XREF=gi:40954	578,41	20,56	1027,18	11,58	1,78	1,67	1,89	448,76	*
219930_at	Kruppel-like factor 8	NM_007250	11279	gb:NM_007250.1 /DB_XREF=gi:6	209,81	5,09	374,33	2,85	1,78	1,71	1,86	164,52	*
213971_s_at	suppressor of zeste 12 homolog (Drosophila	AI924660	23512	gb:AI924660 /DB_XREF=gi:56606	458,84	17,47	814,5	9,84	1,78	1,67	1,9	355,66	*
224227_s_at	B double prime 1, subunit of RNA polymeras	AF298152	55814	gb:AF298152.1 /DB_XREF=gi:110	219,13	8,75	389,9	4,92	1,78	1,66	1,91	170,77	*
223195_s_at	sestrin 2	BF131886	83667	gb:BF131886 /DB_XREF=gi:1097	243,22	11,08	433,84	6,21	1,78	1,65	1,93	190,63	*
1567224_at	Lipoma cell line Li-538/SV40 ectopic sequen	U29113		gb:U29113.1 /DB_XREF=gi:9400	252,03	19,6	447,95	11,03	1,78	1,56	2,05	195,92	*
218906_x_at	likely ortholog of kinesin light chain 2	NM_022822	64837	gb:NM_022822.1 /DB_XREF=gi:1	912,64	35,54	1615,49	20,08	1,77	1,66	1,9	702,86	*
226531_at	hypothetical protein FLJ14466	AL530596	84876	gb:AL530596 /DB_XREF=gi:1279	346,66	20,79	615,23	11,71	1,77	1,61	1,98	268,57	*
206383_s_at	Ras-GTPase activating protein SH3 domain-b	NM_012297	9908	gb:NM_012297.1 /DB_XREF=gi:7	367,27	13,33	647,33	7,56	1,76	1,66	1,88	280,07	*
210057_at	PI-3-kinase-related kinase SMG-1	U32581	23049	gb:U32581.2 /DB_XREF=gi:5542	239,35	5,69	420,6	3,24	1,76	1,69	1,83	181,26	*
212650_at	EH domain binding protein 1	BF116032	23301	gb:BF116032 /DB_XREF=gi:1098	493,72	22,58	870,25	12,81	1,76	1,63	1,91	376,54	*
216074_x_at	KIBRA protein	AK001727	23286	gb:AK001727.1 /DB_XREF=gi:702	202,72	15,23	353,93	8,72	1,75	1,54	2	151,21	*
1560020_at	DnaJ (Hsp40) homolog, subfamily C, membe	BC043583	23317	gb:BC043583.1 /DB_XREF=gi:276	147,07	10,32	257,47	5,89	1,75	1,56	1,99	110,4	*
223546_x_at	LUC7-like (S. cerevisiae)	AY005111	55692	gb:AY005111.1 /DB_XREF=gi:108	141,64	13,03	248,15	7,44	1,75	1,51	2,08	106,5	*
205717_x_at	protocadherin gamma subfamily C, 3 /// pro	NM_002588	56105 /// 5610	gb:NM_002588.1 /DB_XREF=gi:4	269,78	13,66	470,97	7,83	1,75	1,6	1,91	201,19	*
1559436_x_at	Arrestin, beta 2	AL832061	409	gb:AL832061.1 /DB_XREF=gi:217	553,94	9,95	963,02	5,73	1,74	1,69	1,79	409,08	*
237252_at	thrombomodulin	AW119113	7056	gb:AW119113 /DB_XREF=gi:608	293,48	7,72	509,8	4,44	1,74	1,66	1,82	216,32	*
203950_s_at	chloride channel 6	NM_001286	1185	gb:NM_001286.1 /DB_XREF=gi:4	150,82	11,53	260,24	6,68	1,73	1,52	1,99	109,43	*
208892_s_at	dual specificity phosphatase 6	BC003143	1848	gb:BC003143.1 /DB_XREF=gi:131	1024,56	23,17	1774,91	13,38	1,73	1,67	1,8	750,35	*
219850_s_at	ets homologous factor	NM_012153	26298	gb:NM_012153.1 /DB_XREF=gi:6	427,2	19,81	740,81	11,42	1,73	1,6	1,88	313,61	*
218969_at	mitochondria-associated protein involved in	NM_016069	51025	gb:NM_016069.1 /DB_XREF=gi:7	435,17	24,62	754,56	14,2	1,73	1,58	1,92	319,39	*
219560_at	hypothetical protein FLJ21125	NM_024627	79680	gb:NM_024627.1 /DB_XREF=gi:1	155,95	18,7	269,9	10,81	1,73	1,43	2,17	113,95	*
224917_at	gb:BF674052 /DB_XREF=gi:11947947 /DB_X	BF674052		gb:BF674052 /DB_XREF=gi:1194	680,28	29,92	1179,19	17,26	1,73	1,61	1,88	498,91	*
201635_s_at	fragile X mental retardation, autosomal hon	AI990766	8087	gb:AI990766 /DB_XREF=gi:58376	2319,37	70,88	3981,39	41,29	1,72	1,63	1,81	1662,02	*
1553106_at	hypothetical protein FLJ37562	NM_152409	134553	gb:NM_152409.1 /DB_XREF=gi:2	672,56	32,25	1158,05	18,73	1,72	1,59	1,88	485,49	*
212952_at	Calreticulin	AA910371	811	gb:AA910371 /DB_XREF=gi:3049	484,62	28,6	829,42	16,71	1,71	1,55	1,9	344,8	*
202062_s_at	sel-1 suppressor of lin-12-like (C. elegans)	NM_005065	6400	gb:NM_005065.1 /DB_XREF=gi:4	321,08	11,31	548,84	6,62	1,71	1,61	1,82	227,76	*
226637_at	Ubiquitin-conjugating enzyme E2H (UBC8 ho	AA916831	7328	gb:AA916831 /DB_XREF=gi:3056	144,92	10,05	247,6	5,88	1,71	1,52	1,94	102,68	*
1554916_a_at	jerky homolog (mouse)	BC043351	8629	gb:BC043351.1 /DB_XREF=gi:276	195,23	9,35	334,75	5,45	1,71	1,58	1,87	139,52	*
229115_at	dynein, cytoplasmic, heavy polypeptide 1	BF000332	1778	gb:BF000332 /DB_XREF=gi:1070	448,81	5,95	762,94	3,5	1,7	1,66	1,74	314,13	*
227849_at	Retinitis pigmentosa 9 (autosomal dominan	BF438270	6100	gb:BF438270 /DB_XREF=gi:1145	143,99	7,44	244,5	4,38	1,7	1,56	1,86	100,51	*
1557258_a_at	B-cell CLL/lymphoma 10	AA994334	8915	gb:AA994334 /DB_XREF=gi:3180	557,2	14,19	945,72	8,36	1,7	1,62	1,78	388,52	*
238628_s_at	hematopoietic stem/progenitor cells 176	AW827150	51693	gb:AW827150 /DB_XREF=gi:792	183,8	12,03	313,02	7,07	1,7	1,53	1,92	129,21	*
218550_s_at	leucine rich repeat containing 20	NM_018205	55222	gb:NM_018205.1 /DB_XREF=gi:8	164,47	12,81	278,79	7,56	1,7	1,49	1,96	114,31	*
208790_s_at	polymerase I and transcript release factor	AF312393	284119	gb:AF312393.1 /DB_XREF=gi:110	552,19	38,43	939,84	22,58	1,7	1,52	1,93	387,65	*
227129_x_at	hypothetical protein LOC284701 /// hypoth	AW006934	8312 /// 3998	gb:AW006934 /DB_XREF=gi:585	228,37	17,54	388,14	10,32	1,7	1,5	1,96	159,77	*
214870_x_at	nuclear pore complex interacting protein //,	AC002045	7 /// 440341 /,	gb:AC002045 /DB_XREF=gi:2951	358,39	14,65	610,06	8,6	1,7	1,59	1,83	251,68	*

241223_x_at	gb:AI821721 /DB_XREF=gi:5440800 /DB_XR	AI821721		gb:AI821721 /DB_XREF=gi:5440	1075,82	39,64	1832,87	23,27	1,7	1,6	1,82	757,05	*
211756_at	parathyroid hormone-like hormone /// para	BC005961	5744	gb:BC005961.1 /DB_XREF=gi:13	429,51	14,41	723,82	8,55	1,69	1,59	1,79	294,31	*
213799_s_at	protein tyrosine phosphatase, receptor type	BF740139	5786	gb:BF740139 /DB_XREF=gi:1206	361,45	17,76	610,75	10,51	1,69	1,56	1,85	249,31	*
208947_s_at	regulator of nonsense transcripts 1	U59323	5976	gb:U59323.1 /DB_XREF=gi:1633	159,21	4,7	269,3	2,78	1,69	1,61	1,78	110,09	*
202509_s_at	tumor necrosis factor, alpha-induced protei	AI862445	7127	gb:AI862445 /DB_XREF=gi:5526	145,88	12,62	247,13	7,45	1,69	1,47	1,99	101,25	*
32062_at	leucine rich repeat containing 14	D25216	9684	Cluster Incl. D25216:Human mR	1217,42	8,93	2059,22	5,28	1,69	1,67	1,71	841,8	*
212492_s_at	jumonji domain containing 2B	AW237172	23030	gb:AW237172 /DB_XREF=gi:656	205,67	16,22	346,55	9,63	1,69	1,48	1,95	140,88	*
223365_at	DEAH (Asp-Glu-Ala-His) box polypeptide 37	BC004463	57647	gb:BC004463.1 /DB_XREF=gi:13	295,2	15,61	500,32	9,21	1,69	1,55	1,86	205,13	*
1560430_at	NTPase, KAP family P-loop domain containir	AK090919	284353	gb:AK090919.1 /DB_XREF=gi:21	230,72	6,97	390,89	4,11	1,69	1,61	1,79	160,17	*
212113_at	Similar to microtubule-associated proteins 1	AI927479	387869	gb:AI927479 /DB_XREF=gi:5663	363,18	11,56	612,49	6,85	1,69	1,6	1,78	249,31	*
225920_at	hypothetical protein LOC148413 /// similar	AW452640	8413 /// 4407	gb:AW452640 /DB_XREF=gi:699	578,75	24,56	980,9	14,49	1,69	1,58	1,83	402,16	*
244624_at	ribosomal protein S27a /// similar to bA92K	AW013893	/// 389425 //	gb:AW013893 /DB_XREF=gi:586	241,57	2,06	408,06	1,22	1,69	1,66	1,71	166,49	*
207598_x_at	X-ray repair complementing defective repai	NM_005431	7516	gb:NM_005431.1 /DB_XREF=gi:	194,1	12,06	325,44	7,19	1,68	1,51	1,88	131,34	*
224828_at	cytoplasmic polyadenylation element bindir	AV704132	80315	gb:AV704132 /DB_XREF=gi:1072	321,28	12,2	540,2	7,26	1,68	1,58	1,8	218,92	*
213485_s_at	ATP-binding cassette, sub-family C (CFTR/M	AK000002	89845	gb:AK000002.1 /DB_XREF=gi:72	303,26	25,13	508,28	14,99	1,68	1,46	1,95	205,01	*
225009_at	chemokine-like factor super family 4	AA191708	146223	gb:AA191708 /DB_XREF=gi:1780	415,75	16,53	697,72	9,85	1,68	1,57	1,8	281,97	*
1561221_x_at	Hypothetical LOC387905	BC036362	387905	gb:BC036362.1 /DB_XREF=gi:23	168,92	4,25	283,48	2,53	1,68	1,61	1,75	114,56	*
1567985_at	Similar to calreticulin	AJ012500	441884	gb:AJ012500.1 /DB_XREF=gi:544	192,26	11,08	322,4	6,6	1,68	1,52	1,86	130,14	*
225899_x_at	hypothetical protein LOC284701 /// hypoth	AL040396	9844 /// 4404	gb:AL040396 /DB_XREF=gi:5409	287,08	12,32	481,38	7,35	1,68	1,56	1,81	194,3	*
213003_s_at	gb:BF061054 /DB_XREF=gi:10819964 /DB_	BF061054		gb:BF061054 /DB_XREF=gi:1081	377,64	4,25	636,16	2,52	1,68	1,65	1,72	258,52	*
221963_x_at	Transcribed locus, strongly similar to XP_42	BE999967		gb:BE999967 /DB_XREF=gi:1070	468,11	26,68	785,12	15,9	1,68	1,52	1,86	317,02	*
204015_s_at	dual specificity phosphatase 4	BC002671	1846	gb:BC002671.1 /DB_XREF=gi:12	347,34	6,2	580,06	3,71	1,67	1,62	1,72	232,72	*
208891_at	dual specificity phosphatase 6	BC003143	1848	gb:BC003143.1 /DB_XREF=gi:13	851,57	11,66	1422,32	6,98	1,67	1,63	1,71	570,75	*
209996_x_at	pericentriolar material 1	AA931266	5108	gb:AA931266 /DB_XREF=gi:3085	180,18	7,84	301,15	4,69	1,67	1,55	1,81	120,97	*
202936_s_at	SRY (sex determining region Y)-box 9 (camp	NM_000346	6662	gb:NM_000346.1 /DB_XREF=gi:	220,74	5,24	369,56	3,13	1,67	1,61	1,75	148,82	*
1555259_at	Sterile alpha motif and leucine zipper contai	AF465843	51776	gb:AF465843.1 /DB_XREF=gi:28	188,22	7,2	315,13	4,3	1,67	1,57	1,79	126,92	*
239742_at	Tubby like protein 4	H15278	56995	gb:H15278 /DB_XREF=gi:88009	323,64	18,1	541,55	10,82	1,67	1,52	1,85	217,91	*
235009_at	family with sequence similarity 44, member	AI049791	259282	gb:AI049791 /DB_XREF=gi:3298	354,92	11,65	592,58	6,98	1,67	1,58	1,77	237,66	*
208297_s_at	ecotropic viral integration site 5 /// similar t	NM_005665	40593 /// 781	gb:NM_005665.1 /DB_XREF=gi:	401,16	13,11	667,98	7,87	1,67	1,57	1,77	266,82	*
216858_x_at	gb:AL080112.1 /DB_XREF=gi:5262539 /FEA=	AL080112		gb:AL080112.1 /DB_XREF=gi:52	318,44	10,23	531,37	6,13	1,67	1,58	1,77	212,94	*
202600_s_at	nuclear receptor interacting protein 1	AI824012	8204	gb:AI824012 /DB_XREF=gi:5444	397,15	19,82	660,5	11,92	1,66	1,53	1,82	263,35	*
214869_x_at	GTPase activating protein and VPS9 domain	AK021533	26130	gb:AK021533.1 /DB_XREF=gi:10	1036,93	14,72	1724,83	8,85	1,66	1,62	1,71	687,9	*
208721_s_at	anaphase promoting complex subunit 5	BF967271	51433	gb:BF967271 /DB_XREF=gi:1233	443,31	14,22	736,34	8,56	1,66	1,57	1,76	293,03	*
242895_x_at	Ring finger protein 39	R63837	80352	gb:R63837 /DB_XREF=gi:835716	313,21	4,23	520,9	2,55	1,66	1,62	1,7	207,7	*
1564362_x_at	hypothetical protein MGC46336	AK096248	283933	gb:AK096248.1 /DB_XREF=gi:21	275,8	12,28	457,87	7,4	1,66	1,54	1,8	182,08	*
207679_at	paired box gene 3 (Waardenburg syndrome	NM_000438	5077	gb:NM_000438.2 /DB_XREF=gi:	191,36	5,27	315,96	3,19	1,65	1,57	1,73	124,6	*
204275_at	small optic lobes homolog (Drosophila)	AI796687	6650	gb:AI796687 /DB_XREF=gi:5362	301,65	8,61	496,39	5,23	1,65	1,57	1,73	194,73	*
215568_x_at	Lysophospholipase II	AL031295	11313	gb:AL031295 /DB_XREF=gi:4376	1186,35	8,03	1952,71	4,88	1,65	1,63	1,67	766,35	*
216222_s_at	myosin X	AI561354	4651	gb:AI561354 /DB_XREF=gi:4511	1119,93	23,31	1841,96	14,18	1,64	1,59	1,71	722,03	*
209136_s_at	ubiquitin specific protease 10	BG390445	9100	gb:BG390445 /DB_XREF=gi:1328	544	18,94	891,1	11,56	1,64	1,54	1,74	347,1	*
41387_r_at	jumonji domain containing 3	AB002344	23135	Cluster Incl. AB002344:Human r	266,5	9,22	437,06	5,62	1,64	1,55	1,75	170,56	*
1555947_at	chromosome 9 open reading frame 10	AU116818	23196	gb:AU116818 /DB_XREF=gi:1093	202,67	9,25	333,23	5,63	1,64	1,52	1,79	130,55	*
222456_s_at	epithelial protein lost in neoplasm beta	BF197289	51474	gb:BF197289 /DB_XREF=gi:1108	475,32	15,41	778,91	9,4	1,64	1,55	1,74	303,59	*
224775_at	hypothetical protein FLJ10006	AW451291	55677	gb:AW451291 /DB_XREF=gi:699	265,47	20,22	434,6	12,35	1,64	1,44	1,89	169,13	*
202124_s_at	amyotrophic lateral sclerosis 2 (juvenile) chı	AV705253	66008	gb:AV705253 /DB_XREF=gi:1072	395,41	13,44	648,31	8,19	1,64	1,55	1,74	252,9	*
233702_x_at	CDNA: FLJ20946 fis, clone ADSE01819	AK024599		gb:AK024599.1 /DB_XREF=gi:10	796,23	25,19	1306,18	15,36	1,64	1,55	1,74	509,95	*
215615_x_at	Arginine-glutamic acid dipeptide (RE) repeat	AU148274	473	gb:AU148274 /DB_XREF=gi:1100	1770,15	17,12	2887,77	10,5	1,63	1,6	1,66	1117,62	*
204680_s_at	Rap guanine nucleotide exchange factor (GE	AI263837	9771	gb:AI263837 /DB_XREF=gi:3872	187,93	8,41	306,73	5,15	1,63	1,51	1,77	118,8	*
241642_x_at	tousled-like kinase 1	AI791189	9874	gb:AI791189 /DB_XREF=gi:5338	198,19	4,35	322,23	2,67	1,63	1,57	1,69	124,03	*
1555058_a_at	lysophosphatidylglycerol acyltransferase 1	BC034621	9926	gb:BC034621.1 /DB_XREF=gi:22	869,55	47,59	1420,84	29,13	1,63	1,49	1,81	551,29	*

233487_s_at	leucine rich repeat containing 8	AK024649	56262	gb:AK024649.1 /DB_XREF=gi:10	490,6	19,18	802	11,73	1,63	1,53	1,75	311,4	*
237475_x_at	CH5400	AI151104		gb:AI151104 /DB_XREF=gi:3679	763,99	15,78	1241,97	9,7	1,63	1,57	1,69	477,97	*
209457_at	dual specificity phosphatase 5	U16996	1847	gb:U16996.1 /DB_XREF=gi:6420	284,06	15,92	459,15	9,85	1,62	1,47	1,79	175,09	*
213944_x_at	guanine nucleotide binding protein (G prote	BG236220	2767	gb:BG236220 /DB_XREF=gi:1275	212,05	14,33	344,33	8,82	1,62	1,45	1,84	132,28	*
205787_x_at	KIAA0663 gene product	AI803216	9877	gb:AI803216 /DB_XREF=gi:5368	808,73	14,32	1311,44	8,83	1,62	1,57	1,67	502,71	*
201793_x_at	chromosome 1 open reading frame 16	BF509099	9887	gb:BF509099 /DB_XREF=gi:1159	422,43	12,67	684,55	7,82	1,62	1,54	1,71	262,12	*
212886_at	DKFZP434C171 protein	AL080169	26112	gb:AL080169.1 /DB_XREF=gi:52	308,27	19,33	498,58	11,95	1,62	1,45	1,82	190,32	*
201299_s_at	MOB1, Mps One Binder kinase activator-like	NM_018221	55233	gb:NM_018221.1 /DB_XREF=gi:8	2013,26	74,8	3261,59	46,17	1,62	1,52	1,73	1248,33	*
209346_s_at	phosphatidylinositol 4-kinase type II	BC003167	55361	gb:BC003167.1 /DB_XREF=gi:13	211,82	18,2	344,08	11,21	1,62	1,41	1,91	132,26	*
1558080_s_at	hypothetical protein LOC144871	BG913589	144871	gb:BG913589 /DB_XREF=gi:1429	496,28	15,22	805,97	9,37	1,62	1,54	1,72	309,68	*
201041_s_at	dual specificity phosphatase 1	NM_004417	1843	gb:NM_004417.2 /DB_XREF=gi:7	1517,13	43,77	2446,62	27,14	1,61	1,53	1,7	929,5	*
209701_at	type 1 tumor necrosis factor receptor shedc	D16217	51752	gb:D16217.1 /DB_XREF=gi:3035	339,66	11,4	547,72	7,07	1,61	1,52	1,71	208,06	*
49679_s_at	hypothetical LOC400843	AA243774	400843	Cluster Incl. AA243774:zr67d03.	375,97	15,77	606,8	9,77	1,61	1,5	1,74	230,83	*
232596_at	gb:AL354829 /DB_XREF=gi:8670914 /FEA=D	AL354829		gb:AL354829 /DB_XREF=gi:8670	809,99	31,07	1304,72	19,29	1,61	1,51	1,73	494,73	*
201791_s_at	7-dehydrocholesterol reductase	NM_001360	1717	gb:NM_001360.1 /DB_XREF=gi:4	1514,57	26,2	2429,52	16,33	1,6	1,56	1,65	914,95	*
200841_s_at	glutamyl-prolyl-tRNA synthetase	AI142677	2058	gb:AI142677 /DB_XREF=gi:3659	776,89	30,77	1239,32	19,29	1,6	1,49	1,71	462,42	*
204863_s_at	interleukin 6 signal transducer (gp130, onco	BE856546	3572	gb:BE856546 /DB_XREF=gi:1036	471,76	20,68	755,1	12,92	1,6	1,48	1,73	283,34	*
211000_s_at	interleukin 6 signal transducer (gp130, onco	AB015706	3572	gb:AB015706.1 /DB_XREF=gi:49	278,73	15	447,16	9,35	1,6	1,46	1,77	168,44	*
216450_x_at	tumor rejection antigen (gp96) 1	AK025862	7184	gb:AK025862.1 /DB_XREF=gi:10	1011,99	18,88	1614,33	11,83	1,6	1,54	1,65	602,34	*
218565_at	chromosome 9 open reading frame 114	BG223334	51490	gb:BG223334 /DB_XREF=gi:1270	223,7	15,27	357,72	9,55	1,6	1,42	1,81	134,02	*
37170_at	BMP2 inducible kinase	AB015331	55589	Cluster Incl. AB015331:Homo sa	192,03	5,45	306,7	3,41	1,6	1,52	1,68	114,67	*
216490_x_at	similar to dJ408B20.3 (novel protein similar	AL133267	442175	gb:AL133267 /DB_XREF=gi:1018	499,61	5,94	797,34	3,72	1,6	1,56	1,63	297,73	*
214974_x_at	chemokine (C-X-C motif) ligand 5	AK026546	6374	gb:AK026546.1 /DB_XREF=gi:10	416,83	1,12	663,82	0,7	1,59	1,59	1,6	246,99	*
1557227_s_at	translocated promoter region (to activated	AW235355	7175	gb:AW235355 /DB_XREF=gi:656	704,59	32,74	1118,33	20,63	1,59	1,47	1,73	413,74	*
204047_s_at	phosphatase and actin regulator 2	AW295193	9749	gb:AW295193 /DB_XREF=gi:670	180,3	18,38	286,46	11,57	1,59	1,34	1,93	106,15	*
223295_s_at	LUC7-like (S. cerevisiae)	BE049621	55692	gb:BE049621 /DB_XREF=gi:8366	303,63	13,78	481,58	8,69	1,59	1,47	1,72	177,95	*
242608_x_at	Zinc finger protein 410	AW517479	57862	gb:AW517479 /DB_XREF=gi:715	285,16	9,45	452,36	5,96	1,59	1,5	1,68	167,19	*
228633_s_at	LYST-interacting protein LIP8	AI668799	116840	gb:AI668799 /DB_XREF=gi:4833	268,6	12,74	426,6	8,02	1,59	1,46	1,73	158	*
241418_at	Hypothetical LOC344887	AI819386	344887	gb:AI819386 /DB_XREF=gi:5438	183,73	11,22	292,46	7,05	1,59	1,43	1,78	108,74	*
217878_s_at	cell division cycle 27	AI203880	996	gb:AI203880 /DB_XREF=gi:3756	2264,65	71,18	3573,72	45,11	1,58	1,49	1,67	1309,07	*
215541_s_at	diaphanous homolog 1 (Drosophila)	AK023345	1729	gb:AK023345.1 /DB_XREF=gi:10	880,97	21,74	1393,07	13,75	1,58	1,51	1,65	512,1	*
202176_at	excision repair cross-complementing rodent	NM_000122	2071	gb:NM_000122.1 /DB_XREF=gi:4	385,51	25,31	610,93	15,97	1,58	1,42	1,79	225,42	*
202118_s_at	copine III	AA541758	8895	gb:AA541758 /DB_XREF=gi:2288	1145,62	36,82	1812,82	23,27	1,58	1,5	1,68	667,2	*
203463_s_at	epsin 2	H05668	22905	gb:H05668 /DB_XREF=gi:869220	487,8	9,23	771,79	5,83	1,58	1,53	1,64	283,98	*
217678_at	solute carrier family 7, (cationic amino acid	AA488687	23657	gb:AA488687 /DB_XREF=gi:2216	681,23	25,66	1073,15	16,29	1,58	1,48	1,69	391,92	*
221985_at	DRE1 protein	AW006750	54800	gb:AW006750 /DB_XREF=gi:585	419,42	13,71	661,62	8,69	1,58	1,49	1,67	242,2	*
222027_at	Nuclear ubiquitous casein kinase and cyclin-	AW515443	64710	gb:AW515443 /DB_XREF=gi:715	879,23	36,05	1391,62	22,78	1,58	1,47	1,71	512,39	*
228986_at	oxysterol binding protein-like 8	AW978375	114882	gb:AW978375 /DB_XREF=gi:816	1103,15	32,78	1745,08	20,72	1,58	1,5	1,67	641,94	*
239486_at	Transcribed locus	BG111636		gb:BG111636 /DB_XREF=gi:1260	657,21	17,21	1037,11	10,9	1,58	1,51	1,65	379,89	*
208624_s_at	eukaryotic translation initiation factor 4 gan	BE966878	1981	gb:BE966878 /DB_XREF=gi:1177	1788,42	34,7	2816,04	22,04	1,57	1,52	1,63	1027,61	*
212240_s_at	phosphoinositide-3-kinase, regulatory subu	AI679268	5295	gb:AI679268 /DB_XREF=gi:4889	647,28	13,9	1016,78	8,85	1,57	1,51	1,63	369,49	*
203759_at	ST3 beta-galactoside alpha-2,3-sialyltransfe	NM_006278	6484	gb:NM_006278.1 /DB_XREF=gi:5	253,16	21,98	398,24	13,98	1,57	1,36	1,85	145,09	*
213494_s_at	YY1 transcription factor	AA748649	7528	gb:AA748649 /DB_XREF=gi:2788	349,6	10,28	549,45	6,54	1,57	1,49	1,66	199,84	*
215553_x_at	WD repeat domain 45	AK024315	11152	gb:AK024315.1 /DB_XREF=gi:10	543,13	5,85	853,61	3,72	1,57	1,54	1,6	310,48	*
241955_at	HECT domain containing 1	BE243270	25831	gb:BE243270 /DB_XREF=gi:9095	1378,06	48,74	2165,46	31,02	1,57	1,48	1,68	787,4	*
204324_s_at	golgi phosphoprotein 4	NM_014498	27333	gb:NM_014498.1 /DB_XREF=gi:7	688,29	7,3	1077,18	4,66	1,57	1,54	1,59	388,89	*
224469_s_at	chromosome 14 open reading frame 151 //,	BC006173	84800	gb:BC006173.1 /DB_XREF=gi:13	438,57	9,25	690,41	5,88	1,57	1,52	1,64	251,84	*
238547_at	hypothetical protein MGC39389	AI972367	124790	gb:AI972367 /DB_XREF=gi:5769	192,74	2,56	302,89	1,63	1,57	1,54	1,61	110,15	*
243724_at	gb:AW979182 /DB_XREF=gi:8170469 /DB_X	AW979182		gb:AW979182 /DB_XREF=gi:817	182,08	1,7	285,72	1,08	1,57	1,54	1,6	103,63	*
242979_at	Transcribed locus	AI474666		gb:AI474666 /DB_XREF=gi:4327	183,08	12,1	287,44	7,7	1,57	1,4	1,78	104,36	*

227547_at	Transcribed locus, weakly similar to XP_496	AA824321		gb:AA824321 /DB_XREF=gi:2896	210,38	5,03	329,77	3,21	1,57	1,5	1,64	119,39	*
209164_s_at	cytochrome b-561	BC002976	1534	gb:BC002976.1 /DB_XREF=gi:128	252,24	12,25	394,08	7,84	1,56	1,44	1,71	141,83	*
211074_at	folate receptor 1 (adult) /// folate receptor	AF000381	2348	gb:AF000381.1 /DB_XREF=gi:256	413,11	9,49	643,71	6,09	1,56	1,5	1,62	230,6	*
211094_s_at	neurofibromin 1 (neurofibromatosis, von Re	D12625	4763	gb:D12625.1 /DB_XREF=gi:2199	350,79	11,5	548,53	7,35	1,56	1,48	1,66	197,74	*
221830_at	RAP2A, member of RAS oncogene family	AI302106	5911	gb:AI302106 /DB_XREF=gi:3961	525,11	25,59	820,07	16,39	1,56	1,44	1,71	294,96	*
201070_x_at	splicing factor 3b, subunit 1, 155kDa	AI739389	23451	gb:AI739389 /DB_XREF=gi:5101	820,19	48,48	1277,13	31,13	1,56	1,41	1,74	456,94	*
223584_s_at	kelch repeat and BTB (POZ) domain containi	BF000166	25948	gb:BF000166 /DB_XREF=gi:1070	432,52	9,06	673,5	5,82	1,56	1,5	1,62	240,99	*
221649_s_at	peter pan homolog (Drosophila)	BC000535	56342	gb:BC000535.1 /DB_XREF=gi:126	554,65	11,41	867,77	7,29	1,56	1,51	1,62	313,13	*
1558822_at	AT rich interactive domain 1B (SWI1-like)	AF147412	57492	gb:AF147412.1 /DB_XREF=gi:476	309,91	1,98	481,93	1,28	1,56	1,54	1,57	172,01	*
211993_at	WNK lysine deficient protein kinase 1 /// W	AI768512	65125	gb:AI768512 /DB_XREF=gi:5235	988,07	52,37	1541,73	33,56	1,56	1,42	1,72	553,67	*
225954_s_at	midnolin	AL512725	90007	gb:AL512725.1 /DB_XREF=gi:122	656,36	9,87	1022,35	6,33	1,56	1,52	1,6	366	*
227633_at	gb:AA056145 /DB_XREF=gi:1548483 /DB_XI	AA056145		gb:AA056145 /DB_XREF=gi:1548	259,52	6,52	405,64	4,17	1,56	1,5	1,64	146,12	*
204470_at	chemokine (C-X-C motif) ligand 1 (melanom	NM_001511	2919	gb:NM_001511.1 /DB_XREF=gi:4	393,79	8,3	608,84	5,37	1,55	1,49	1,61	215,04	*
203752_s_at	jun D proto-oncogene	NM_005354	3727	gb:NM_005354.2 /DB_XREF=gi:1	832,18	18,05	1292,39	11,62	1,55	1,49	1,62	460,21	*
215498_s_at	mitogen-activated protein kinase kinase 3 /,	AA780381	5606	gb:AA780381 /DB_XREF=gi:2839	428,34	17,22	665,81	11,08	1,55	1,45	1,67	237,46	*
201039_s_at	RAD23 homolog A (S. cerevisiae)	BF572938	5886	gb:BF572938 /DB_XREF=gi:1164	805,69	49,31	1245,48	31,9	1,55	1,39	1,73	439,79	*
201085_s_at	SON DNA binding protein	AA664291	6651	gb:AA664291 /DB_XREF=gi:2618	1418,29	87,93	2202,65	56,62	1,55	1,4	1,74	784,36	*
214305_s_at	splicing factor 3b, subunit 1, 155kDa	AW003030	23451	gb:AW003030 /DB_XREF=gi:584	1105,65	48,8	1715,34	31,45	1,55	1,44	1,68	609,69	*
203899_s_at	calcitonin gene-related peptide-receptor co	NM_014478	27297	gb:NM_014478.1 /DB_XREF=gi:7	404,25	21,85	627,29	14,08	1,55	1,41	1,71	223,04	*
46665_at	sema domain, immunoglobulin domain (Ig),	AI949392	54910	Cluster Incl. AI949392:wq11a04.	443,14	14,68	687,54	9,46	1,55	1,46	1,65	244,4	*
223907_s_at	PIN2-interacting protein 1	AF205718	54984	gb:AF205718.1 /DB_XREF=gi:105	304,96	19,1	472,06	12,34	1,55	1,39	1,74	167,1	*
206460_at	transmembrane protein SHREW1	NM_018836	55966	gb:NM_018836.1 /DB_XREF=gi:9	377,52	16,46	584,62	10,63	1,55	1,44	1,68	207,11	*
237333_at	Syncoilin, intermediate filament 1	T90771	81493	gb:T90771 /DB_XREF=gi:722684	447,42	17,7	694,45	11,4	1,55	1,45	1,67	247,03	*
224346_at	gb:AF116671.1 /DB_XREF=gi:7959840 /FEA=	AF116671		gb:AF116671.1 /DB_XREF=gi:795	184,33	9,03	285,96	5,82	1,55	1,43	1,7	101,63	*
202859_x_at	interleukin 8	NM_000584	3576	gb:NM_000584.1 /DB_XREF=gi:1	1034,62	9,12	1589,1	5,93	1,54	1,51	1,56	554,49	*
201831_s_at	vesicle docking protein p115	BE875592	8615	gb:BE875592 /DB_XREF=gi:1032	998,24	18,84	1538,44	12,22	1,54	1,49	1,59	540,2	*
223196_s_at	sestrin 2	AL136551	83667	gb:AL136551.1 /DB_XREF=gi:132	358,79	6,28	551,91	4,08	1,54	1,49	1,59	193,12	*
230188_at	hypothetical protein LOC348938	AW138350	348938	gb:AW138350 /DB_XREF=gi:614	242,24	12,46	373,38	8,08	1,54	1,41	1,69	131,15	*
213926_s_at	HIV-1 Rev binding protein	AI742626	3267	gb:AI742626 /DB_XREF=gi:5110	262,67	9,31	401,53	6,09	1,53	1,44	1,63	138,86	*
215628_x_at	Protein phosphatase 2 (formerly 2A), cataly	AL049285	5515	gb:AL049285.1 /DB_XREF=gi:450	238,53	18,37	364,21	12,03	1,53	1,34	1,77	125,69	*
204611_s_at	protein phosphatase 2, regulatory subunit B	NM_006244	5526	gb:NM_006244.1 /DB_XREF=gi:5	204,68	1,46	313,99	0,95	1,53	1,51	1,55	109,31	*
212332_at	retinoblastoma-like 2 (p130)	BF110947	5934	gb:BF110947 /DB_XREF=gi:1094	236,18	9,02	361,83	5,89	1,53	1,43	1,64	125,64	*
243256_at	MAP kinase interacting serine/threonine kir	AW796364	8569	gb:AW796364 /DB_XREF=gi:784	206,99	6,8	316,77	4,44	1,53	1,44	1,63	109,79	*
203055_s_at	Rho guanine nucleotide exchange factor (GE	NM_004706	9138	gb:NM_004706.1 /DB_XREF=gi:4	355,4	7,12	543,55	4,65	1,53	1,48	1,59	188,15	*
32094_at	carbohydrate (chondroitin 6) sulfotransfera:	AB017915	9469	Cluster Incl. AB017915:Homo sa	519,12	16,35	793,82	10,69	1,53	1,45	1,62	274,7	*
223577_x_at	PRO1073 protein	AA827878	29005	gb:AA827878 /DB_XREF=gi:2900	1394,16	38,72	2132,25	25,32	1,53	1,46	1,61	738,09	*
221501_x_at	hypothetical protein LOC339047	AF229069	339047	gb:AF229069.1 /DB_XREF=gi:898	462,09	13,27	708,55	8,65	1,53	1,46	1,62	246,46	*
239046_at	Transcribed locus	AA322245		gb:AA322245 /DB_XREF=gi:1974	423,67	12,56	648,01	8,21	1,53	1,45	1,61	224,34	*
206562_s_at	casein kinase 1, alpha 1	NM_001892	1452	gb:NM_001892.1 /DB_XREF=gi:4	3154,25	170,1	4800,2	111,77	1,52	1,39	1,68	1645,95	*
213957_s_at	centrosome-associated protein 350	AA635523	9857	gb:AA635523 /DB_XREF=gi:2559	362,39	15,99	551,65	10,5	1,52	1,41	1,65	189,26	*
222439_s_at	thyroid hormone receptor associated protei	BE967048	9967	gb:BE967048 /DB_XREF=gi:1177	2812,98	100,61	4262,67	66,39	1,52	1,42	1,62	1449,69	*
205018_s_at	muscleblind-like 2 (Drosophila)	NM_005757	10150	gb:NM_005757.2 /DB_XREF=gi:9	274,8	9,85	416,55	6,5	1,52	1,42	1,62	141,76	*
208003_s_at	nuclear factor of activated T-cells 5, tonicity	NM_006599	10725	gb:NM_006599.1 /DB_XREF=gi:5	381,83	21,95	581,15	14,42	1,52	1,38	1,69	199,32	*
212553_at	KIAA0460 protein	AK026120	23248	gb:AK026120.1 /DB_XREF=gi:104	693,41	10,26	1050,72	6,77	1,52	1,48	1,56	357,31	*
218030_at	G protein-coupled receptor kinase interacto	NM_014030	28964	gb:NM_014030.1 /DB_XREF=gi:7	1063,76	10,05	1616,5	6,61	1,52	1,49	1,55	552,74	*
219742_at	proline rich 7 (synaptic)	NM_030567	80758	gb:NM_030567.1 /DB_XREF=gi:1	218,88	7,32	333,74	4,8	1,52	1,44	1,62	114,86	*
233543_s_at	hypothetical protein FLJ13614	AK021582	84142	gb:AK021582.1 /DB_XREF=gi:104	204,58	15,24	310,29	10,05	1,52	1,33	1,75	105,71	*
1565641_at	Chromosome 16 open reading frame 45	BE503823	89927	gb:BE503823 /DB_XREF=gi:9706	213,69	7,71	324,64	5,08	1,52	1,43	1,62	110,95	*
241706_at	gb:AA431782 /DB_XREF=gi:2115490 /DB_XI	AA431782		gb:AA431782 /DB_XREF=gi:2115	435,02	4,81	661,42	3,16	1,52	1,49	1,55	226,4	*
239452_at	Transcribed locus	AI088640		gb:AI088640 /DB_XREF=gi:3427	311,4	4,47	473,15	2,94	1,52	1,48	1,56	161,75	*

209661_at	kinesin family member C3	BC001211	3801	gb:BC001211.1 /DB_XREF=gi:12	385,54	5,95	583,84	3,93	1,51	1,47	1,56	198,3	*
209039_x_at	EH-domain containing 1	AF001434	10938	gb:AF001434.1 /DB_XREF=gi:25	622,46	4	937,89	2,66	1,51	1,49	1,52	315,43	*
1558747_at	structural maintenance of chromosomes fle	AA336502	23347	gb:AA336502 /DB_XREF=gi:198	255,98	24,84	385,64	16,49	1,51	1,28	1,81	129,66	*
207253_s_at	ubinuclein 1	NM_016936	29855	gb:NM_016936.1 /DB_XREF=gi:	529,85	6,61	799,3	4,38	1,51	1,48	1,54	269,44	*
222626_at	chromosome 13 open reading frame 10	T79937	64062	gb:T79937 /DB_XREF=gi:698446	314,98	10,66	476,88	7,04	1,51	1,43	1,61	161,9	*
218762_at	zinc finger protein 574	NM_022752	64763	gb:NM_022752.1 /DB_XREF=gi:	201,11	12,33	302,97	8,19	1,51	1,35	1,69	101,86	*
227292_at	hypothetical protein BC007540	AI866590	144097	gb:AI866590 /DB_XREF=gi:5530	751,66	15,73	1135,15	10,42	1,51	1,46	1,57	383,49	*
1558723_at	hypothetical protein LOC284014	AK095567	284014	gb:AK095567.1 /DB_XREF=gi:21	248,55	4,49	374,37	2,98	1,51	1,46	1,56	125,82	*
1555742_at	gb:U92817.1 /DB_XREF=gi:2465327 /TID=H	U92817		gb:U92817.1 /DB_XREF=gi:2465	236,75	14,3	358,24	9,45	1,51	1,36	1,69	121,49	*
213384_x_at	phospholipase C, beta 3 (phosphatidylinosit	Z37544	5331	gb:Z37544 /DB_XREF=gi:609185	818,37	43,35	1226,29	28,93	1,5	1,37	1,65	407,93	*
1569188_s_at	Ribosomal protein L10	BC021806	6134	gb:BC021806.1 /DB_XREF=gi:21	218,61	2,5	327,73	1,67	1,5	1,47	1,53	109,12	*
1558924_s_at	restin (Reed-Steinberg cell-expressed intern	BF673049	6249	gb:BF673049 /DB_XREF=gi:1194	701,61	25,73	1052,67	17,15	1,5	1,41	1,61	351,06	*
202241_at	tribbles homolog 1 (Drosophila)	NM_025195	10221	gb:NM_025195.1 /DB_XREF=gi:	398,99	24,89	598,68	16,59	1,5	1,35	1,69	199,69	*
220511_s_at	deleted in liver cancer 1	NM_024767	10395	gb:NM_024767.1 /DB_XREF=gi:	282,03	1,81	423,15	1,21	1,5	1,48	1,52	141,13	*
223578_x_at	PRO1073 protein	AF113016	29005	gb:AF113016.1 /DB_XREF=gi:66	459,87	25,17	690,97	16,75	1,5	1,37	1,66	231,1	*
227152_at	hypothetical protein FLJ10652	AI979334	55196	gb:AI979334 /DB_XREF=gi:5804	515,04	13,09	772,52	8,73	1,5	1,43	1,57	257,48	*
230629_s_at	E1A binding protein p400	AI809582	57634	gb:AI809582 /DB_XREF=gi:5396	388,43	8,59	584,39	5,71	1,5	1,45	1,57	195,96	*
221705_s_at	hypothetical protein FLJ21168 /// hypotheti	BC005934	80143	gb:BC005934.1 /DB_XREF=gi:13	208,31	3,37	312,27	2,25	1,5	1,46	1,54	103,95	*
221789_x_at	ras homolog gene family, member T2	AK024450	89941	gb:AK024450.1 /DB_XREF=gi:10	225,48	4,87	337,62	3,25	1,5	1,44	1,56	112,14	*
213865_at	discoidin, CUB and LCCL domain containing	AI378788	131566	gb:AI378788 /DB_XREF=gi:4188	726,47	11,44	1086,3	7,65	1,5	1,45	1,54	359,83	*
222252_x_at	gb:AK023354.1 /DB_XREF=gi:10435253 /FE	AK023354		gb:AK023354.1 /DB_XREF=gi:10	1534,24	19,06	2299,3	12,71	1,5	1,47	1,53	765,06	*
203311_s_at	ADP-ribosylation factor 6	M57763	382	gb:M57763.1 /DB_XREF=gi:178	1011,7	19,35	675,38	28,99	-1,5	-1,39	-1,62	-336,32	*
214893_x_at	hyperpolarization activated cyclic nucleotid	AI421964	610	gb:AI421964 /DB_XREF=gi:4267	392,36	0,72	262	1,07	-1,5	-1,49	-1,51	-130,36	*
1555730_a_at	cofilin 1 (non-muscle)	D00682	1072	gb:D00682.1 /DB_XREF=gi:2195	2500,76	16,7	1669,67	25,02	-1,5	-1,46	-1,54	-831,1	*
206635_at	cholinergic receptor, nicotinic, beta polypep	NM_000748	1141	gb:NM_000748.1 /DB_XREF=gi:	374,11	4,5	248,72	6,77	-1,5	-1,43	-1,58	-125,39	*
206284_x_at	clathrin, light polypeptide (Lcb)	NM_001834	1212	gb:NM_001834.1 /DB_XREF=gi:	1108,93	22,96	740,9	34,37	-1,5	-1,38	-1,63	-368,03	*
209126_x_at	keratin 6B	L42612	3854	gb:L42612.1 /DB_XREF=gi:9088	4167,88	22,43	2774,85	33,69	-1,5	-1,47	-1,54	-1393,03	*
204734_at	keratin 15	NM_002275	3866	gb:NM_002275.1 /DB_XREF=gi:	6447,3	72,12	4305,98	107,98	-1,5	-1,43	-1,57	-2141,32	*
227312_at	Syntrophin, beta 2 (dystrophin-associated p	AI694536	6645	gb:AI694536 /DB_XREF=gi:4971	538,57	11,47	359,57	17,19	-1,5	-1,38	-1,64	-179	*
202658_at	peroxisomal biogenesis factor 11B	NM_003846	8799	gb:NM_003846.1 /DB_XREF=gi:	738,5	15,83	491,14	23,8	-1,5	-1,38	-1,65	-247,37	*
236416_at	Rho guanine nucleotide exchange factor (G	AI681617	8874	gb:AI681617 /DB_XREF=gi:4891	362,25	0,75	241,74	1,12	-1,5	-1,49	-1,51	-120,51	*
1554148_a_at	solute carrier family 33 (acetyl-CoA transpo	BC014416	9197	gb:BC014416.1 /DB_XREF=gi:15	630,5	10,16	420,77	15,23	-1,5	-1,41	-1,6	-209,73	*
210288_at	killer cell lectin-like receptor subfamily G, m	AF081675	10219	gb:AF081675.1 /DB_XREF=gi:34	998,23	2,42	667,7	3,62	-1,5	-1,48	-1,51	-330,53	*
218388_at	6-phosphogluconolactonase	NM_012088	25796	gb:NM_012088.1 /DB_XREF=gi:	898,04	16,77	600,32	25,09	-1,5	-1,39	-1,62	-297,72	*
224582_s_at	Nuclear ubiquitous casein kinase and cyclin-	H09085	64710	gb:H09085 /DB_XREF=gi:873907	1120,91	11,44	745,92	17,2	-1,5	-1,44	-1,57	-374,99	*
221477_s_at	hypothetical protein MGC5618	BF575213	79099	gb:BF575213 /DB_XREF=gi:1164	2647,62	5,75	1764,44	8,63	-1,5	-1,49	-1,51	-883,18	*
233380_s_at	RUN and FYVE domain containing 1	AA429145	80230	gb:AA429145 /DB_XREF=gi:2110	425,54	5,5	283,97	8,25	-1,5	-1,42	-1,58	-141,58	*
222278_at	hypothetical LOC389393	AW969655	389393	gb:AW969655 /DB_XREF=gi:815	407,61	0,83	270,91	1,25	-1,5	-1,49	-1,52	-136,7	*
237355_at	Similar to Zgc:85944 protein	AW448981	390468	gb:AW448981 /DB_XREF=gi:698	322,49	0,89	214,99	1,33	-1,5	-1,48	-1,52	-107,5	*
202576_s_at	DEAD (Asp-Glu-Ala-As) box polypeptide 19 /	AL553254	/// 544314 ///	gb:AL553254 /DB_XREF=gi:1289	1748,77	12,15	1167,34	18,2	-1,5	-1,46	-1,54	-581,43	*
228168_at	ATP synthase, H+ transporting, mitochondri	AU153583	518	gb:AU153583 /DB_XREF=gi:1101	403,21	8,59	266,86	12,98	-1,51	-1,39	-1,65	-136,35	*
201971_s_at	ATPase, H+ transporting, lysosomal 70kDa,	NM_001690	523	gb:NM_001690.1 /DB_XREF=gi:	498,31	17,33	330,24	26,15	-1,51	-1,32	-1,75	-168,07	*
205595_at	desmoglein 3 (pemphigus vulgaris antigen)	NM_001944	1830	gb:NM_001944.1 /DB_XREF=gi:	857,89	11,06	566,28	16,75	-1,51	-1,44	-1,6	-291,61	*
202363_at	sparc/osteonectin, cwcv and kazal-like dom	AF231124	6695	gb:AF231124.1 /DB_XREF=gi:72	629,7	6,38	417,89	9,62	-1,51	-1,45	-1,57	-211,81	*
1558254_s_at	SFRS protein kinase 2	BU155802	6733	gb:BU155802 /DB_XREF=gi:226	340,78	4,6	225,78	6,95	-1,51	-1,43	-1,6	-115,01	*
219924_s_at	zinc finger protein 258	NM_007167	9204	gb:NM_007167.1 /DB_XREF=gi:	889,09	10,59	589,02	15,99	-1,51	-1,44	-1,59	-300,07	*
214553_s_at	cyclic AMP phosphoprotein, 19 kD	NM_006628	10776	gb:NM_006628.1 /DB_XREF=gi:	478,5	9,99	317,79	15,04	-1,51	-1,39	-1,64	-160,71	*
231771_at	gap junction protein, beta 6 (connexin 30)	AI694073	10804	gb:AI694073 /DB_XREF=gi:4971	496,33	2,05	329,16	3,1	-1,51	-1,48	-1,53	-167,17	*
213935_at	abhydrolase domain containing 5	AF007132	51099	gb:AF007132.1 /DB_XREF=gi:28	323,92	3,74	214,01	5,65	-1,51	-1,44	-1,59	-109,9	*
222531_s_at	chromosome 14 open reading frame 108	AW137526	55745	gb:AW137526 /DB_XREF=gi:614	1430,28	34,47	947,89	52,01	-1,51	-1,37	-1,67	-482,39	*

218752_at	U11/U12 snRNP 20K	NM_019103	55954	gb:NM_019103.1 /DB_XREF=gi:9	517,18	8,24	342,99	12,42	-1,51	-1,41	-1,61	-174,19	*
223735_at	ADP-ribosylation factor-like 6	AL136815	84100	gb:AL136815.1 /DB_XREF=gi:12	384,04	4,05	255,07	6,1	-1,51	-1,44	-1,57	-128,98	*
227835_at	Hypothetical gene supported by AL713796	T86830	389831	gb:T86830 /DB_XREF=gi:715182	389,11	6,93	256,97	10,5	-1,51	-1,41	-1,63	-132,14	*
204645_at	cyclin T2	NM_001241	905	gb:NM_001241.1 /DB_XREF=gi:4	391,79	10,54	258,42	15,98	-1,52	-1,36	-1,7	-133,37	*
204305_at	mitochondrial intermediate peptidase	NM_005932	4285	gb:NM_005932.1 /DB_XREF=gi:5	763,09	27,2	502,25	41,32	-1,52	-1,32	-1,78	-260,83	*
213465_s_at	protein phosphatase 1, regulatory subunit 7	BF718769	5510	gb:BF718769 /DB_XREF=gi:1201	1602,18	6,09	1051,2	9,29	-1,52	-1,5	-1,55	-550,98	*
216899_s_at	src family associated phosphoprotein 2	AC003999	8935	gb:AC003999 /DB_XREF=gi:2772	407,77	4,74	268,2	7,2	-1,52	-1,45	-1,6	-139,57	*
210852_s_at	aminoadipate-semialdehyde synthase	AF229180	10157	gb:AF229180.1 /DB_XREF=gi:72	363,49	5,7	238,99	8,66	-1,52	-1,43	-1,63	-124,5	*
201264_at	coatomer protein complex, subunit epsilon	NM_007263	11316	gb:NM_007263.1 /DB_XREF=gi:6	396,64	9	260,3	13,72	-1,52	-1,39	-1,68	-136,34	*
214449_s_at	ras homolog gene family, member Q	NM_012249	23433	gb:NM_012249.1 /DB_XREF=gi:6	607,36	8,07	400,75	12,23	-1,52	-1,44	-1,6	-206,61	*
214581_x_at	tumor necrosis factor receptor superfamily,	BE568134	27242	gb:BE568134 /DB_XREF=gi:9811	566,61	3,4	371,89	5,18	-1,52	-1,49	-1,56	-194,72	*
212032_s_at	prostate tumor overexpressed gene 1	AL046054	53635	gb:AL046054 /DB_XREF=gi:5936	542,77	7,59	357,53	11,52	-1,52	-1,43	-1,61	-185,24	*
221781_s_at	DnaJ (Hsp40) homolog, subfamily C, membe	BG168666	54431	gb:BG168666 /DB_XREF=gi:1267	614,5	6,47	403,21	9,85	-1,52	-1,46	-1,59	-211,29	*
228397_at	Hypothetical protein FLJ20618	AW082208	55000	gb:AW082208 /DB_XREF=gi:603	377,18	5,09	248,51	7,72	-1,52	-1,44	-1,61	-128,68	*
230943_at	SRY (sex determining region Y)-box 17	AI821669	64321	gb:AI821669 /DB_XREF=gi:5440	297,66	2,24	196,11	3,4	-1,52	-1,47	-1,57	-101,55	*
228536_at	hypothetical protein BC004337	AA574240	90826	gb:AA574240 /DB_XREF=gi:2348	385,53	4,36	253	6,64	-1,52	-1,45	-1,6	-132,53	*
203442_x_at	hypothetical protein FLJ35827	AA478965	256364	gb:AA478965 /DB_XREF=gi:2207	448,3	5,07	295,37	7,7	-1,52	-1,45	-1,59	-152,93	*
225062_at	hypothetical gene supported by AL713796	BF667120	389831	gb:BF667120 /DB_XREF=gi:1194	595,34	9,89	392,77	14,99	-1,52	-1,42	-1,63	-202,57	*
231975_s_at	gb:AK002183.1 /DB_XREF=gi:7023905 /FEA	AK002183		gb:AK002183.1 /DB_XREF=gi:70	369,96	5,66	243,86	8,59	-1,52	-1,43	-1,62	-126,09	*
214578_s_at	gb:AV683882 /DB_XREF=gi:10285745 /DB_	AV683882		gb:AV683882 /DB_XREF=gi:1028	1412,66	20,38	927,65	31,04	-1,52	-1,44	-1,62	-485,01	*
1553551_s_at	gb:NM_173709.1 /DB_XREF=gi:27754201 /(	NM_173709		gb:NM_173709.1 /DB_XREF=gi:2	10209,61	88,49	6725,65	134,33	-1,52	-1,47	-1,57	-3483,96	*
201850_at	capping protein (actin filament), gelsolin-like	NM_001747	822	gb:NM_001747.1 /DB_XREF=gi:4	1600,5	21,89	1046,43	33,48	-1,53	-1,45	-1,62	-554,07	*
213581_at	programmed cell death 2	BF446180	5134	gb:BF446180 /DB_XREF=gi:1151	1115,84	13,28	727,07	20,38	-1,53	-1,46	-1,61	-388,77	*
203329_at	protein tyrosine phosphatase, receptor type	NM_002845	5797	gb:NM_002845.1 /DB_XREF=gi:4	355,01	2,54	231,61	3,89	-1,53	-1,49	-1,58	-123,4	*
227164_at	Splicing factor, arginine/serine-rich 1 (splicir	AL521786	6426	gb:AL521786 /DB_XREF=gi:1278	504,96	1,57	329,75	2,41	-1,53	-1,51	-1,55	-175,2	*
214168_s_at	tight junction protein 1 (zona occludens 1)	AA813018	7082	gb:AA813018 /DB_XREF=gi:2883	599,37	12,31	392,13	18,82	-1,53	-1,41	-1,67	-207,24	*
226268_at	RAB21, member RAS oncogene family	AI309554	23011	gb:AI309554 /DB_XREF=gi:4004	521,75	5,5	341,88	8,4	-1,53	-1,46	-1,6	-179,87	*
219201_s_at	twisted gastrulation homolog 1 (Drosophila)	NM_020648	57045	gb:NM_020648.1 /DB_XREF=gi:1	477,13	6,97	312,44	10,65	-1,53	-1,44	-1,63	-164,69	*
231867_at	odz, odd Oz/ten-m homolog 2 (Drosophila)	AB032953	57451	gb:AB032953.1 /DB_XREF=gi:63	678,48	10,79	442,05	16,56	-1,53	-1,44	-1,64	-236,42	*
219469_at	dynein, cytoplasmic, heavy polypeptide 2	NM_024606	79659	gb:NM_024606.1 /DB_XREF=gi:1	599,28	8,93	391,2	13,69	-1,53	-1,44	-1,63	-208,09	*
226506_at	hypothetical protein FLJ13710	AI742570	79875	gb:AI742570 /DB_XREF=gi:5110	329,16	8,22	214,91	12,58	-1,53	-1,38	-1,71	-114,25	*
238863_x_at	Transcribed locus, weakly similar to NP_055	AI524240		gb:AI524240 /DB_XREF=gi:4438	758,01	6,94	495,23	10,62	-1,53	-1,47	-1,59	-262,78	*
209275_s_at	ceroid-lipofuscinosis, neuronal 3, juvenile (E	AF015593	1201	gb:AF015593.1 /DB_XREF=gi:41	290,41	4,5	188,43	6,93	-1,54	-1,45	-1,65	-101,99	*
242363_at	Dynein, cytoplasmic, intermediate polypept	BF592008	1781	gb:BF592008 /DB_XREF=gi:1168	580,55	21,22	375,77	32,78	-1,54	-1,33	-1,82	-204,79	*
205770_at	glutathione reductase	NM_000637	2936	gb:NM_000637.1 /DB_XREF=gi:1	811,68	10,38	526,54	16,01	-1,54	-1,46	-1,63	-285,14	*
214359_s_at	heat shock 90kDa protein 1, beta	AI218219	3326	gb:AI218219 /DB_XREF=gi:3798	2823,84	30,58	1836,02	47,04	-1,54	-1,47	-1,61	-987,82	*
213680_at	keratin 6B	AI831452	3854	gb:AI831452 /DB_XREF=gi:5452	880,63	10,29	573,03	15,82	-1,54	-1,46	-1,62	-307,6	*
203466_at	MpV17 transgene, murine homolog, glomer	NM_002437	4358	gb:NM_002437.1 /DB_XREF=gi:4	677,44	7,46	440,31	11,48	-1,54	-1,47	-1,61	-237,13	*
217457_s_at	RAP1, GTP-GDP dissociation stimulator 1	X63465	5910	gb:X63465.1 /DB_XREF=gi:3207	773,84	1,47	501,95	2,27	-1,54	-1,53	-1,55	-271,89	*
201006_at	peroxiredoxin 2	NM_005809	7001	gb:NM_005809.1 /DB_XREF=gi:5	548,59	4,7	356,49	7,24	-1,54	-1,48	-1,6	-192,09	*
212041_at	ATPase, H+ transporting, lysosomal 38kDa, V	AL566172	9114	gb:AL566172 /DB_XREF=gi:1291	1296,04	26,56	843,8	40,8	-1,54	-1,41	-1,68	-452,25	*
217847_s_at	thyroid hormone receptor associated protei	NM_005119	9967	gb:NM_005119.1 /DB_XREF=gi:4	380,11	5,99	246,61	9,24	-1,54	-1,44	-1,65	-133,49	*
216071_x_at	mediator of RNA polymerase II transcription	AF132033	9968	gb:AF132033 /DB_XREF=gi:5524	1659,53	5,91	1076,25	9,11	-1,54	-1,52	-1,57	-583,27	*
202955_s_at	ADP-ribosylation factor guanine nucleotide-	AF084520	10565	gb:AF084520.1 /DB_XREF=gi:50	337,03	1,79	218,63	2,76	-1,54	-1,51	-1,58	-118,4	*
217188_s_at	chromosome 14 open reading frame 1	AC007182	11161	gb:AC007182 /DB_XREF=gi:5708	687,55	8,96	447,24	13,77	-1,54	-1,46	-1,63	-240,31	*
222478_at	chromosome 13 open reading frame 9	AL576924	51028	gb:AL576924 /DB_XREF=gi:1293	1214,1	13,73	786,01	21,21	-1,54	-1,47	-1,62	-428,09	*
226888_at	Casein kinase 1, gamma 1	BG104860	53944	gb:BG104860 /DB_XREF=gi:1259	564,99	9,67	366,12	14,92	-1,54	-1,44	-1,66	-198,86	*
218437_s_at	leucine zipper transcription factor-like 1	NM_020347	54585	gb:NM_020347.1 /DB_XREF=gi:9	299,84	1,5	194,63	2,31	-1,54	-1,51	-1,57	-105,21	*
219527_at	hypothetical protein FLJ20605	NM_017898	54996	gb:NM_017898.1 /DB_XREF=gi:8	293,97	3,34	191,51	5,12	-1,54	-1,46	-1,61	-102,47	*
1554679_a_at	lysosomal associated protein transmembrar	AF317417	55353	gb:AF317417.1 /DB_XREF=gi:14	981,94	15,97	638,04	24,57	-1,54	-1,44	-1,65	-343,91	*

218584_at	hypothetical protein FLJ21127	NM_024549	79600	gb:NM_024549.1 /DB_XREF=gi:1	380,62	5,36	246,47	8,28	-1,54	-1,46	-1,64	-134,15	*
234945_at	family with sequence similarity 54, member	AL138828	113115	gb:AL138828 /DB_XREF=gi:1044	379,91	4,34	247,41	6,66	-1,54	-1,46	-1,61	-132,5	*
225328_at	F-box protein 32	N21643	114907	gb:N21643 /DB_XREF=gi:112681	344,57	1,05	224,38	1,61	-1,54	-1,52	-1,56	-120,19	*
227270_at	hypothetical protein LOC285550	BF593037	285550	gb:BF593037 /DB_XREF=gi:1168	354,65	4,58	229,95	7,06	-1,54	-1,46	-1,63	-124,7	*
238656_at	Transcribed locus, strongly similar to XP_51	AA877043		gb:AA877043 /DB_XREF=gi:2986	325,59	7,53	211,86	11,58	-1,54	-1,4	-1,7	-113,73	*
211558_s_at	deoxyhypusine synthase	U26266	1725	gb:U26266.1 /DB_XREF=gi:1373	297,17	7,11	191,16	11,06	-1,55	-1,41	-1,73	-106,01	*
210233_at	interleukin 1 receptor accessory protein	AF167343	3556	gb:AF167343.1 /DB_XREF=gi:80	328,29	6,17	212,43	9,53	-1,55	-1,43	-1,68	-115,85	*
201234_at	integrin-linked kinase	NM_004517	3611	gb:NM_004517.1 /DB_XREF=gi:4	847,8	12,04	548,32	18,62	-1,55	-1,46	-1,64	-299,48	*
206544_x_at	SWI/SNF related, matrix associated, actin de	NM_003070	6595	gb:NM_003070.1 /DB_XREF=gi:4	607,64	6,38	391,37	9,91	-1,55	-1,49	-1,63	-216,27	*
216697_at	Triple functional domain (PTPRF interacting)	AL161955	7204	gb:AL161955.1 /DB_XREF=gi:73	1022,7	1,54	660,24	2,39	-1,55	-1,54	-1,56	-362,46	*
217799_x_at	ubiquitin-conjugating enzyme E2H (UBC8 ho	NM_003344	7328	gb:NM_003344.1 /DB_XREF=gi:4	1629,11	25,51	1050,68	39,55	-1,55	-1,45	-1,66	-578,42	*
211238_at	a disintegrin and metalloproteinase domain	AF215824	8756	gb:AF215824.1 /DB_XREF=gi:12	1762,71	5,85	1140,49	9,04	-1,55	-1,52	-1,57	-622,22	*
203404_at	armadillo repeat containing, X-linked 2	NM_014782	9823	gb:NM_014782.1 /DB_XREF=gi:7	363,7	4,24	235,06	6,57	-1,55	-1,47	-1,63	-128,64	*
1554352_s_at	c-myc promoter binding protein	BC041706	10260	gb:BC041706.1 /DB_XREF=gi:27	492,38	5,31	316,91	8,26	-1,55	-1,48	-1,63	-175,47	*
202784_s_at	nicotinamide nucleotide transhydrogenase	NM_012343	23530	gb:NM_012343.1 /DB_XREF=gi:6	605,3	5,15	389,8	7,99	-1,55	-1,5	-1,61	-215,49	*
218036_x_at	NMD3 homolog (S. cerevisiae)	NM_015938	51068	gb:NM_015938.1 /DB_XREF=gi:7	941,35	18,44	606,5	28,62	-1,55	-1,43	-1,69	-334,84	*
211376_s_at	chromosome 10 open reading frame 86	BC005212	54780	gb:BC005212.1 /DB_XREF=gi:13	1214,43	29,66	784,22	45,93	-1,55	-1,4	-1,73	-430,21	*
224373_s_at	6-pyruvoyl-tetrahydropterin synthase/dime	AF253979	84105	gb:AF253979.1 /DB_XREF=gi:12	16477,91	148,17	10602,71	230,28	-1,55	-1,5	-1,62	-5875,2	*
1556429_a_at	unknown MGC21654 product	BC026969	93594	gb:BC026969.1 /DB_XREF=gi:20	309,64	4,65	199,28	7,23	-1,55	-1,46	-1,66	-110,36	*
227119_at	CCR4-NOT transcription complex, subunit 6-	BF103856	246175	gb:BF103856 /DB_XREF=gi:1088	393,01	4,84	253,34	7,5	-1,55	-1,47	-1,64	-139,67	*
212969_x_at	hypothetical protein FLJ35827	BE222618	256364	gb:BE222618 /DB_XREF=gi:8909	434,33	3,88	279,7	6,03	-1,55	-1,5	-1,61	-154,63	*
223443_s_at	hypothetical protein LOC51321 /// hypothe	BC003669	01283 /// 5132	gb:BC003669.1 /DB_XREF=gi:13	560,48	4,06	362,26	6,29	-1,55	-1,5	-1,6	-198,22	*
216383_at	ribosomal protein L18a /// similar to riboso	U52111	47544 /// 614	gb:U52111 /DB_XREF=gi:833175	465,48	7,2	299,95	11,17	-1,55	-1,45	-1,66	-165,53	*
205750_at	biphenyl hydrolase-like (serine hydrolase; b	NM_004332	670	gb:NM_004332.1 /DB_XREF=gi:4	681,1	18,76	435,65	29,34	-1,56	-1,39	-1,77	-245,46	*
205196_s_at	adaptor-related protein complex 1, sigma 1	NM_001283	1174	gb:NM_001283.1 /DB_XREF=gi:4	633,26	11,69	406,33	18,22	-1,56	-1,44	-1,69	-226,93	*
227677_at	Janus kinase 3 (a protein tyrosine kinase, le	BF512748	3718	gb:BF512748 /DB_XREF=gi:1159	522,59	3,83	335,21	5,97	-1,56	-1,51	-1,61	-187,38	*
210102_at	loss of heterozygosity, 11, chromosomal reg	BC001234	4013	gb:BC001234.1 /DB_XREF=gi:12	493,11	10,61	315,94	16,57	-1,56	-1,43	-1,72	-177,17	*
212016_s_at	polypyrimidine tract binding protein 1	AA679988	5725	gb:AA679988 /DB_XREF=gi:2656	484,39	3,65	311,22	5,69	-1,56	-1,51	-1,61	-173,16	*
211536_x_at	mitogen-activated protein kinase kinase kin	AB009358	6885	gb:AB009358.2 /DB_XREF=gi:89	340,12	10,63	217,87	16,59	-1,56	-1,37	-1,8	-122,25	*
242879_x_at	V-akt murine thymoma viral oncogene hom	AI939442	10000	gb:AI939442 /DB_XREF=gi:5678	302,34	3,78	193,93	5,9	-1,56	-1,48	-1,65	-108,42	*
230926_s_at	KIAA1229 protein	AW452022	57489	gb:AW452022 /DB_XREF=gi:699	308,04	3,69	197,42	5,76	-1,56	-1,48	-1,65	-110,62	*
225631_at	KIAA1706 protein	BE501865	80820	gb:BE501865 /DB_XREF=gi:9704	494,59	3,7	317,21	5,77	-1,56	-1,51	-1,61	-177,38	*
1553929_at	N-acylsphingosine amidohydrolase (alkaline	NM_133492	125981	gb:NM_133492.1 /DB_XREF=gi:1	1737,15	4,07	1114,84	6,35	-1,56	-1,54	-1,57	-622,31	*
208596_s_at	UDP glycosyltransferase 1 family, polypepti	NM_019093	/// 54579 ///	gb:NM_019093.1 /DB_XREF=gi:1	436,2	13	279,2	20,3	-1,56	-1,38	-1,79	-157	*
206042_x_at	small nuclear ribonucleoprotein polypeptid	NM_022804	6638 /// 8926	gb:NM_022804.1 /DB_XREF=gi:1	722,59	9,99	463,29	15,59	-1,56	-1,47	-1,66	-259,3	*
239973_at	Full-length cDNA clone CS0DJ013YE21 of T c	AW450675		gb:AW450675 /DB_XREF=gi:699	466,75	2,07	298,67	3,23	-1,56	-1,53	-1,59	-168,08	*
1555852_at	gb:AI375915 /DB_XREF=gi:4175905 /DB_XR	AI375915		gb:AI375915 /DB_XREF=gi:4175	383,31	6,08	245,87	9,49	-1,56	-1,46	-1,67	-137,44	*
215719_x_at	Fas (TNF receptor superfamily, member 6)	X83493	355	gb:X83493.1 /DB_XREF=gi:9714	852,85	3,91	544,57	6,13	-1,57	-1,54	-1,6	-308,28	*
225805_at	Heterogeneous nuclear ribonucleoprotein U	AW137669	3192	gb:AW137669 /DB_XREF=gi:614	625,69	10,61	398,83	16,64	-1,57	-1,46	-1,69	-226,85	*
201604_s_at	protein phosphatase 1, regulatory (inhibitor	NM_002480	4659	gb:NM_002480.1 /DB_XREF=gi:4	1446,95	10,4	922,04	16,33	-1,57	-1,52	-1,62	-524,91	*
218129_s_at	nuclear transcription factor Y, beta	NM_006166	4801	gb:NM_006166.2 /DB_XREF=gi:1	751,03	15,48	477,56	24,35	-1,57	-1,44	-1,73	-273,46	*
217356_s_at	phosphoglycerate kinase 1	S81916	5230	gb:S81916.1 /DB_XREF=gi:1470	5680,24	24,07	3623,67	37,72	-1,57	-1,54	-1,6	-2056,56	*
216175_at	Polymerase (DNA directed), delta 2, regulat	AK025276	5425	gb:AK025276.1 /DB_XREF=gi:10	276,3	1,61	176,2	2,53	-1,57	-1,53	-1,61	-100,1	*
203210_s_at	replication factor C (activator 1) 5, 36.5kDa	NM_007370	5985	gb:NM_007370.1 /DB_XREF=gi:6	544,19	12,62	345,86	19,85	-1,57	-1,43	-1,75	-198,33	*
210372_s_at	tumor protein D52-like 1	AF208012	7164	gb:AF208012.1 /DB_XREF=gi:12	896,02	16,45	569,29	25,89	-1,57	-1,46	-1,71	-326,73	*
201813_s_at	TBC1 domain family, member 5	AI654161	9779	gb:AI654161 /DB_XREF=gi:4738	399,14	7,09	254,45	11,13	-1,57	-1,45	-1,7	-144,7	*
209316_s_at	HBS1-like (S. cerevisiae)	BC001465	10767	gb:BC001465.1 /DB_XREF=gi:12	2448,85	15,61	1562,14	24,47	-1,57	-1,53	-1,61	-886,71	*
223400_s_at	polybromo 1	AF197569	55193	gb:AF197569.1 /DB_XREF=gi:11	305,11	6,53	193,79	10,28	-1,57	-1,44	-1,74	-111,32	*
214941_s_at	formin binding protein 3	AA675900	55660	gb:AA675900 /DB_XREF=gi:2775	1282,77	12	817,43	18,83	-1,57	-1,51	-1,64	-465,33	*
213312_at	chromosome 6 open reading frame 162	NM_020425	57150	gb:NM_020425.1 /DB_XREF=gi:1	451,86	11,74	287,57	18,45	-1,57	-1,41	-1,77	-164,28	*

228429_x_at	kinesin family member 9	BG168764	64147	gb:BG168764 /DB_XREF=gi:1267	478,54	2,61	305,02	4,1	-1,57	-1,53	-1,61	-173,51	*
1553708_at	hypothetical protein MGC16075	NM_032761	84847	gb:NM_032761.1 /DB_XREF=gi:1	495,92	9,24	315,63	14,52	-1,57	-1,45	-1,71	-180,29	*
1557055_s_at	hypothetical protein LOC284591	BC042880	284591	gb:BC042880.1 /DB_XREF=gi:27!	700,67	16,89	446,49	26,5	-1,57	-1,42	-1,75	-254,18	*
208229_at	gb:NM_022975.1 /DB_XREF=gi:13186264 /(	NM_022975		gb:NM_022975.1 /DB_XREF=gi:1	287,59	3,54	183,24	5,55	-1,57	-1,49	-1,66	-104,35	*
1554241_at	coagulation factor C homolog, cochlin (Limu	BC007230	1690	gb:BC007230.1 /DB_XREF=gi:13!	1275,38	8,18	809,06	12,89	-1,58	-1,53	-1,62	-466,32	*
207590_s_at	FSH primary response (LRPR1 homolog, rat)	NM_006733	2491	gb:NM_006733.1 /DB_XREF=gi:5	593,65	6,52	375,11	10,32	-1,58	-1,51	-1,66	-218,54	*
204867_at	GTP cyclohydrolase I feedback regulator	NM_005258	2644	gb:NM_005258.2 /DB_XREF=gi:6	287,78	1,3	181,58	2,06	-1,58	-1,55	-1,62	-106,2	*
205157_s_at	keratin 17	NM_000422	3872	gb:NM_000422.1 /DB_XREF=gi:4	5296,25	84,63	3350	133,79	-1,58	-1,48	-1,7	-1946,25	*
212741_at	monoamine oxidase A	AA923354	4128	gb:AA923354 /DB_XREF=gi:3070	1687,9	14,09	1067,07	22,28	-1,58	-1,53	-1,64	-620,83	*
203226_s_at	sarcoma amplified sequence	AL514076	6302	gb:AL514076 /DB_XREF=gi:1277	459,92	11,81	291,97	18,6	-1,58	-1,41	-1,77	-167,95	*
201527_at	ATPase, H+ transporting, lysosomal 14kDa, \	NM_004231	9296	gb:NM_004231.1 /DB_XREF=gi:4	1503,16	30,57	951,41	48,29	-1,58	-1,45	-1,73	-551,75	*
225294_s_at	trafficking protein particle complex 1	BG340967	58485	gb:BG340967 /DB_XREF=gi:1314	324,82	10,77	205,15	17,05	-1,58	-1,38	-1,85	-119,68	*
221573_at	chromosome 7 open reading frame 25	BC000769	79020	gb:BC000769.1 /DB_XREF=gi:12(	281,59	5,11	178,58	8,06	-1,58	-1,46	-1,71	-103,01	*
222393_s_at	Mak3 homolog (S. cerevisiae)	AU149868	80218	gb:AU149868 /DB_XREF=gi:1101	2255,57	27,22	1424,4	43,1	-1,58	-1,5	-1,67	-831,17	*
1554489_a_at	p10-binding protein	BC016050	80321	gb:BC016050.1 /DB_XREF=gi:16:	860,23	12,86	544,11	20,33	-1,58	-1,48	-1,69	-316,13	*
1552486_s_at	lactamase, beta	NM_171846	114294	gb:NM_171846.1 /DB_XREF=gi:2	420,82	5,78	267,16	9,1	-1,58	-1,48	-1,68	-153,66	*
235433_at	chromosome X open reading frame 33	BG290639	139322	gb:BG290639 /DB_XREF=gi:1304	373,47	6,82	236,23	10,78	-1,58	-1,46	-1,72	-137,24	*
208310_s_at	chromosome 7 open reading frame 28A ///	NM_007085	21960 /// 5162	gb:NM_007085.1 /DB_XREF=gi:5	1505,04	18,46	952,3	29,18	-1,58	-1,5	-1,67	-552,73	*
209805_at	PMS2 postmeiotic segregation increased 2 (	U14658	41194 /// 539	gb:U14658.1 /DB_XREF=gi:5574	366,59	3,74	232,18	5,91	-1,58	-1,51	-1,65	-134,42	*
235917_at	Transcribed locus	AI950319		gb:AI950319 /DB_XREF=gi:5742(	416,41	2,61	263,39	4,12	-1,58	-1,54	-1,63	-153,01	*
201122_x_at	eukaryotic translation initiation factor 5A	BC000751	1984	gb:BC000751.1 /DB_XREF=gi:12(	5180,79	34,01	3254,16	54,15	-1,59	-1,55	-1,64	-1926,63	*
210904_s_at	interleukin 13 receptor, alpha 1	U81380	3597	gb:U81380.2 /DB_XREF=gi:5174	599,76	12,09	377,93	19,19	-1,59	-1,45	-1,74	-221,83	*
203417_at	microfibrillar-associated protein 2	NM_017459	4237	gb:NM_017459.1 /DB_XREF=gi:9	594,64	5,12	374,19	8,13	-1,59	-1,53	-1,65	-220,46	*
201476_s_at	ribonucleotide reductase M1 polypeptide	AI692974	6240	gb:AI692974 /DB_XREF=gi:4970:	653,73	17,22	410,21	27,44	-1,59	-1,42	-1,8	-243,52	*
216591_s_at	succinate dehydrogenase complex, subunit	AF080579	6391	gb:AF080579 /DB_XREF=gi:3406	1279,11	11,87	804,24	18,87	-1,59	-1,53	-1,66	-474,87	*
210317_s_at	tyrosine 3-monooxygenase/tryptophan 5-m	U28936	7531	gb:U28936.1 /DB_XREF=gi:9843	610,12	6	383,8	9,54	-1,59	-1,52	-1,66	-226,33	*
216266_s_at	ADP-ribosylation factor guanine nucleotide-	AK025637	10565	gb:AK025637.1 /DB_XREF=gi:10	478,38	6,67	300,38	10,62	-1,59	-1,5	-1,7	-178	*
210028_s_at	origin recognition complex, subunit 3-like (y	AF125507	23595	gb:AF125507.1 /DB_XREF=gi:43:	2734,82	9,9	1721,91	15,73	-1,59	-1,56	-1,61	-1012,91	*
223649_s_at	CGI-69 protein	AF317711	51629	gb:AF317711.1 /DB_XREF=gi:12(	1134,36	10,26	712,16	16,34	-1,59	-1,53	-1,66	-422,19	*
218787_x_at	CWF19-like 1, cell cycle control (S. pombe)	NM_018294	55280	gb:NM_018294.1 /DB_XREF=gi:8	464,27	3,11	292,08	4,94	-1,59	-1,54	-1,64	-172,19	*
1555334_s_at	solute carrier family 30 (zinc transporter), m	AF439324	64924	gb:AF439324.1 /DB_XREF=gi:19(	487,44	9,41	306,04	14,98	-1,59	-1,46	-1,74	-181,41	*
225659_at	hypothetical protein LOC339745	BF516590	339745	gb:BF516590 /DB_XREF=gi:1160	414,94	3,03	261,44	4,81	-1,59	-1,54	-1,64	-153,51	*
228791_at	Hypothetical protein MGC61550	BF434655	348110	gb:BF434655 /DB_XREF=gi:1144	384,56	11,57	242,39	18,36	-1,59	-1,4	-1,83	-142,17	*
229227_at	FLJ45244 protein	BF511219	400242	gb:BF511219 /DB_XREF=gi:1159	378,06	4,06	237,92	6,45	-1,59	-1,52	-1,67	-140,14	*
233732_at	MRNA; cDNA DKFZp566O134 (from clone D	AL137445		gb:AL137445.1 /DB_XREF=gi:68(	704,26	0,71	443,91	1,12	-1,59	-1,58	-1,59	-260,35	*
214505_s_at	four and a half LIM domains 1	AF220153	2273	gb:AF220153.1 /DB_XREF=gi:69	402,66	4,3	251,29	6,89	-1,6	-1,53	-1,68	-151,37	*
202592_at	biogenesis of lysosome-related organelles c	NM_001487	2647	gb:NM_001487.1 /DB_XREF=gi:4	473,76	11,4	296,43	18,22	-1,6	-1,44	-1,79	-177,33	*
201644_at	tissue specific transplantation antigen P35B	NM_003313	7264	gb:NM_003313.2 /DB_XREF=gi:6	923,93	6,04	578,13	9,65	-1,6	-1,55	-1,65	-345,79	*
204453_at	zinc finger protein 84 (HPF2)	NM_003428	7637	gb:NM_003428.1 /DB_XREF=gi:4	309,28	5,85	193,19	9,36	-1,6	-1,47	-1,75	-116,08	*
204871_at	transcription termination factor, mitochond	NM_006980	7978	gb:NM_006980.1 /DB_XREF=gi:5	496,89	5,74	310,75	9,17	-1,6	-1,52	-1,69	-186,14	*
208811_s_at	DnaJ (Hsp40) homolog, subfamily B, membe	AF080569	10049	gb:AF080569.1 /DB_XREF=gi:43:	2000,36	24,96	1251,73	39,89	-1,6	-1,51	-1,69	-748,63	*
1555167_s_at	pre-B-cell colony enhancing factor 1	BC020691	10135	gb:BC020691.1 /DB_XREF=gi:18(	342,95	7,26	214,82	11,58	-1,6	-1,46	-1,76	-128,14	*
242916_at	centrosomal protein 1	AA642477	11064	gb:AA642477 /DB_XREF=gi:2567	345,19	1,32	215,41	2,11	-1,6	-1,58	-1,63	-129,78	*
214507_s_at	exosome component 2	NM_014285	23404	gb:NM_014285.1 /DB_XREF=gi:7	437,11	9,92	272,97	15,88	-1,6	-1,45	-1,78	-164,14	*
219260_s_at	S-phase 2 protein	NM_015362	23587	gb:NM_015362.1 /DB_XREF=gi:7	519,59	5,3	325,32	8,47	-1,6	-1,53	-1,67	-194,27	*
228091_at	Syntaxin 17	AI800609	55014	gb:AI800609 /DB_XREF=gi:5366:	714,32	5,78	446,28	9,25	-1,6	-1,54	-1,66	-268,05	*
221447_s_at	glycosyltransferase 8 domain containing 2 /,	NM_031302	83468	gb:NM_031302.1 /DB_XREF=gi:1	353,72	9,62	221,42	15,37	-1,6	-1,42	-1,82	-132,3	*
1570523_s_at	APG10 autophagy 10-like (S. cerevisiae)	AF318326	83734	gb:AF318326.1 /DB_XREF=gi:18(	424,93	6,43	265,01	10,31	-1,6	-1,5	-1,72	-159,93	*
212891_s_at	growth arrest and DNA-damage-inducible, g	BF972185	90480	gb:BF972185 /DB_XREF=gi:1233	1673,41	3,43	1048,46	5,47	-1,6	-1,58	-1,61	-624,95	*
225832_s_at	KCCR13L	BE795104	221955	gb:BE795104 /DB_XREF=gi:1021	304,02	6,14	189,85	9,83	-1,6	-1,47	-1,76	-114,17	*

222987_s_at	transmembrane protein 9	NM_016456	252839	gb:NM_016456.1 /DB_XREF=gi:7	1250,97	8,32	781,74	13,31	-1,6	-1,55	-1,65	-469,23	*
225200_at	zinc finger, CSL domain containing 2	AU151106	285381	gb:AU151106 /DB_XREF=gi:1101	486,64	8,22	303,23	13,2	-1,6	-1,49	-1,74	-183,41	*
216918_s_at	dystonin	AL096710	667	gb:AL096710 /DB_XREF=gi:5738	1539,89	20,62	955,32	33,24	-1,61	-1,52	-1,72	-584,57	*
226160_at	hexose-6-phosphate dehydrogenase (glucos	AW138757	9563	gb:AW138757 /DB_XREF=gi:614	273,78	4,8	170,52	7,7	-1,61	-1,49	-1,74	-103,26	*
200988_s_at	proteasome (prosome, macropain) activato	NM_005789	10197	gb:NM_005789.1 /DB_XREF=gi:5	632,24	9,35	392,84	15,05	-1,61	-1,51	-1,72	-239,4	*
203779_s_at	epithelial V-like antigen 1	NM_005797	10205	gb:NM_005797.1 /DB_XREF=gi:5	311,37	3,74	193,83	6	-1,61	-1,52	-1,7	-117,54	*
220773_s_at	gephyrin	NM_020806	10243	gb:NM_020806.1 /DB_XREF=gi:1	406,75	8,06	252,44	12,99	-1,61	-1,48	-1,77	-154,31	*
218382_s_at	U2 (RNU2) small nuclear RNA auxiliary facto	NM_007279	11338	gb:NM_007279.1 /DB_XREF=gi:6	322,31	10,83	200,73	17,39	-1,61	-1,39	-1,89	-121,59	*
222991_s_at	ubiquilin 1	AL136643	29979	gb:AL136643.1 /DB_XREF=gi:120	1965,12	40,48	1220,91	65,16	-1,61	-1,47	-1,77	-744,21	*
1552617_a_at	ring finger and WD repeat domain 2	NM_022457	64326	gb:NM_022457.4 /DB_XREF=gi:2	887,03	8,24	552,65	13,23	-1,61	-1,54	-1,68	-334,38	*
226104_at	Ring finger protein 170	AI052736	81790	gb:AI052736 /DB_XREF=gi:3308	278,46	5,01	172,72	8,07	-1,61	-1,49	-1,76	-105,74	*
214727_at	breast cancer 2, early onset	X95152	675	gb:X95152 /DB_XREF=gi:117154	379,2	5,13	234,01	8,31	-1,62	-1,52	-1,73	-145,19	*
211080_s_at	NIMA (never in mitosis gene a)-related kina:	Z25425	4751	gb:Z25425.1 /DB_XREF=gi:40572	659,52	17,53	407,04	28,4	-1,62	-1,44	-1,84	-252,48	*
210004_at	oxidised low density lipoprotein (lectin-like)	AF035776	4973	gb:AF035776.1 /DB_XREF=gi:394	961,22	15,69	593,56	25,4	-1,62	-1,5	-1,75	-367,67	*
200695_at	protein phosphatase 2 (formerly 2A), regula	NM_014225	5518	gb:NM_014225.1 /DB_XREF=gi:7	333,99	5,87	206,41	9,5	-1,62	-1,5	-1,76	-127,58	*
200964_at	ubiquitin-activating enzyme E1 (A1S9T and I	NM_003334	7317	gb:NM_003334.1 /DB_XREF=gi:4	368,55	12,8	227,82	20,71	-1,62	-1,39	-1,92	-140,72	*
228967_at	Putative translation initiation factor	BE964053	10209	gb:BE964053 /DB_XREF=gi:1176	275,99	4,18	170,82	6,76	-1,62	-1,51	-1,74	-105,16	*
227912_s_at	Exosome component 3	AA747303	51010	gb:AA747303 /DB_XREF=gi:2787	367,07	5,15	226,73	8,34	-1,62	-1,52	-1,73	-140,34	*
1558014_s_at	male sterility domain containing 2	BG261090	84188	gb:BG261090 /DB_XREF=gi:1277	701,41	5,81	432,44	9,42	-1,62	-1,56	-1,69	-268,97	*
235428_at	Transcribed locus, strongly similar to XP_51	H78106		gb:H78106 /DB_XREF=gi:105619	372,3	5,52	229,66	8,95	-1,62	-1,52	-1,74	-142,64	*
205195_at	adaptor-related protein complex 1, sigma 1	NM_001283	1174	gb:NM_001283.1 /DB_XREF=gi:4	492,8	8,2	302,93	13,33	-1,63	-1,51	-1,76	-189,87	*
209428_s_at	zinc finger protein-like 1	BG420865	7542	gb:BG420865 /DB_XREF=gi:1332	265,29	2,37	162,35	3,87	-1,63	-1,57	-1,71	-102,94	*
215832_x_at	phosphatidylinositol binding clathrin asseml	AV722190	8301	gb:AV722190 /DB_XREF=gi:1082	490,51	13,03	300,89	21,24	-1,63	-1,45	-1,86	-189,61	*
204899_s_at	sin3-associated polypeptide, 30kDa	BF247098	8819	gb:BF247098 /DB_XREF=gi:1116	292,63	5,76	179,05	9,42	-1,63	-1,49	-1,8	-113,58	*
218261_at	adaptor-related protein complex 1, mu 2 su	NM_005498	10053	gb:NM_005498.2 /DB_XREF=gi:9	280,44	3,88	172,08	6,32	-1,63	-1,53	-1,74	-108,36	*
231850_x_at	KIAA1712	AB051499	80817	gb:AB051499.1 /DB_XREF=gi:120	772,04	6,06	473,14	9,88	-1,63	-1,57	-1,69	-298,9	*
211228_s_at	RAD17 homolog (S. pombe)	AF085736	5884	gb:AF085736.1 /DB_XREF=gi:364	543,1	6,78	330,77	11,13	-1,64	-1,55	-1,74	-212,33	*
211537_x_at	mitogen-activated protein kinase kinase kin	AF218074	6885	gb:AF218074.1 /DB_XREF=gi:674	476,8	4,94	290,1	8,12	-1,64	-1,57	-1,73	-186,69	*
210978_s_at	transgelin 2	BC002616	8407	gb:BC002616.1 /DB_XREF=gi:128	2214,82	27,29	1352	44,71	-1,64	-1,55	-1,74	-862,82	*
208456_s_at	related RAS viral (r-ras) oncogene homolog	NM_012250	22800	gb:NM_012250.1 /DB_XREF=gi:6	2585,99	11,85	1575,04	19,45	-1,64	-1,61	-1,68	-1010,95	*
222242_s_at	kallikrein 5	AF243527	25818	gb:AF243527 /DB_XREF=gi:1124	783,86	13,32	477,62	21,87	-1,64	-1,52	-1,78	-306,25	*
228124_at	chromosome 20 open reading frame 22	AW303865	26090	gb:AW303865 /DB_XREF=gi:671	262,47	6,25	160,29	10,23	-1,64	-1,47	-1,84	-102,18	*
229113_s_at	hypothetical protein FLJ31031	W16779	199990	gb:W16779 /DB_XREF=gi:12911	534,21	6,12	325,67	10,03	-1,64	-1,56	-1,73	-208,54	*
227052_at	Hypothetical protein LOC201895	AI810669	201895	gb:AI810669 /DB_XREF=gi:5397	1643,8	16,48	1004,22	26,98	-1,64	-1,56	-1,72	-639,58	*
201718_s_at	erythrocyte membrane protein band 4.1-like	BF511685	2037	gb:BF511685 /DB_XREF=gi:1159	412,31	7,4	249,71	12,23	-1,65	-1,52	-1,8	-162,6	*
210153_s_at	malic enzyme 2, NAD(+)-dependent, mitoch	M55905	4200	gb:M55905.1 /DB_XREF=gi:1872	597,6	9,84	361,81	16,26	-1,65	-1,53	-1,79	-235,79	*
230764_at	Chromosome 9 open reading frame 5	BF109998	23731	gb:BF109998 /DB_XREF=gi:1093	265,46	1,2	160,93	1,99	-1,65	-1,61	-1,69	-104,53	*
214790_at	SUMO1/sentrin specific protease 6	AK001406	26054	gb:AK001406.1 /DB_XREF=gi:70	256,93	3,29	155,58	5,43	-1,65	-1,56	-1,76	-101,36	*
219078_at	G patch domain containing 2	NM_018040	55105	gb:NM_018040.1 /DB_XREF=gi:8	292,8	6,32	177	10,46	-1,65	-1,5	-1,84	-115,8	*
225942_at	neurolysin (metallopeptidase M3 family)	AB033052	57486	gb:AB033052.1 /DB_XREF=gi:63	277,47	4,28	168,29	7,06	-1,65	-1,53	-1,78	-109,18	*
205321_at	eukaryotic translation initiation factor 2, sul	NM_001415	1968	gb:NM_001415.1 /DB_XREF=gi:4	1377,37	12	829,98	19,91	-1,66	-1,59	-1,73	-547,4	*
1554885_a_at	primase, polypeptide 2A, 58kDa	BC017833	5558	gb:BC017833.1 /DB_XREF=gi:17	454,83	5,22	274,08	8,66	-1,66	-1,57	-1,76	-180,75	*
202129_s_at	RIO kinase 3 (yeast) /// RIO kinase 3 (yeast)	AW006290	8780	gb:AW006290 /DB_XREF=gi:585	900,22	8,92	543,18	14,79	-1,66	-1,58	-1,74	-357,04	*
1554464_a_at	cartilage associated protein	BC008745	10491	gb:BC008745.1 /DB_XREF=gi:14	1284,23	11,89	775,21	19,7	-1,66	-1,59	-1,73	-509,02	*
233809_at	Huntingtin interacting protein K	AK022435	25764	gb:AK022435.1 /DB_XREF=gi:10	294,79	6,35	177,16	10,57	-1,66	-1,5	-1,86	-117,63	*
202407_s_at	PRP31 pre-mRNA processing factor 31 hom	BF342707	26121	gb:BF342707 /DB_XREF=gi:1128	336,85	8,16	203,18	13,52	-1,66	-1,48	-1,87	-133,67	*
236694_at	Chromosome Y open reading frame 15A	AW468885	246126	gb:AW468885 /DB_XREF=gi:703	417,88	6,27	251,04	10,44	-1,66	-1,55	-1,79	-166,84	*
205355_at	acyl-Coenzyme A dehydrogenase, short/bra	NM_001609	36	gb:NM_001609.1 /DB_XREF=gi:4	435,63	6,31	261,21	10,52	-1,67	-1,56	-1,79	-174,43	*
210818_s_at	BTB and CNC homology 1, basic leucine zipp	AF026199	571	gb:AF026199.1 /DB_XREF=gi:25	404,63	2,58	242,83	4,3	-1,67	-1,62	-1,72	-161,81	*
235747_at	Solute carrier family 25 (mitochondrial carri	AI305170	8034	gb:AI305170 /DB_XREF=gi:3988	309,03	10,06	185,56	16,75	-1,67	-1,43	-1,97	-123,46	*

201781_s_at	aryl hydrocarbon receptor interacting prote	AL558532	9049	gb:AL558532 /DB_XREF=gi:1290	594,33	6,41	355,98	10,71	-1,67	-1,59	-1,76	-238,35	*
201378_s_at	ubiquitin associated protein 2-like	NM_014847	9898	gb:NM_014847.1 /DB_XREF=gi:7	408,74	8,1	244,48	13,55	-1,67	-1,52	-1,85	-164,27	*
207079_s_at	mediator of RNA polymerase II transcriptior	NM_005466	10001	gb:NM_005466.1 /DB_XREF=gi:4	960,19	10,33	574,88	17,26	-1,67	-1,59	-1,76	-385,31	*
219326_s_at	UDP-GlcNAc:betaGal beta-1,3-N-acetylgluco	NM_006577	10678	gb:NM_006577.2 /DB_XREF=gi:9	271,97	8,06	162,82	13,46	-1,67	-1,46	-1,95	-109,15	*
223103_at	START domain containing 10	AF151810	10809	gb:AF151810.1 /DB_XREF=gi:492	476,81	4,35	286,21	7,25	-1,67	-1,59	-1,74	-190,61	*
212280_x_at	APG4 autophagy 4 homolog B (S. cerevisiae)	AA532726	23192	gb:AA532726 /DB_XREF=gi:2278	288,18	3,44	172,27	5,75	-1,67	-1,58	-1,78	-115,91	*
223879_s_at	oxidation resistance 1	AF309387	55074	gb:AF309387.1 /DB_XREF=gi:109	490,05	3,07	293,44	5,13	-1,67	-1,62	-1,72	-196,61	*
235559_at	hypothetical protein FLJ22374	AI539542	84182	gb:AI539542 /DB_XREF=gi:4453(	347,64	0,95	208,5	1,58	-1,67	-1,65	-1,69	-139,14	*
208232_x_at	gb:L12260.1 /DB_XREF=gi:292047 /FEA=FLn	L12260		gb:L12260.1 /DB_XREF=gi:29204	827,1	4,07	495,28	6,8	-1,67	-1,63	-1,71	-331,82	*
210186_s_at	FK506 binding protein 1A, 12kDa	BC005147	2280	gb:BC005147.1 /DB_XREF=gi:134	1717,25	16,17	1024,44	27,11	-1,68	-1,6	-1,76	-692,81	*
215708_s_at	primase, polypeptide 2A, 58kDa	AL121975	5558	gb:AL121975 /DB_XREF=gi:9663	363,42	3,18	216,01	5,35	-1,68	-1,61	-1,76	-147,41	*
209754_s_at	thymopoietin	AF113682	7112	gb:AF113682.1 /DB_XREF=gi:685	599,73	5,74	356,32	9,67	-1,68	-1,61	-1,77	-243,41	*
209488_s_at	RNA binding protein with multiple splicing	D84109	11030	gb:D84109.1 /DB_XREF=gi:1669!	311,5	0,62	185,47	1,05	-1,68	-1,66	-1,7	-126,03	*
211801_x_at	mitofusin 1	AF329637	55669	gb:AF329637.1 /DB_XREF=gi:127	539,81	9,18	320,87	15,44	-1,68	-1,55	-1,83	-218,94	*
221423_s_at	golgi membrane protein SB140 /// golgi me	NM_030799	81555	gb:NM_030799.1 /DB_XREF=gi:1	1080,28	3,75	644,7	6,28	-1,68	-1,65	-1,7	-435,59	*
202516_s_at	discs, large homolog 1 (Drosophila)	NM_004087	1739	gb:NM_004087.1 /DB_XREF=gi:4	284,38	3,19	167,84	5,41	-1,69	-1,6	-1,79	-116,54	*
213562_s_at	squalene epoxidase	BF979497	6713	gb:BF979497 /DB_XREF=gi:1234	1043,67	8,6	616,48	14,56	-1,69	-1,63	-1,77	-427,19	*
219072_at	B-cell CLL/lymphoma 7C	NM_004765	9274	gb:NM_004765.1 /DB_XREF=gi:4	558,28	6,97	330,22	11,78	-1,69	-1,59	-1,8	-228,06	*
209732_at	C-type lectin domain family 2, member B	BC005254	9976	gb:BC005254.1 /DB_XREF=gi:135	2729,5	15,37	1611,86	26,03	-1,69	-1,65	-1,74	-1117,64	*
212170_at	RNA binding motif protein 12	BF447705	10137	gb:BF447705 /DB_XREF=gi:1151	367,02	2,83	216,72	4,79	-1,69	-1,63	-1,76	-150,3	*
214493_s_at	InaD-like (Drosophila)	NM_005799	10207	gb:NM_005799.1 /DB_XREF=gi:5	294,72	6,54	174,52	11,04	-1,69	-1,52	-1,9	-120,2	*
203871_at	SUMO1/sentrin/SMT3 specific protease 3	NM_015670	26168	gb:NM_015670.1 /DB_XREF=gi:7	605,29	12,82	358,39	21,65	-1,69	-1,53	-1,89	-246,9	*
220285_at	chromosome 9 open reading frame 77	NM_016014	51104	gb:NM_016014.1 /DB_XREF=gi:7	285,77	2,19	169,5	3,69	-1,69	-1,62	-1,75	-116,27	*
234942_s_at	deoxynucleotidyltransferase, terminal, inter	AK025220	116092	gb:AK025220.1 /DB_XREF=gi:104	1194,23	9,9	706,69	16,73	-1,69	-1,62	-1,76	-487,54	*
200041_s_at	HLA-B associated transcript 1 /// HLA-B asso	NM_004640	7919	gb:NM_004640.1 /DB_XREF=gi:4	926,23	6,28	543,71	10,71	-1,7	-1,65	-1,76	-382,52	*
219015_s_at	glycosyltransferase 28 domain containing 1	NM_018466	55849	gb:NM_018466.1 /DB_XREF=gi:8	560,14	7,42	330,01	12,59	-1,7	-1,59	-1,82	-230,13	*
221966_at	chromosome 11 hypothetical protein ORF4	AA813194	56834	gb:AA813194 /DB_XREF=gi:2883	1769,24	11,64	1041,79	19,77	-1,7	-1,64	-1,76	-727,45	*
214256_at	ATPase, Class V, type 10A	AB011138	57194	gb:AB011138.1 /DB_XREF=gi:304	338,83	3,66	198,87	6,24	-1,7	-1,62	-1,8	-139,96	*
1562836_at	DEAD (Asp-Glu-Ala-Asp) box polypeptide 6	AK021715	1656	gb:AK021715.1 /DB_XREF=gi:104	264,21	2,66	154,24	4,55	-1,71	-1,63	-1,8	-109,97	*
200008_s_at	GDP dissociation inhibitor 2 /// GDP dissocia	D13988	2665	gb:D13988.1 /DB_XREF=gi:2859	3849,45	33,37	2254,34	56,98	-1,71	-1,64	-1,79	-1595,11	*
211799_x_at	Major histocompatibility complex, class I, C	U62824	3107	gb:U62824.1 /DB_XREF=gi:1575	1241,18	20,1	726,6	34,33	-1,71	-1,58	-1,86	-514,59	*
242277_at	Phosphatase and actin regulator 2	AW880875	9749	gb:AW880875 /DB_XREF=gi:804	252,42	4,4	147,61	7,53	-1,71	-1,57	-1,87	-104,81	*
203961_at	nebulette	AL157398	10529	gb:AL157398 /DB_XREF=gi:1004	277,69	3,08	162,79	5,26	-1,71	-1,61	-1,81	-114,89	*
210829_s_at	single-stranded DNA binding protein 2	AF077048	23635	gb:AF077048.1 /DB_XREF=gi:468	504,18	4,33	294,47	7,42	-1,71	-1,64	-1,79	-209,72	*
211002_s_at	tripartite motif-containing 29	AF230389	23650	gb:AF230389.1 /DB_XREF=gi:122	423,29	8,83	247,06	15,13	-1,71	-1,55	-1,91	-176,23	*
222699_s_at	pleckstrin homology domain containing, fan	BF439250	79666	gb:BF439250 /DB_XREF=gi:1145	633,23	8,42	371,33	14,37	-1,71	-1,6	-1,83	-261,9	*
1553515_at	helicase/primase complex protein	NM_138336	150678	gb:NM_138336.1 /DB_XREF=gi:1	752,07	1,74	440,69	2,98	-1,71	-1,69	-1,73	-311,39	*
239169_at	RAD52 homolog B (S. cerevisiae)	AA761980	201299	gb:AA761980 /DB_XREF=gi:2810	350,34	3,21	205,18	5,49	-1,71	-1,63	-1,79	-145,16	*
214845_s_at	calumenin	AF257659	813	gb:AF257659.1 /DB_XREF=gi:851	411,28	5,51	239,45	9,46	-1,72	-1,61	-1,84	-171,83	*
223758_s_at	general transcription factor IIH, polypeptide	BC005345	2966	gb:BC005345.1 /DB_XREF=gi:135	497,32	3,54	288,97	6,08	-1,72	-1,66	-1,79	-208,35	*
1554768_a_at	MAD2 mitotic arrest deficient-like 1 (yeast)	AF394735	4085	gb:AF394735.1 /DB_XREF=gi:252	2657,68	17,14	1542,34	29,53	-1,72	-1,67	-1,78	-1115,34	*
223115_at	cofactor required for Sp1 transcriptional act	AK001674	9440	gb:AK001674.1 /DB_XREF=gi:702	386,5	4,76	224,72	8,18	-1,72	-1,62	-1,84	-161,78	*
212528_at	DNA segment, Chr 15, Wayne State Univers	AI348009	27351	gb:AI348009 /DB_XREF=gi:4085	246,22	3,31	142,74	5,71	-1,72	-1,61	-1,85	-103,48	*
1553661_a_at	HUS1 checkpoint homolog b (S. pombe)	NM_148959	135458	gb:NM_148959.1 /DB_XREF=gi:2	254,55	2,12	148,29	3,63	-1,72	-1,65	-1,79	-106,26	*
201866_s_at	nuclear receptor subfamily 3, group C, mem	NM_000176	2908	gb:NM_000176.1 /DB_XREF=gi:4	768,56	12,15	445,37	20,96	-1,73	-1,59	-1,88	-323,2	*
225575_at	Leukemia inhibitory factor receptor	AI680541	3977	gb:AI680541 /DB_XREF=gi:4890	360,89	4,19	208,55	7,25	-1,73	-1,63	-1,84	-152,34	*
206166_s_at	chloride channel, calcium activated, family r	AF043977	9635	gb:AF043977.1 /DB_XREF=gi:513	269,11	1,75	155,96	3,02	-1,73	-1,67	-1,79	-113,16	*
212595_s_at	DAZ associated protein 2	AL534321	9802	gb:AL534321 /DB_XREF=gi:1279	978,95	10,62	565,58	18,38	-1,73	-1,64	-1,83	-413,37	*
226882_x_at	WD repeat domain 4	AI861913	10785	gb:AI861913 /DB_XREF=gi:5526	370,69	8,48	214,37	14,67	-1,73	-1,54	-1,96	-156,32	*
228824_s_at	leukotriene B4 12-hydroxydehydrogenase	BE566894	22949	gb:BE566894 /DB_XREF=gi:9810	1772,94	23,66	1021,89	41,06	-1,73	-1,62	-1,86	-751,05	*

202736_s_at	LSM4 homolog, U6 small nuclear RNA assoc	AA112507	25804	gb:AA112507 /DB_XREF=gi:1665	2381,4	27,56	1372,82	47,82	-1,73	-1,64	-1,85	-1008,59	*
213872_at	Chromosome 6 open reading frame 62	BE465032	81688	gb:BE465032 /DB_XREF=gi:9510	1089,63	12,07	629,39	20,89	-1,73	-1,64	-1,84	-460,24	*
239431_at	toll-like receptor adaptor molecule 2	AI400110	353376	gb:AI400110 /DB_XREF=gi:4243	246,82	5,93	142,68	10,25	-1,73	-1,54	-1,97	-104,15	*
241887_at	CDNA FLJ41537 fis, clone BRTHA2017985	AI370381		gb:AI370381 /DB_XREF=gi:4149	366,95	6,17	211,83	10,68	-1,73	-1,59	-1,9	-155,12	*
205627_at	cytidine deaminase	NM_001785	978	gb:NM_001785.1 /DB_XREF=gi:	240,62	7,64	137,92	13,34	-1,74	-1,49	-2,09	-102,71	*
204384_at	golgi autoantigen, golgin subfamily a, 2	NM_004486	2801	gb:NM_004486.1 /DB_XREF=gi:	316,36	3,72	182,27	6,46	-1,74	-1,63	-1,85	-134,08	*
204128_s_at	replication factor C (activator 1) 3, 38kDa	NM_002915	5983	gb:NM_002915.1 /DB_XREF=gi:	801,62	9,42	461,07	16,38	-1,74	-1,64	-1,85	-340,55	*
205071_x_at	X-ray repair complementing defective repai	AB017445	7518	gb:AB017445.1 /DB_XREF=gi:12	701,19	5,75	404,09	9,98	-1,74	-1,66	-1,81	-297,1	*
224173_s_at	mitochondrial ribosomal protein L30	BC000217	51263	gb:BC000217.1 /DB_XREF=gi:12	471,87	4,04	271,76	7,01	-1,74	-1,66	-1,82	-200,1	*
221268_s_at	sphingosine-1-phosphate phosphatase 1 ///	NM_030791	81537	gb:NM_030791.1 /DB_XREF=gi:	1218,32	8,09	699,37	14,1	-1,74	-1,68	-1,8	-518,96	*
220033_at	gb:NM_024601.1 /DB_XREF=gi:13375802 /	NM_024601		gb:NM_024601.1 /DB_XREF=gi:	270	9,04	155,5	15,69	-1,74	-1,47	-2,1	-114,5	*
213470_s_at	heterogeneous nuclear ribonucleoprotein H	BF983406	3187	gb:BF983406 /DB_XREF=gi:1238	522,59	6,83	299,13	11,93	-1,75	-1,63	-1,88	-223,45	*
1554417_s_at	anterior pharynx defective 1 homolog A (C.	AY113699	51107	gb:AY113699.1 /DB_XREF=gi:25	478,88	8,22	273,5	14,4	-1,75	-1,6	-1,92	-205,38	*
213882_at	Beta-amyloid binding protein precursor	AA012917	83941	gb:AA012917 /DB_XREF=gi:1473	253,98	5,98	145,39	10,45	-1,75	-1,55	-1,99	-108,59	*
1555679_a_at	reticulon 4 interacting protein 1	AF439711	84816	gb:AF439711.1 /DB_XREF=gi:17	252,49	3,67	144,68	6,41	-1,75	-1,62	-1,89	-107,81	*
225400_at	chromosome 1 open reading frame 19	BF111780	116461	gb:BF111780 /DB_XREF=gi:1094	432,26	10,43	246,86	18,26	-1,75	-1,55	-2	-185,4	*
1552417_a_at	neural precursor cell expressed, developme	NM_152905	121441	gb:NM_152905.1 /DB_XREF=gi:	458,2	10,13	262,15	17,71	-1,75	-1,56	-1,98	-196,05	*
214896_at	MRNA full length insert cDNA clone EUROIM	AL109671		gb:AL109671.1 /DB_XREF=gi:56	292,12	1,06	166,68	1,86	-1,75	-1,72	-1,79	-125,44	*
202604_x_at	a disintegrin and metalloproteinase domain	NM_001110	102	gb:NM_001110.1 /DB_XREF=gi:	376,18	6,49	213,98	11,4	-1,76	-1,61	-1,93	-162,19	*
203806_s_at	Fanconi anemia, complementation group A	NM_000135	2175	gb:NM_000135.1 /DB_XREF=gi:	264,38	3,58	149,98	6,32	-1,76	-1,64	-1,9	-114,4	*
201028_s_at	CD99 antigen	U82164	4267	gb:U82164.1 /DB_XREF=gi:2149	498,09	8,09	282,53	14,26	-1,76	-1,62	-1,93	-215,57	*
216765_at	Mitogen-activated protein kinase kinase 5	AK025177	5607	gb:AK025177.1 /DB_XREF=gi:10	285,34	1,05	162,08	1,84	-1,76	-1,73	-1,8	-123,26	*
211749_s_at	vesicle-associated membrane protein 3 (cell	BC005941	9341	gb:BC005941.1 /DB_XREF=gi:13	1891,39	12,39	1073,04	21,85	-1,76	-1,7	-1,83	-818,35	*
214657_s_at	Trophoblast-derived noncoding RNA	AU134977	283131	gb:AU134977 /DB_XREF=gi:109	355,57	3,75	202,55	6,59	-1,76	-1,66	-1,86	-153,02	*
1559161_at	Hypothetical protein LOC284804	AI871356	284804	gb:AI871356 /DB_XREF=gi:5545	252,14	2,09	143,08	3,68	-1,76	-1,69	-1,84	-109,06	*
1569952_x_at	Homo sapiens, Similar to syndecan binding	BC030617		gb:BC030617.1 /DB_XREF=gi:21	515,91	2,78	293,97	4,87	-1,76	-1,71	-1,81	-221,95	*
209761_s_at	SP110 nuclear body protein	AA969194	3431	gb:AA969194 /DB_XREF=gi:3144	268,82	5,84	152,07	10,32	-1,77	-1,58	-2	-116,75	*
209041_s_at	ubiquitin-conjugating enzyme E2G 2 (UBC7	BG395660	7327	gb:BG395660 /DB_XREF=gi:132	331,08	5,05	186,96	8,94	-1,77	-1,63	-1,93	-144,12	*
220183_s_at	nudix (nucleoside diphosphate linked moiet	NM_007083	11162	gb:NM_007083.1 /DB_XREF=gi:	255,92	6,32	144,32	11,21	-1,77	-1,56	-2,04	-111,6	*
91703_at	EH domain binding protein 1-like 1	AA149545	254102	Cluster Incl. AA149545:zl28g08.	233,04	6,04	131,8	10,69	-1,77	-1,55	-2,05	-101,24	*
226458_at	CDNA FLJ30340 fis, clone BRACE2007411	AL559202		gb:AL559202 /DB_XREF=gi:1290	785,99	8,04	443,34	14,25	-1,77	-1,68	-1,88	-342,65	*
216252_x_at	Fas (TNF receptor superfamily, member 6)	Z70519	355	gb:Z70519.1 /DB_XREF=gi:1418	683,52	6,96	384,02	12,4	-1,78	-1,69	-1,88	-299,5	*
1554930_a_at	fucosyltransferase 8 (alpha (1,6) fucosyltran	AB049740	2530	gb:AB049740.2 /DB_XREF=gi:18	256,49	5,17	144,48	9,18	-1,78	-1,6	-1,99	-112,02	*
207935_s_at	keratin 13	NM_002274	3860	gb:NM_002274.1 /DB_XREF=gi:	317,75	2,3	178,26	4,09	-1,78	-1,71	-1,86	-139,48	*
210318_at	retinol binding protein 3, interstitial	J03912	5949	gb:J03912.1 /DB_XREF=gi:18654	238,86	0,34	134,27	0,6	-1,78	-1,77	-1,79	-104,58	*
207827_x_at	synuclein, alpha (non A4 component of amy	L36675	6622	gb:L36675.1 /DB_XREF=gi:55621	255,35	7,58	143,46	13,48	-1,78	-1,53	-2,12	-111,9	*
228630_at	Zinc finger protein 84 (HPF2)	W92744	7637	gb:W92744 /DB_XREF=gi:14219	247,87	4,99	139,1	8,89	-1,78	-1,6	-2	-108,77	*
223847_s_at	endoplasmic reticulum-golgi intermediate c	AF267855	57222	gb:AF267855.1 /DB_XREF=gi:12	444,09	0,67	249,56	1,2	-1,78	-1,76	-1,79	-194,53	*
226002_at	GRB2-associated binding protein 1	AK022142	2549	gb:AK022142.1 /DB_XREF=gi:10	268,24	5,78	150,16	10,32	-1,79	-1,6	-2,02	-118,08	*
227905_s_at	5-azacytidine induced 2	BF000175	64343	gb:BF000175 /DB_XREF=gi:1070	392,47	10,66	218,68	19,14	-1,79	-1,56	-2,11	-173,79	*
215430_at	glycerol kinase 2	AA757089	2712	gb:AA757089 /DB_XREF=gi:2804	317,12	0,76	176,58	1,37	-1,8	-1,77	-1,82	-140,53	*
211574_s_at	membrane cofactor protein (CD46, trophob	D84105	4179	gb:D84105.1 /DB_XREF=gi:1256	342,37	10,34	189,7	18,66	-1,8	-1,54	-2,17	-152,67	*
235344_at	Protein phosphatase 1A (formerly 2C), mag	BG288054	5494	gb:BG288054 /DB_XREF=gi:1304	242,57	3,87	134,87	6,97	-1,8	-1,65	-1,97	-107,7	*
210285_x_at	Wilms tumor 1 associated protein	BC000383	9589	gb:BC000383.1 /DB_XREF=gi:12	477,25	6	264,71	10,82	-1,8	-1,68	-1,94	-212,54	*
212179_at	chromosome 6 open reading frame 111	AW157501	25957	gb:AW157501 /DB_XREF=gi:622	260,69	3,39	144,5	6,11	-1,8	-1,68	-1,94	-116,19	*
233555_s_at	sulfatase 2	AL034418	55959	gb:AL034418 /DB_XREF=gi:1154	425,19	1,44	235,83	2,59	-1,8	-1,77	-1,84	-189,35	*
238469_at	Chromosome 6 open reading frame 155	BE620374	79940	gb:BE620374 /DB_XREF=gi:9891	259,75	5,25	144,51	9,43	-1,8	-1,61	-2,02	-115,24	*
229128_s_at	Acidic (leucine-rich) nuclear phosphoprotei	AI697657	81611	gb:AI697657 /DB_XREF=gi:4985	1339,26	21,83	746,04	39,19	-1,8	-1,65	-1,97	-593,23	*
211676_s_at	interferon gamma receptor 1 /// interferon	AF056979	3459	gb:AF056979.1 /DB_XREF=gi:13	1489,99	17,77	822,64	32,19	-1,81	-1,7	-1,94	-667,35	*
214484_s_at	opioid receptor, sigma 1	AF226604	10280	gb:AF226604.1 /DB_XREF=gi:75	352,79	5,69	194,6	10,32	-1,81	-1,66	-1,99	-158,19	*

207993_s_at	calcium binding protein P22	NM_007236	11261	gb:NM_007236.1 /DB_XREF=gi:6	370,39	3,52	204,51	6,38	-1,81	-1,72	-1,91	-165,88	*
202748_at	guanylate binding protein 2, interferon-indu	NM_004120	2634	gb:NM_004120.2 /DB_XREF=gi:6	320,1	7,45	176,19	13,54	-1,82	-1,6	-2,09	-143,91	*
210154_at	malic enzyme 2, NAD(+)-dependent, mitoch	M55905	4200	gb:M55905.1 /DB_XREF=gi:1872	229,25	4,41	126,17	8,02	-1,82	-1,64	-2,04	-103,09	*
210935_s_at	WD repeat domain 1	AF274954	9948	gb:AF274954.1 /DB_XREF=gi:127	305,53	3,34	167,98	6,07	-1,82	-1,71	-1,94	-137,54	*
1555240_s_at	guanine nucleotide binding protein (G prote	AF493879	55970	gb:AF493879.1 /DB_XREF=gi:201	1389,7	17,99	764,54	32,69	-1,82	-1,69	-1,96	-625,16	*
214164_x_at	carbonic anhydrase XII	BF752277	771	gb:BF752277 /DB_XREF=gi:1207	270,24	4,51	148,03	8,24	-1,83	-1,67	-2,02	-122,2	*
216607_s_at	cytochrome P450, family 51, subfamily A, po	U40053	1595	gb:U40053 /DB_XREF=gi:118406	850,6	13,55	465,08	24,77	-1,83	-1,67	-2,01	-385,52	*
200722_s_at	membrane component, chromosome 11, su	BG258784	4076	gb:BG258784 /DB_XREF=gi:1276	958,68	6,74	523,05	12,35	-1,83	-1,76	-1,91	-435,63	*
223181_at	chromosome 18 open reading frame 55	BC000892	29090	gb:BC000892.1 /DB_XREF=gi:126	578,65	3,25	315,89	5,95	-1,83	-1,77	-1,89	-262,76	*
244107_at	gb:AW189097 /DB_XREF=gi:6463533 /DB_X	AW189097		gb:AW189097 /DB_XREF=gi:646	252,73	4,48	138,37	8,18	-1,83	-1,66	-2,03	-114,35	*
233851_s_at	torsin family 3, member A	AJ299441	64222	gb:AJ299441.1 /DB_XREF=gi:110	233,26	5,08	126,71	9,35	-1,84	-1,63	-2,1	-106,54	*
214931_s_at	SFRS protein kinase 2	AC005070	6733	gb:AC005070 /DB_XREF=gi:3406	590,11	3,38	319,5	6,25	-1,85	-1,79	-1,91	-270,61	*
218439_s_at	COMM domain containing 10	NM_016144	51397	gb:NM_016144.1 /DB_XREF=gi:7	1738,48	19,81	937,89	36,72	-1,85	-1,74	-1,99	-800,59	*
228062_at	nucleosome assembly protein 1-like 5	AW025330	266812	gb:AW025330 /DB_XREF=gi:587	244,07	4,53	131,8	8,39	-1,85	-1,67	-2,08	-112,27	*
1558105_a_at	gb:AK096921.1 /DB_XREF=gi:21756524 /TID	AK096921		gb:AK096921.1 /DB_XREF=gi:217	292,75	2,18	158,51	4,03	-1,85	-1,77	-1,93	-134,24	*
206164_at	chloride channel, calcium activated, family r	NM_006536	9635	gb:NM_006536.2 /DB_XREF=gi:1	598,85	3,09	321,78	5,75	-1,86	-1,81	-1,92	-277,07	*
224460_s_at	chromosome 14 open reading frame 160 //,	BC006117	79944	gb:BC006117.1 /DB_XREF=gi:135	289	6,4	155,37	11,9	-1,86	-1,64	-2,14	-133,64	*
201123_s_at	eukaryotic translation initiation factor 5A	NM_001970	1984	gb:NM_001970.1 /DB_XREF=gi:4	6169,96	32,09	3306,92	59,88	-1,87	-1,81	-1,93	-2863,04	*
1565681_s_at	KIAA0934	N42910	22982	gb:N42910 /DB_XREF=gi:116665	226,35	5,43	120,98	10,16	-1,87	-1,63	-2,18	-105,37	*
1555078_at	hypothetical protein MGC46336	BC036762	283933	gb:BC036762.1 /DB_XREF=gi:224	230,34	2,84	123,19	5,31	-1,87	-1,74	-2,02	-107,15	*
228851_s_at	endosulfine alpha	AV726322	2029	gb:AV726322 /DB_XREF=gi:1083	1360,3	14,91	722,91	28,06	-1,88	-1,76	-2,01	-637,39	*
206288_at	protein geranylgeranyltransferase type I, be	NM_005023	5229	gb:NM_005023.1 /DB_XREF=gi:4	273,04	6,52	144,99	12,28	-1,88	-1,64	-2,2	-128,06	*
204344_s_at	Sec23 homolog A (S. cerevisiae)	NM_006364	10484	gb:NM_006364.1 /DB_XREF=gi:5	912,4	5,17	484,34	9,73	-1,88	-1,82	-1,95	-428,06	*
220955_x_at	RAB23, member RAS oncogene family	NM_016277	51715	gb:NM_016277.1 /DB_XREF=gi:7	223	5,21	118,44	9,81	-1,88	-1,65	-2,19	-104,56	*
216495_x_at	IgG to Puumala virus G2, light chain variable	AF043584		gb:AF043584.1 /DB_XREF=gi:286	269,72	2,02	143,09	3,8	-1,88	-1,8	-1,97	-126,62	*
202376_at	serine (or cysteine) proteinase inhibitor, cla	NM_001085	12	gb:NM_001085.2 /DB_XREF=gi:9	428,8	11,74	227,31	22,14	-1,89	-1,61	-2,26	-201,49	*
201551_s_at	lysosomal-associated membrane protein 1	J03263	3916	gb:J03263.1 /DB_XREF=gi:18717	611,54	7,46	324,13	14,08	-1,89	-1,76	-2,04	-287,41	*
207175_at	adiponectin, C1Q and collagen domain cont	NM_004797	9370	gb:NM_004797.1 /DB_XREF=gi:4	242,07	0,96	128,27	1,81	-1,89	-1,84	-1,93	-113,8	*
216032_s_at	serologically defined breast cancer antigen 8	AF091085	51614	gb:AF091085.1 /DB_XREF=gi:386	1010,18	12,95	533,62	24,52	-1,89	-1,75	-2,05	-476,57	*
237108_x_at	FLJ42875 protein	AW611845	440556	gb:AW611845 /DB_XREF=gi:731	236,91	5,07	125,64	9,56	-1,89	-1,67	-2,16	-111,27	*
1565717_s_at	fusion (involved in t(12;16) in malignant lipo	BE930017	2521	gb:BE930017 /DB_XREF=gi:1045	315,01	5,49	165,82	10,42	-1,9	-1,71	-2,13	-149,19	*
216080_s_at	fatty acid desaturase 3	AC004770	3995	gb:AC004770 /DB_XREF=gi:3212	558,22	6,47	293,13	12,33	-1,9	-1,78	-2,05	-265,09	*
201120_s_at	progesterone receptor membrane compone	AL547946	10857	gb:AL547946 /DB_XREF=gi:1288	1588,65	19,21	834,47	36,57	-1,9	-1,77	-2,06	-754,18	*
220477_s_at	chromosome 20 open reading frame 30	NM_014145	29058	gb:NM_014145.1 /DB_XREF=gi:7	1113,47	7,81	587,51	14,8	-1,9	-1,82	-1,98	-525,96	*
1553603_s_at	ADP-ribosylation factor-like 6 interacting pr	NM_022374	64225	gb:NM_022374.1 /DB_XREF=gi:1	409,64	4,14	216,12	7,85	-1,9	-1,78	-2,02	-193,52	*
225341_at	transcription termination factor-like protein	BF697312	80298	gb:BF697312 /DB_XREF=gi:1198	242,77	4,09	127,49	7,79	-1,9	-1,72	-2,12	-115,27	*
200756_x_at	calumenin	U67280	813	gb:U67280.1 /DB_XREF=gi:2809	241,07	4,84	126,37	9,23	-1,91	-1,69	-2,18	-114,71	*
202145_at	lymphocyte antigen 6 complex, locus E	NM_002346	4061	gb:NM_002346.1 /DB_XREF=gi:4	456,74	7,41	239,05	14,17	-1,91	-1,73	-2,12	-217,69	*
210543_s_at	protein kinase, DNA-activated, catalytic poly	U34994	5591	gb:U34994.3 /DB_XREF=gi:1360	1948,15	23,86	1017,54	45,68	-1,91	-1,78	-2,07	-930,61	*
212214_at	optic atrophy 1 (autosomal dominant)	BF439570	4976	gb:BF439570 /DB_XREF=gi:1145	565,07	9,64	293,91	18,53	-1,92	-1,73	-2,15	-271,16	*
214149_s_at	ATPase, H+ transporting, lysosomal 9kDa, V0	AI252582	8992	gb:AI252582 /DB_XREF=gi:3849	949,89	7,62	494,85	14,63	-1,92	-1,83	-2,02	-455,04	*
210458_s_at	TRAF family member-associated NFKB activa	BC003388	10010	gb:BC003388.1 /DB_XREF=gi:130	261,04	5,8	136,29	11,11	-1,92	-1,68	-2,22	-124,75	*
231412_at	hypothetical gene supported by BX538329	H04388	441366	gb:H04388 /DB_XREF=gi:867321	298,73	3,27	155,61	6,27	-1,92	-1,8	-2,06	-143,12	*
203357_s_at	calpain 7	NM_014296	23473	gb:NM_014296.1 /DB_XREF=gi:7	631,1	9,98	327,74	19,22	-1,93	-1,75	-2,14	-303,36	*
208606_s_at	wingless-type MMTV integration site family,	NM_030761	54361	gb:NM_030761.1 /DB_XREF=gi:1	272,76	4,51	141,44	8,69	-1,93	-1,74	-2,15	-131,32	*
1552660_a_at	hypothetical protein FLJ11193	BC021215	55322	gb:BC021215.1 /DB_XREF=gi:182	322,93	4,61	167,74	8,88	-1,93	-1,76	-2,11	-155,19	*
201742_x_at	splicing factor, arginine/serine-rich 1 (splicir	NM_006924	6426	gb:NM_006924.1 /DB_XREF=gi:5	720,51	9,93	371,61	19,26	-1,94	-1,78	-2,13	-348,9	*
201337_s_at	vesicle-associated membrane protein 3 (cell	NM_004781	9341	gb:NM_004781.2 /DB_XREF=gi:9	908,32	7,74	467,84	15,03	-1,94	-1,84	-2,05	-440,47	*
223489_x_at	exosome component 3	AW015573	51010	gb:AW015573 /DB_XREF=gi:586	423,55	6,85	218,81	13,26	-1,94	-1,75	-2,16	-204,75	*
222507_s_at	chromosome 11 open reading frame 15	AI188389	56674	gb:AI188389 /DB_XREF=gi:3739	1589,94	30,98	818,4	60,18	-1,94	-1,72	-2,22	-771,54	*

231918_s_at	G elongation factor, mitochondrial 2	AK025314	84340	gb:AK025314.1 /DB_XREF=gi:104	445,7	5,66	230,18	10,95	-1,94	-1,79	-2,11	-215,52	*
206482_at	PTK6 protein tyrosine kinase 6	NM_005975	5753	gb:NM_005975.1 /DB_XREF=gi:5	253,58	1,27	129,74	2,47	-1,95	-1,89	-2,02	-123,84	*
213195_at	hypothetical protein LOC201229	AI625844	201229	gb:AI625844 /DB_XREF=gi:4650	229,73	2,25	117,7	4,39	-1,95	-1,83	-2,08	-112,02	*
1555226_s_at	chromosome 1 open reading frame 43	BC008306	25912	gb:BC008306.1 /DB_XREF=gi:14	1983,79	22,29	1010,92	43,74	-1,96	-1,83	-2,12	-972,88	*
1555814_a_at	ras homolog gene family, member A	AF498970	387	gb:AF498970.1 /DB_XREF=gi:20	3205,74	35,85	1628,32	70,57	-1,97	-1,83	-2,12	-1577,42	*
201539_s_at	four and a half LIM domains 1	U29538	2273	gb:U29538.1 /DB_XREF=gi:2078	391,63	5,02	198,72	9,89	-1,97	-1,82	-2,15	-192,91	*
216556_x_at	gb:AL135926 /DB_XREF=gi:9801286 /FEA=D	AL135926		gb:AL135926 /DB_XREF=gi:9801	1016,68	2,72	513,81	5,38	-1,98	-1,94	-2,01	-502,87	*
1552921_a_at	fidgetin-like 1	NM_022116	63979	gb:NM_022116.1 /DB_XREF=gi:1	364,89	4,79	182,95	9,56	-1,99	-1,83	-2,19	-181,94	*
207549_x_at	membrane cofactor protein (CD46, trophob	NM_002389	4179	gb:NM_002389.1 /DB_XREF=gi:1	411,06	10,49	205,02	21,04	-2	-1,71	-2,42	-206,04	*
230314_at	Similar to hypothetical protein 628	AW014557	440424	gb:AW014557 /DB_XREF=gi:586	233,51	4,78	116,93	9,54	-2	-1,75	-2,31	-116,58	*
201841_s_at	heat shock 27kDa protein 1	NM_001540	3315	gb:NM_001540.2 /DB_XREF=gi:4	1451,9	8,84	722,22	17,77	-2,01	-1,93	-2,1	-729,68	*
208047_s_at	NGFI-A binding protein 1 (EGR1 binding prot	NM_005966	4664	gb:NM_005966.1 /DB_XREF=gi:5	394,21	10,33	195,79	20,81	-2,01	-1,7	-2,45	-198,42	*
214152_at	phosphatidylinositol glycan, class B	AU144243	9488	gb:AU144243 /DB_XREF=gi:1100	204,94	3,62	101,93	7,27	-2,01	-1,79	-2,29	-103,02	*
36742_at	tripartite motif-containing 15	U34249	89870	Cluster Incl. U34249:Human put	321,47	0,74	160,14	1,48	-2,01	-1,98	-2,04	-161,32	*
213606_s_at	Rho GDP dissociation inhibitor (GDI) alpha	AI571798	396	gb:AI571798 /DB_XREF=gi:4535	249,7	2,76	123,69	5,57	-2,02	-1,88	-2,18	-126,01	*
213901_x_at	RNA binding motif protein 9	AW149379	23543	gb:AW149379 /DB_XREF=gi:619	733,03	4,85	363,49	9,78	-2,02	-1,93	-2,11	-369,54	*
219927_at	chromosome 14 open reading frame 111	NM_015962	51077	gb:NM_015962.1 /DB_XREF=gi:7	324,1	3,95	159,5	8,03	-2,03	-1,87	-2,22	-164,6	*
232640_at	COMM domain containing 5	AK023070	28991	gb:AK023070.1 /DB_XREF=gi:104	295,67	3,5	144,76	7,14	-2,04	-1,88	-2,23	-150,91	*
203865_s_at	adenosine deaminase, RNA-specific, B1 (REI	NM_015833	104	gb:NM_015833.1 /DB_XREF=gi:7	214,41	3,71	104,08	7,65	-2,06	-1,83	-2,35	-110,32	*
213887_s_at	polymerase (RNA) II (DNA directed) polypep	AI554759	5434	gb:AI554759 /DB_XREF=gi:4487	1057,93	11,2	513,91	23,05	-2,06	-1,91	-2,23	-544,02	*
206113_s_at	RAB5A, member RAS oncogene family	NM_004162	5868	gb:NM_004162.1 /DB_XREF=gi:4	377,19	4,83	183,11	9,95	-2,06	-1,89	-2,27	-194,08	*
212328_at	KIAA1102 protein	AB029025	22998	gb:AB029025.1 /DB_XREF=gi:56	537,63	23,6	259,73	48,84	-2,07	-1,56	-3,01	-277,89	*
217973_at	dicarbonyl/L-xylulose reductase	NM_016286	51181	gb:NM_016286.1 /DB_XREF=gi:7	530,59	10,99	256,83	22,71	-2,07	-1,8	-2,43	-273,76	*
221428_s_at	transducin (beta)-like 1X-linked receptor 1 /	NM_030921	79718	gb:NM_030921.1 /DB_XREF=gi:1	2293,18	22,49	1108,38	46,52	-2,07	-1,93	-2,23	-1184,8	*
201320_at	SWI/SNF related, matrix associated, actin de	BF663402	6601	gb:BF663402 /DB_XREF=gi:1193	267,19	1,96	128,25	4,08	-2,08	-1,98	-2,2	-138,94	*
222856_at	apelin, AGTRL1 ligand	AK001855	8862	gb:AK001855.1 /DB_XREF=gi:70	210,94	2,96	101,18	6,17	-2,08	-1,89	-2,32	-109,76	*
216897_s_at	hypothetical protein MGC34648	AL035309	199870	gb:AL035309.1 /DB_XREF=gi:420	204,88	2,41	98,3	5,02	-2,08	-1,92	-2,28	-106,58	*
211450_s_at	mutS homolog 6 (E. coli)	D89646	2956	gb:D89646.1 /DB_XREF=gi:2687	800,17	8,94	383,06	18,68	-2,09	-1,93	-2,28	-417,1	*
208055_s_at	hect domain and RLD 4	NM_015601	26091	gb:NM_015601.1 /DB_XREF=gi:7	382,66	5,14	182,35	10,78	-2,1	-1,91	-2,33	-200,31	*
221430_s_at	ring finger protein 146 /// ring finger protei	NM_030963	81847	gb:NM_030963.1 /DB_XREF=gi:1	227,68	3,36	108,58	7,04	-2,1	-1,89	-2,35	-119,09	*
231354_at	POU domain, class 4, transcription factor 1	AW510748	5457	gb:AW510748 /DB_XREF=gi:714	222,93	0,17	105,75	0,37	-2,11	-2,1	-2,12	-117,18	*
241656_at	Transcribed locus	AI820960		gb:AI820960 /DB_XREF=gi:5440	371,99	0,59	176,35	1,25	-2,11	-2,08	-2,13	-195,63	*
203962_s_at	nebulette	NM_006393	10529	gb:NM_006393.1 /DB_XREF=gi:5	240,7	5,42	113,71	11,47	-2,12	-1,81	-2,55	-126,99	*
1555201_a_at	chromosome 6 open reading frame 96	BC012081	55005	gb:BC012081.1 /DB_XREF=gi:150	268,32	5,78	126,4	12,26	-2,12	-1,82	-2,53	-141,92	*
242312_x_at	Transcribed locus	AV736963		gb:AV736963 /DB_XREF=gi:1085	281,14	2,78	132,55	5,9	-2,12	-1,97	-2,29	-148,59	*
1554450_s_at	hypothetical protein FLJ35954	BC041348	166968	gb:BC041348.1 /DB_XREF=gi:27	280,87	4,51	132,16	9,59	-2,13	-1,89	-2,42	-148,71	*
217202_s_at	glutamate-ammonia ligase (glutamine synth	U08626	2752	gb:U08626 /DB_XREF=gi:551473	818,16	8,27	381,47	17,74	-2,14	-1,99	-2,33	-436,69	*
1552789_at	Translocation protein 1	NM_153039	7095	gb:NM_153039.1 /DB_XREF=gi:2	217,75	3,29	101,69	7,05	-2,14	-1,92	-2,42	-116,06	*
233878_s_at	5'-3' exoribonuclease 2	BE536170	22803	gb:BE536170 /DB_XREF=gi:9764	1294,12	4,64	604,5	9,93	-2,14	-2,08	-2,2	-689,62	*
216479_at	ribosomal protein L21 /// similar to 60S ribo	AL356414	89156 /// 390	gb:AL356414 /DB_XREF=gi:1004	1227,6	1,28	574,76	2,73	-2,14	-2,12	-2,15	-652,84	*
231955_s_at	3-hydroxyisobutyrate dehydrogenase	AC007130	11112	gb:AC007130 /DB_XREF=gi:5001	1052,4	7,01	488,75	15,1	-2,15	-2,05	-2,27	-563,65	*
231723_at	sorting nexin 12	NM_013346	29934	gb:NM_013346.1 /DB_XREF=gi:7	265,55	2,35	122,59	5,09	-2,17	-2,02	-2,33	-142,96	*
1555419_a_at	N-acylsphingosine amidohydrolase (acid cer	BC016828	427	gb:BC016828.1 /DB_XREF=gi:16	514,99	5,08	236,16	11,08	-2,18	-2,02	-2,37	-278,83	*
225238_at	musashi homolog 2 (Drosophila)	BF435123	124540	gb:BF435123 /DB_XREF=gi:1144	229,01	3,73	104,17	8,21	-2,2	-1,94	-2,53	-124,84	*
206788_s_at	core-binding factor, beta subunit	AF294326	865	gb:AF294326.1 /DB_XREF=gi:98	309,57	7,64	140,37	16,85	-2,21	-1,83	-2,76	-169,21	*
211473_s_at	collagen, type IV, alpha 6	U04845	1288	gb:U04845.1 /DB_XREF=gi:4969	389,87	3,37	176,39	7,46	-2,21	-2,06	-2,38	-213,48	*
200648_s_at	glutamate-ammonia ligase (glutamine synth	NM_002065	2752	gb:NM_002065.1 /DB_XREF=gi:4	1431,51	12,2	644,96	27,09	-2,22	-2,07	-2,39	-786,55	*
231917_at	G elongation factor, mitochondrial 2	AK025314	84340	gb:AK025314.1 /DB_XREF=gi:104	604,73	6,15	271,11	13,71	-2,23	-2,06	-2,44	-333,61	*
202444_s_at	SPFH domain family, member 1	NM_006459	10613	gb:NM_006459.1 /DB_XREF=gi:5	253,82	3,24	113,16	7,27	-2,24	-2,02	-2,51	-140,66	*
1567080_s_at	ceroid-lipofuscinosis, neuronal 6, late infant	D17218	54982	gb:D17218.1 /DB_XREF=gi:5987	238,05	3,17	106,39	7,1	-2,24	-2,01	-2,52	-131,67	*

210327_s_at	alanine-glyoxylate aminotransferase (oxalos	D13368	189	gb:D13368.1 /DB_XREF=gi:2194	275,65	1,55	122,53	3,49	-2,25	-2,15	-2,36	-153,12	*
230527_at	Transcribed locus	BF224444		gb:BF224444 /DB_XREF=gi:1113	244,69	4,56	107,95	10,34	-2,27	-1,95	-2,7	-136,74	*
204427_s_at	coated vesicle membrane protein	NM_006815	10959	gb:NM_006815.1 /DB_XREF=gi:5	2731	15,13	1193,83	34,61	-2,29	-2,18	-2,4	-1537,17	*
235846_at	Fibrinogen silencer binding protein	AI208616	25788	gb:AI208616 /DB_XREF=gi:3770	383,24	19,21	167,63	43,92	-2,29	-1,58	-4,04	-215,61	*
1563445_x_at	cathepsin L-like 3	L25629	1518	gb:L25629.1 /DB_XREF=gi:40972	208,92	1,42	90,84	3,27	-2,3	-2,17	-2,45	-118,08	*
207782_s_at	presenilin 1 (Alzheimer disease 3)	NM_007319	5663	gb:NM_007319.1 /DB_XREF=gi:7	341,18	4,41	147,98	10,18	-2,31	-2,07	-2,6	-193,2	*
218045_x_at	parathymosin	NM_002824	5763	gb:NM_002824.1 /DB_XREF=gi:4	215,19	1,43	93,33	3,29	-2,31	-2,18	-2,45	-121,86	*
215113_s_at	SUMO1/sentrin/SMT3 specific protease 3	AK000923	26168	gb:AK000923.1 /DB_XREF=gi:70	210,22	3,46	90,82	8,01	-2,31	-2,02	-2,71	-119,39	*
236444_x_at	hypothetical LOC389328	BE785577	389328	gb:BE785577 /DB_XREF=gi:1020	290,69	0,17	125,34	0,39	-2,32	-2,31	-2,33	-165,35	*
203811_s_at	DnaJ (Hsp40) homolog, subfamily B, membe	NM_007034	11080	gb:NM_007034.2 /DB_XREF=gi:6	587,69	8,31	252,46	19,35	-2,33	-2,06	-2,67	-335,24	*
234728_s_at	DEAH (Asp-Glu-Ala-His) box polypeptide 35	AL023803	60625	gb:AL023803 /DB_XREF=gi:9408	473,44	1,16	201,58	2,71	-2,35	-2,3	-2,4	-271,86	*
215707_s_at	prion protein (p27-30) (Creutzfeld-Jakob dis	AV725328	5621	gb:AV725328 /DB_XREF=gi:1083	1080,78	7,31	456,28	17,31	-2,37	-2,23	-2,53	-624,5	*
224559_at	metastasis associated lung adenocarcinoma	AF001540	378938	gb:AF001540 /DB_XREF=gi:2529	2456,45	20,36	1037,36	48,21	-2,37	-2,2	-2,57	-1419,1	*
1554397_s_at	ubiquitin-conjugating enzyme E2-like	BC011011	55293	gb:BC011011.1 /DB_XREF=gi:15	485,41	6,94	203,92	16,51	-2,38	-2,1	-2,75	-281,49	*
234724_x_at	gb:AF152528.1 /DB_XREF=gi:5457102 /FEA=	AF152528		gb:AF152528.1 /DB_XREF=gi:545	206,09	0,15	85,98	0,35	-2,4	-2,38	-2,41	-120,11	*
1568964_x_at	sialophorin (gpL115, leukosialin, CD43)	BC035510	6693	gb:BC035510.1 /DB_XREF=gi:23	594,07	1,76	246,46	4,25	-2,41	-2,34	-2,48	-347,61	*
1553694_a_at	phosphoinositide-3-kinase, class 2, alpha po	NM_002645	5286	gb:NM_002645.1 /DB_XREF=gi:4	269,94	2,92	111,77	7,05	-2,42	-2,18	-2,7	-158,17	*
220892_s_at	phosphoserine aminotransferase 1	NM_021154	29968	gb:NM_021154.1 /DB_XREF=gi:1	292,63	3,05	120,74	7,4	-2,42	-2,2	-2,7	-171,89	*
234726_s_at	hypothetical protein FLJ13576	AK025482	64418	gb:AK025482.1 /DB_XREF=gi:10	252,95	3,39	103,36	8,29	-2,45	-2,16	-2,82	-149,59	*
214895_s_at	a disintegrin and metalloproteinase domain	AU135154	102	gb:AU135154 /DB_XREF=gi:1099	618,36	4,22	245,28	10,64	-2,52	-2,35	-2,72	-373,08	*
1553112_s_at	cyclin-dependent kinase 8	NM_001260	1024	gb:NM_001260.1 /DB_XREF=gi:4	242,22	3,87	96,22	9,75	-2,52	-2,15	-3,03	-146,01	*
224117_at	hypothetical gene supported by BC001801	BC001801	284912	gb:BC001801.1 /DB_XREF=gi:12	356,39	1,55	141,67	3,9	-2,52	-2,41	-2,64	-214,72	*
202278_s_at	serine palmitoyltransferase, long chain base	NM_006415	10558	gb:NM_006415.1 /DB_XREF=gi:5	334,57	2,75	132	6,97	-2,53	-2,33	-2,78	-202,56	*
218748_s_at	SEC10-like 1 (S. cerevisiae)	NM_006544	10640	gb:NM_006544.1 /DB_XREF=gi:5	402,63	4,57	158,9	11,57	-2,53	-2,26	-2,88	-243,73	*
229930_at	hypothetical LOC150371	Z83851	150371	gb:Z83851 /DB_XREF=gi:544134	174,18	2,21	68,94	5,58	-2,53	-2,23	-2,92	-105,24	*
244030_at	gb:BG390493 /DB_XREF=gi:13283941 /DB_	BG390493		gb:BG390493 /DB_XREF=gi:1328	213,08	2,66	83,18	6,82	-2,56	-2,25	-2,96	-129,9	*
207001_x_at	TSC22 domain family 3	NM_004089	1831	gb:NM_004089.1 /DB_XREF=gi:4	724,16	3,92	281,69	10,07	-2,57	-2,43	-2,73	-442,46	*
226051_at	selenoprotein M	BF973568	140606	gb:BF973568 /DB_XREF=gi:1234	218,7	5,06	85,22	12,99	-2,57	-2,04	-3,43	-133,48	*
203032_s_at	fumarate hydratase	AI363836	2271	gb:AI363836 /DB_XREF=gi:4123	482,6	3,18	187,05	8,21	-2,58	-2,4	-2,78	-295,56	*
202833_s_at	serine (or cysteine) proteinase inhibitor, cla	NM_000295	5265	gb:NM_000295.1 /DB_XREF=gi:4	842,22	4,68	324,05	12,15	-2,6	-2,45	-2,77	-518,17	*
217139_at	gb:AJ002428 /DB_XREF=gi:3183956 /FEA=D	AJ002428		gb:AJ002428 /DB_XREF=gi:3183	247,75	2,07	95,32	5,39	-2,6	-2,38	-2,87	-152,43	*
222846_at	RAB8B, member RAS oncogene family	AB038995	51762	gb:AB038995.1 /DB_XREF=gi:72	201,81	2,46	76,77	6,47	-2,63	-2,31	-3,06	-125,04	*
213555_at	RWD domain containing 2	AL049699	112611	gb:AL049699 /DB_XREF=gi:5419	216,16	0,6	81,52	1,59	-2,65	-2,57	-2,74	-134,64	*
217729_s_at	amino-terminal enhancer of split	NM_001130	166	gb:NM_001130.3 /DB_XREF=gi:6	268,22	6	100,19	16,06	-2,68	-2,11	-3,64	-168,03	*
209629_s_at	nuclear transport factor 2-like export factor	AF201942	55916	gb:AF201942.1 /DB_XREF=gi:92	212,56	3,78	79,04	10,16	-2,69	-2,21	-3,42	-133,53	*
205721_at	GDNF family receptor alpha 2	U97145	2675	gb:U97145.1 /DB_XREF=gi:2282	251,89	0,08	93,08	0,23	-2,71	-2,7	-2,72	-158,81	*
215329_s_at	cell division cycle 2-like 1 (PITSLRE proteins)	AL031282	984 /// 985	gb:AL031282 /DB_XREF=gi:3860	294,6	2,81	107,74	7,68	-2,73	-2,44	-3,1	-186,85	*
206667_s_at	secretory carrier membrane protein 1	AF005037	9522	gb:AF005037.1 /DB_XREF=gi:22	370,79	3,12	134,33	8,6	-2,76	-2,49	-3,09	-236,46	*
223341_s_at	short coiled-coil protein	AF330205	60592	gb:AF330205.1 /DB_XREF=gi:12	1258,69	8,16	456,44	22,5	-2,76	-2,55	-3	-802,25	*
221618_s_at	TAF9-like RNA polymerase II, TATA box bind	AF220509	51616	gb:AF220509.1 /DB_XREF=gi:99	211,51	3,03	76,04	8,42	-2,78	-2,35	-3,41	-135,47	*
223778_at	kinesin family member 9	AF311212	64147	gb:AF311212.1 /DB_XREF=gi:11	277,61	0,08	99,21	0,23	-2,8	-2,79	-2,81	-178,4	*
224407_s_at	Mst3 and SOK1-related kinase /// Mst3 and	AF344882	51765	gb:AF344882.1 /DB_XREF=gi:13	240,13	2,22	85,41	6,25	-2,81	-2,51	-3,2	-154,71	*
215236_s_at	phosphatidylinositol binding clathrin asseml	AV721177	8301	gb:AV721177 /DB_XREF=gi:1081	200,18	2,67	69,85	7,66	-2,87	-2,42	-3,5	-130,33	*
218922_s_at	LAG1 longevity assurance homolog 4 (S. cer	NM_024552	79603	gb:NM_024552.1 /DB_XREF=gi:1	205,62	2,16	71,66	6,21	-2,87	-2,51	-3,35	-133,96	*
222719_s_at	platelet derived growth factor C	AB033831	56034	gb:AB033831.1 /DB_XREF=gi:93	246,01	4,56	84,97	13,2	-2,9	-2,3	-3,89	-161,04	*
211559_s_at	cyclin G2	L49506	901	gb:L49506.1 /DB_XREF=gi:12362	374,78	2,77	124,06	8,36	-3,02	-2,72	-3,4	-250,72	*
202917_s_at	S100 calcium binding protein A8 (calgranulir	NM_002964	6279	gb:NM_002964.2 /DB_XREF=gi:9	262,67	1,4	85,01	4,34	-3,09	-2,85	-3,37	-177,66	*
1555326_a_at	a disintegrin and metalloproteinase domain	AF495383	8754	gb:AF495383.1 /DB_XREF=gi:21	553,56	2,3	177,14	7,19	-3,13	-2,93	-3,35	-376,43	*
203289_s_at	chromosome 16 open reading frame 35	BE791629	8131	gb:BE791629 /DB_XREF=gi:1021	201,93	1,18	63,44	3,76	-3,18	-2,9	-3,53	-138,49	*
211194_s_at	tumor protein p73-like	AB010153	8626	gb:AB010153.1 /DB_XREF=gi:34	548,29	3,49	164,7	11,62	-3,33	-2,98	-3,77	-383,59	*

231128_at	hypothetical protein MGC33846	BE858471	220382	gb:BE858471 /DB_XREF=gi:1037	208,96	0,64	56,81	2,36	-3,68	-3,44	-3,95	-152,16	*
204426_at	coated vesicle membrane protein	NM_006815	10959	gb:NM_006815.1 /DB_XREF=gi:5	1454,88	2,82	375,36	10,94	-3,88	-3,7	-4,07	-1079,52	*
226987_at	RNA binding motif protein 15B	W68720	29890	gb:W68720 /DB_XREF=gi:13776	503,68	7,5	85,3	44,31	-5,91	-3,18	-40,59	-418,39	*
239928_at	hypothetical protein FLJ12975	AI733451	79867	gb:AI733451 /DB_XREF=gi:5054	241,23	1,63	35,47	11,08	-6,8	-4,49	-13,98	-205,76	*
1554947_at	acetyl-Coenzyme A acetyltransferase 1 (ace	BC010942	38	gb:BC010942.1 /DB_XREF=gi:15	210,25	0,27	17,12	3,31	-12,28	-9,32	-17,99	-193,13	*
241245_at	Transcribed locus, weakly similar to XP_496	AV647470		gb:AV647470 /DB_XREF=gi:9868	3319,72	0,08	98,86	2,54	-33,58	-32,22	-35,06	-3220,86	*
1566900_at	TGFB-induced factor (TALE family homeobo	AL832409	7050	gb:AL832409.1 /DB_XREF=gi:217	1822,73	1,04	21	90,18	-86,82	-10,76	-100000000	-1801,73	*
234380_x_at	hypothetical LOC389253	M18679	389253	gb:M18679 /DB_XREF=gi:33737	2548,57	0,14	21,99	16,66	-115,9	-51,6	-100000000	-2526,58	*
207412_x_at	gb:NM_001808.1 /DB_XREF=gi:4502772 /G	NM_001808		gb:NM_001808.1 /DB_XREF=gi:4	5888,33	0,02	4,04	22,57	-1458,05	-143,06	-100000000	-5884,29	*
228770_at	G protein-coupled receptor 146	BE645027	115330	gb:BE645027 /DB_XREF=gi:9969	4977,54	0	1	3,88	-4977,54	-673,96	-100000000	-4976,54	*
